# DeepPod: a convolutional neural network based quantification of fruit number in *Arabidopsis*

**DOI:** 10.1093/gigascience/giaa012

**Published:** 2020-03-04

**Authors:** Azam Hamidinekoo, Gina A Garzón-Martínez, Morteza Ghahremani, Fiona M K Corke, Reyer Zwiggelaar, John H Doonan, Chuan Lu

**Affiliations:** 1 Department of Computer Science, Aberystwyth University, Aberystwyth, Ceredigion SY233DB, UK; 2 National Plant Phenomics Centre, Institute of Biological, Environmental and Rural Sciences, Aberystwyth University, Aberystwyth, Ceredigion SY233EB, UK

**Keywords:** plant phenotyping, image analysis, deep learning, object detection, fruit counting, *Arabidopsis*

## Abstract

**Background:**

High-throughput phenotyping based on non-destructive imaging has great potential in plant biology and breeding programs. However, efficient feature extraction and quantification from image data remains a bottleneck that needs to be addressed. Advances in sensor technology have led to the increasing use of imaging to monitor and measure a range of plants including the model *Arabidopsis thaliana*. These extensive datasets contain diverse trait information, but feature extraction is often still implemented using approaches requiring substantial manual input.

**Results:**

The computational detection and segmentation of individual fruits from images is a challenging task, for which we have developed DeepPod, a patch-based 2-phase deep learning framework. The associated manual annotation task is simple and cost-effective without the need for detailed segmentation or bounding boxes. Convolutional neural networks (CNNs) are used for classifying different parts of the plant inflorescence, including the tip, base, and body of the siliques and the stem inflorescence. In a post-processing step, different parts of the same silique are joined together for silique detection and localization, whilst taking into account possible overlapping among the siliques. The proposed framework is further validated on a separate test dataset of 2,408 images. Comparisons of the CNN-based prediction with manual counting (*R*^2^ = 0.90) showed the desired capability of methods for estimating silique number.

**Conclusions:**

The DeepPod framework provides a rapid and accurate estimate of fruit number in a model system widely used by biologists to investigate many fundemental processes underlying growth and reproduction

## Introduction

Photometrics (imaging followed by computationally assisted feature extraction and measurement) promises to revolutionize biological research and agricultural production systems [1–5]. Automation of workflows remains a key challenge in the scaling of these approaches to cope with the requirements of large genetic experiments or, indeed, food production systems. Phenotyping aims to measure observable plant features, often as a response to environmental cues and/or variability between individuals. Traditionally, phenotyping has been a labour-intensive and costly process, usually manual and often destructive. High-throughput phenotyping technologies aim to address this problem by the use of non-destructive approaches either in glasshouses [1, 2, 6] or directly in the field [4, 7] integrating imaging, robotics, spectroscopy, high-tech sensors, and high-performance computing [3, 8].

Imaging has the potential to generate an enormous volume of data in real time, while image analysis to extract useful information is currently the main bottleneck. The extraction of quantitative traits relies on the development and use of improved software techniques. Machine learning tools have been used to identify patterns in large biological datasets [8–12]. Recently, deep learning tools have been applied to accurately extract features from plant images [13–21].

Model organisms have been widely used to dissect different biological processes and provide a useful means to test and develop new methods that can subsequently be more widely applied to crop and ecological scenarios. *Arabidopsis thaliana* is a small, flowering plant widely used to address questions related to plant genetics, molecular biology, evolution, ecology, and physiology, among others [22–24]. The seedling produces a small rosette that increases in size by addition of leaves. The central meristem produces an inflorescence that produces flowers and then fruits. The fruits are also known as pods or siliques [24]. The measurement of traits, such as growth rate, flowering, and fruit number, is key to evaluate plant performance and reproductive fitness [25]. However, many high-throughput imaging studies focus on growth dynamics of the rosette [9, 26–28], despite the importance of fruit production in reproductive and evolutionary processes [2, 29–31].

This work demonstrates that deep learning can be used to estimate fruit number from images. In particular, we have developed DeepPod, a framework for *Arabidopsis* silique detection that involves a deep neural network for patch-based classification and an object reconstructor for silique localization and counting. The framework has been validated using a separate dataset of 2,408 images from biological experiments. This allowed the analysis of large numbers of plants' inflorescences in an accurate and effective way, providing a cost-effective alternative to manual counting.

## Background

Convolutional neural networks (CNNs) have become the dominant type of models for image classification [32]. The input for a CNN, typically an image, can be represented as a 3D array of height × width × channels. A CNN contains convolutional layers, where inputs are passed through various filters for extracting features that are arranged as feature maps, prior to using the fully connected layers for classification or regression. The weights or parameters of the filters are shared among the neurons of the convolutional layers [33], not only to encourage detection of repeated patterns in the image but also to reduce the number of parameters for the network to learn. Other types of layers such as pooling are also often used in combination with convolutional layers to reduce the dimensionality of feature maps. A CNN can be trained using a back-propagation algorithm to update the weights in an iterative process, in order to minimize the loss function that measures the discrepancy between the predicted output and actual output for the training examples. What makes CNNs particularly attractive in computer vision is that they can directly extract features from images without the need for time-consuming, hand-crafted pre-processing or feature extraction steps, unlike classical machine learning approaches [34].

Recent publications have reported the application of deep learning in various plant phenotyping tasks such as leaf counting, age estimation, mutant classification, disease detection, fruit classification, and plant organ localization [13–16, 18–21]. Mohanty et al. [14] trained deep CNNs to identify 14 crop species and 26 diseases using a publicly available plant disease dataset. They built models with architectures of AlexNet [35] and GoogleNet [36] using transfer learning. Wang et al. [20] used CNNs to establish disease severity in apple black rot images. Deep learning meta-architectures have also been considered for more complex scenarios. Fuentes et al. [19] demonstrated a combination of CNNs and deep feature extractors to recognize different diseases and pests in tomatoes, which dealt with inter- and intra-class variations. Deep learning was also used for cassava disease detection via mobile devices [21]. Pawara et al. [18] applied CNNs to classify leaves, fruits, and flowers in field images. They compared the performance of classical classifiers to CNNs, where architectures such as GoogleNet and AlexNet gave the best results in the plant-related datasets used. Namin et al.[16] proposed a convolutional neural network–long short-term memory (CNN-LSTM) framework for plant classification using temporal sequence of images. In particular, the model features were learned using CNNs and the plant growth variation over time was modeled with LSTMs. Ubbens et al. [15] used CNNs for regression to perform leaf counting. They used rendered images of synthetic plants to augment an *Arabidopsis* rosette dataset and concluded that the augmentation with high-quality 3D synthetic plants improved the performance of leaf counting while real and synthetic plants could be interchangeably used for training a neural network. Pound et al. [13] demonstrated wheat root and shoot feature identification and localization using 2 different standard CNN architectures for patch classification. For shoot features, they found that the leaf tips represented the hardest classification problem compared to the leaf base owing to the existing variations in orientation, size, shape, and colour of tips in their dataset. Further reconstruction from the classification results of the overlapping patches allows localization of separate structural regions such as leaf tips and bases. However the objects of interest as a whole (such as leaves) are yet to be identified in order to extract more morphological features (e.g., leaf length and shape).

Our proposed framework treats the silique (or pod) counting problem as an object detection and segmentation problem followed by counting. One popular approach of deep learning frameworks for object detection is to train a single CNN to jointly perform object classification and localization tasks, where the object localizations are usually defined by bounding boxes. Examples of such networks include Fast-RCNN (regional-CNN), SSD (single shot multibox detector), and YOLO (you only look once) [37]. However training of such networks requires labelled data with detailed segmentation or bounding boxes of individual objects, which are usually obtained through a tedious manual process. Moreover, the image size allowed for the network input is limited owing to the complexity of network architecture and the available memory.

In our case, the resolution of the raw images needs to be sufficiently high in order to preserve details of pods that are small and narrow, often overlapping. A single image can also contain a wide variation in the number of fruits from 0 to near 400, which poses further challenges for deep learning models when the available labelled data are limited.

To address these issues, we adopted an alternative approach that performs patch-based classification and localization in 2 separate phases. The first step is to classify a region of a suitable size in the original image into different parts of the inflorescences. In the localization phase, each original image will be scanned and each patch classified as silique/not silique (i.e., as 1 of the 4 classes including the tip, base, or body of siliques, and the stem inflorescence). Given an accurate classification of patches as silique/not silique, one could then estimate the number of siliques and their lengths to a good precision. The manual annotation task for the proposed framework was simple, involving collection of sufficient pixels from different defined structural parts of the plant.

## Data Acquisition

A set of 2,552 images of mature inflorescences taken from a subset of the Multiparent Advanced Generation Inter-Cross (MAGIC) RIL (Recombinant Inbred Line) population [38] were used to establish and test the CNN pipeline. A subset (referred to as Set-1 = 144 images) of this dataset was randomly selected for manual annotation and then used to train 1 shallow and 1 deep CNN. A total of 2,408 images (referred to as Set-2) were used to test the performance of the selected model. Information about the dedicated data for different tasks is presented in Table 1.

**Table 1.** Information about the dedicated data for different tasks

Dataset name	No. of images	Provided annotation	Used task
Set-1	144	Silique main structural elements, silique count	Developing classification model, developing counting pipeline
Set-2	2,408	Silique count	Evaluating counting pipeline

Plants were grown on an automatic watering platform within the National Plant Phenomics Centre (NPPC) (Aberystwyth University, UK) in 6-cm diameter pots half-filled with vermiculite; the upper half was filled with 30% grit/sand: 70% Levington F1 (peat-based compost). The vermiculite was used to restrict plant growth. Pots were filled to a uniform weight. Each plant was automatically weighed and irrigated from above to a 75% gravimetric water content daily.

**Figure 1:**
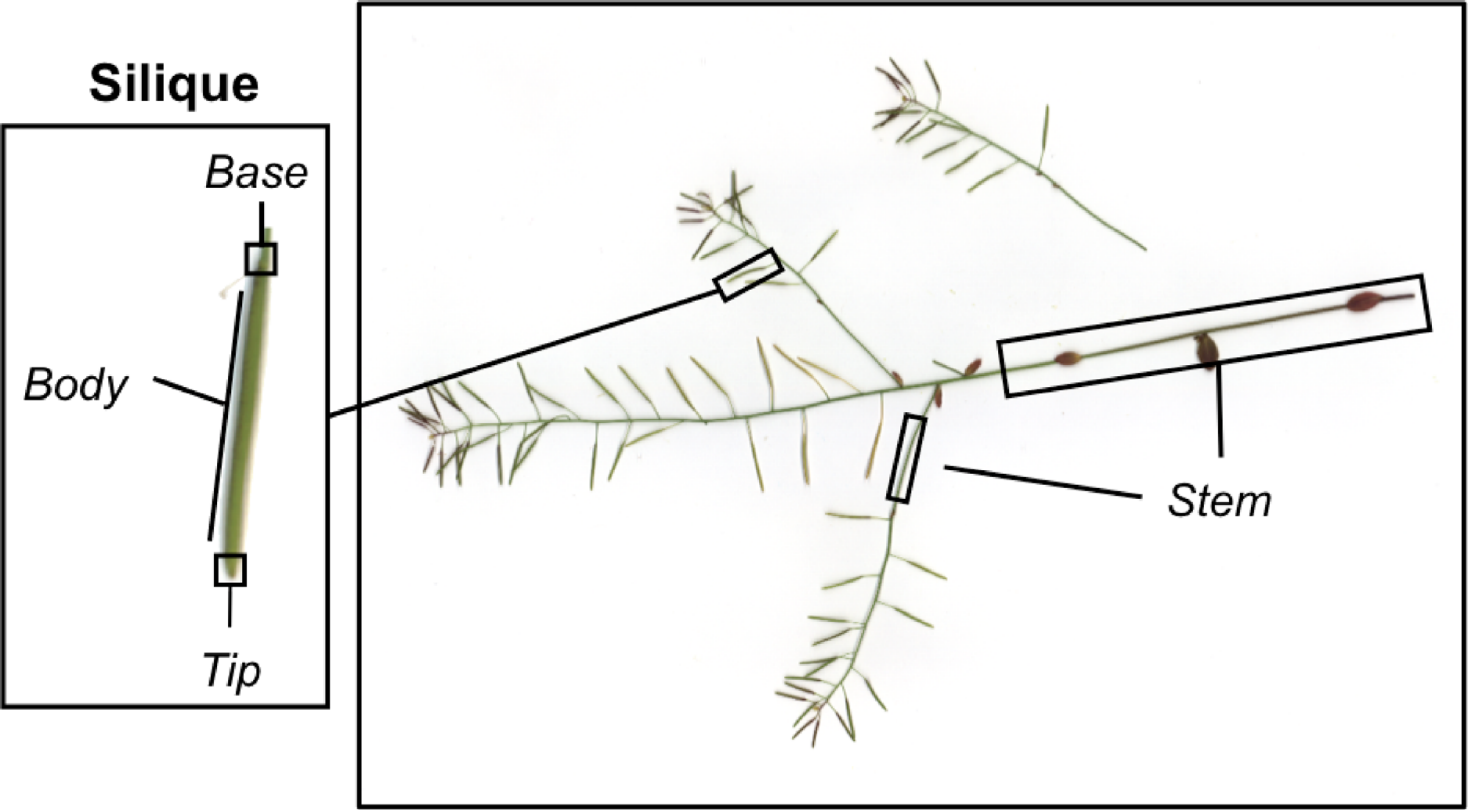
An illustrative example of image and features (the important parts of the plants) annotated for patch-based classification.

The mature inflorescence or stem of each plant, with attached fruits, was harvested and imaged in a flatbed scanner (Plustek, OpticPro A320, Ewige Weide 13 22926 Ahrensburg, Germany). Images were saved at 300 dpi and stored in .PNG format with image size equal to 3,600 × 5,100 pixels. The image file name includes the identification number for the line (e.g., ATxxx_001xxx represents RIL001) according to Kover et al. [38]. A sample image is shown in Fig. 1. Manual counting of viable fruits in images was undertaken by a single person to minimize operator variation. ImageJ [39] was used to track the counting by setting a label to each fruit as it was counted.

## Patch-based Classification using CNNs

### Data preparation for model development

#### Data annotation

**Figure 2:**
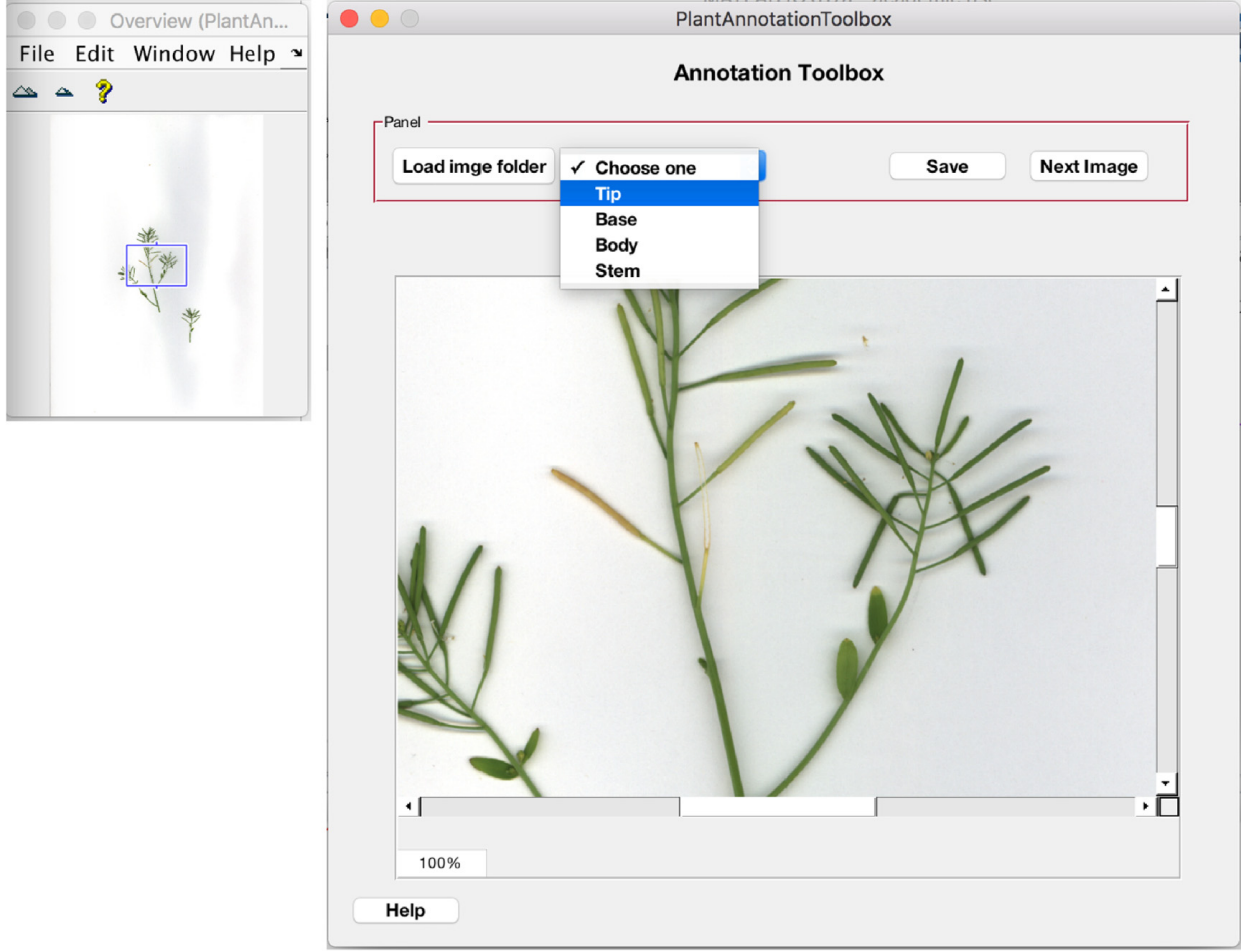
The developed GUI used for manually annotating plant parts.

**Figure 3:**
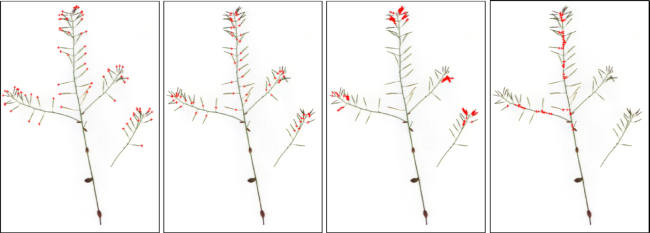
Example annotated images (from left to right) for tip, base, body, and stem. Our annotation approach only requires sampling of pixels/points for the 4 main structural regions. Although most tips and bases have been annotated (see the left 2 panels), only a small portion of points for body or stems have been sampled and labelled (see the right 2 panels).

An annotation tool with a GUI was built (in MATLAB) to assist with manual annotation of different parts of the inflorescence. Fig. 2 shows the schematic of this GUI with some screenshots of annotation. The user selects the class type (tip, base, body of the silique, and stem) and clicks on the respective parts on each input image. The annotated parts (points clicked) were saved as defined locations based on image coordinates. An example annotated image illustrating the predefined parts of the silique (tip, base, body, and stem) is given in Fig. 3. This tool was used to manually annotate Set-1, which was used to develop the patch classifiers (see Section Patch-Based Classification Problem).

The main advantages of this annotation platform include its relatively low cost and ease of use. Compared to other annotation approaches that require detailed segmentation, polygons, or bounding boxes, this approach requires annotation of just 4 main structural elements. Using this platform, Set-1 was manually annotated by a single person in a total of 36 working hours.

Table 2 shows the number of annotations performed per class (before augmentation). This dataset was used in the initial training step for classifying whole inflorescences into defined parts. To prepare patches for classification, Set-1 was randomly split into training, validation, and test sets as rounded of 65%, 20%, and 15% of the 144 images.

**Table 2.** Summary statistics for data annotation performed on Set-1

Feature	No. of manual annotations
Silique tip	7,299
Silique base	8,058
Silique body	11,187
Stem	10,266

#### Patch generation and augmentation

**Figure 4:**
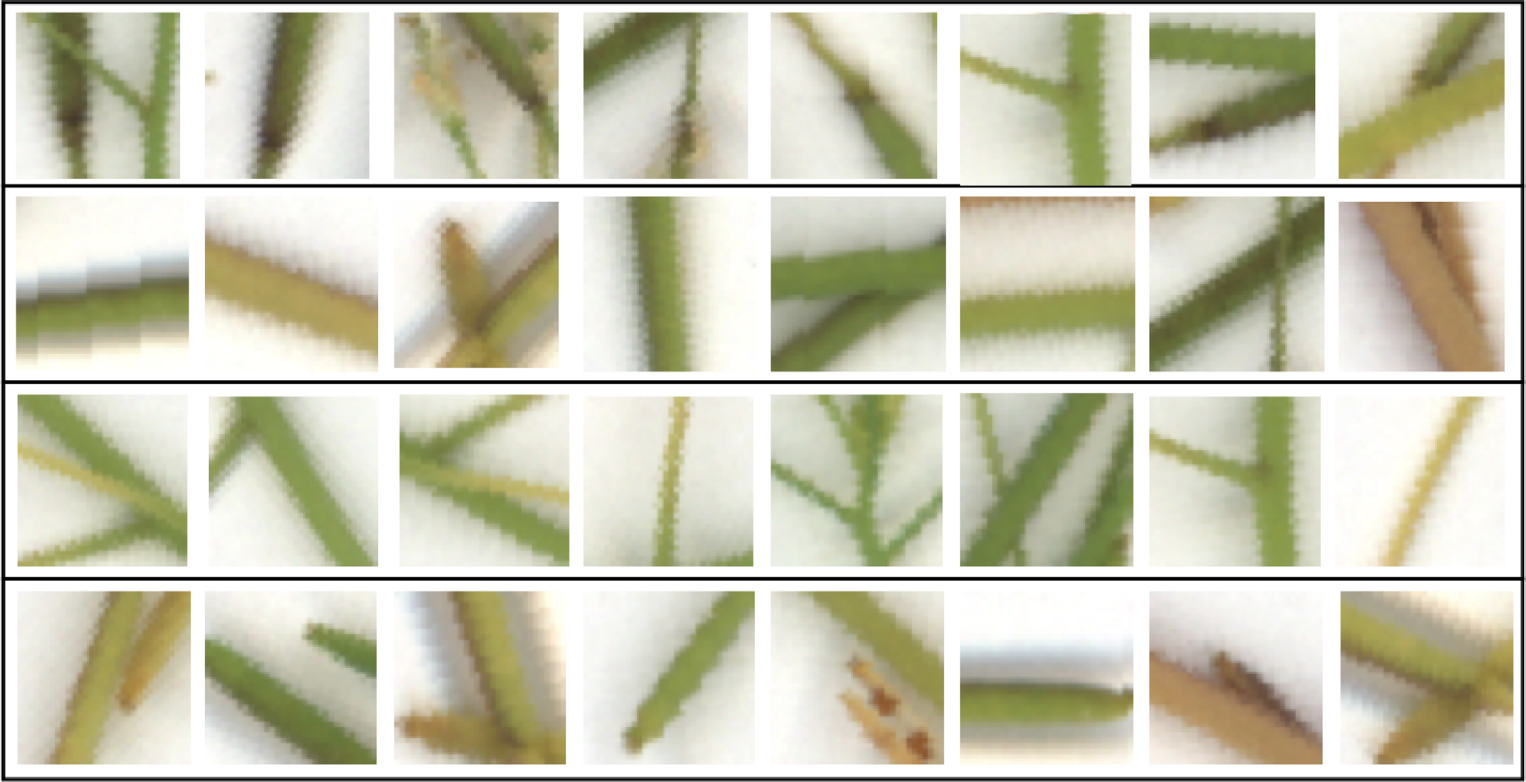
Example extracted patches using manual annotations. *From top to bottom:* samples of base, body, stem, and tip, respectively. Note that the key structural elements are not always centred in the patches as a result of the random cropping process for patch extraction.

An approach similar to that proposed by Pound et al. [13] has been followed for image patch generation and augmentation. Using the annotated data to prepare training samples, square bounding box patches were extracted while being centred at the manually annotated points. Subsequently, data augmentation [40, 41] was performed to increase the amount of training data via specific transformations, as well as considering frames different from the centred ones. The patches of size 50 × 50 were first extracted. Then, random 32 × 32 pixel crops followed by random mirroring or rotation were performed. For pre-processing, we normalized the data using the channel means and standard deviations on the training set. For validation samples, no augmentation was undertaken and the 32 × 32 patches centred at the annotated points were extracted. Fig. 4 shows various examples of each class that were used in the training procedure.

### Data preparation for testing

The training patches were centred at the annotated points; this was followed by augmentation, as described earlier. To prepare test samples, the difference in the pixel intensity distribution between the testing data and the training/validation data (that were used during training time) was taken into account. First, the whole image was scanned over with a sliding window and tiled into 32 × 32 patches with 50% overlap in both the vertical and horizontal directions (see Fig. 7). Most pixels within the area of interest (plant area) would hence be included in 4 different patches. The patches belonging to the white background (lacking plant pixels) were excluded by thresholding.

The rationale behind selecting overlapping regions was (i) to increase the number of patches by a factor of 4 compared to without overlapping and (ii) to make the patch classification more robust by combining multiple predictions.

When the model is applied to test data, the difference between the sample distribution for training and that for testing, i.e., presence of potential covariate and dataset shift, can adversely affect the model generalization performance. To address this issue, each test image was also to be normalized using the channel-wise mean and standard deviation of the training set.

Then the resultant patches were fed to the trained networks and the classification outcomes for each sample patch (tip, base, stem, body) were computed.

### Building CNN classifiers

In the next step, CNN-based classifiers were built to take extracted patches of interest as input, and to output probability scores for different labels {0, 1, 2, 3} indicating the probability that the input patch contains a base, body, stem, and tip, respectively.

#### Network architecture

**Figure 5:**
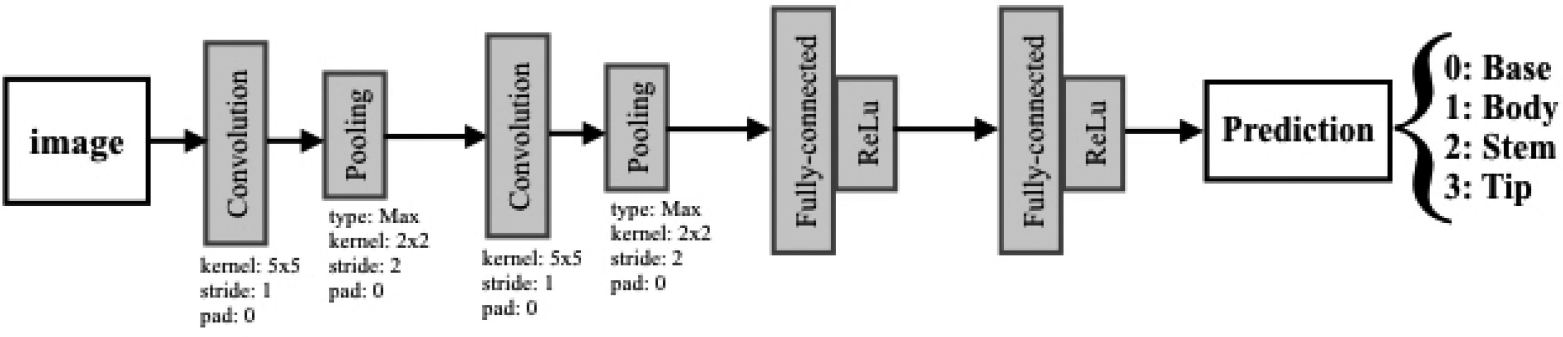
LeNet architecture.

LeNet is a pioneering CNN that was proposed to classify handwriting digits [42]. LeNet's architecture [43] consists of 2 sets of convolutional and pooling layers stacked on top of each other, followed by 2 fully connected layers and finally ending with a Softmax layer (see Fig. 5). LeNet is a simple shallow network and has been chosen as a baseline model in this study, considering the potentially higher computational resource needs for running more complex deep learning models.

**Figure 6:**
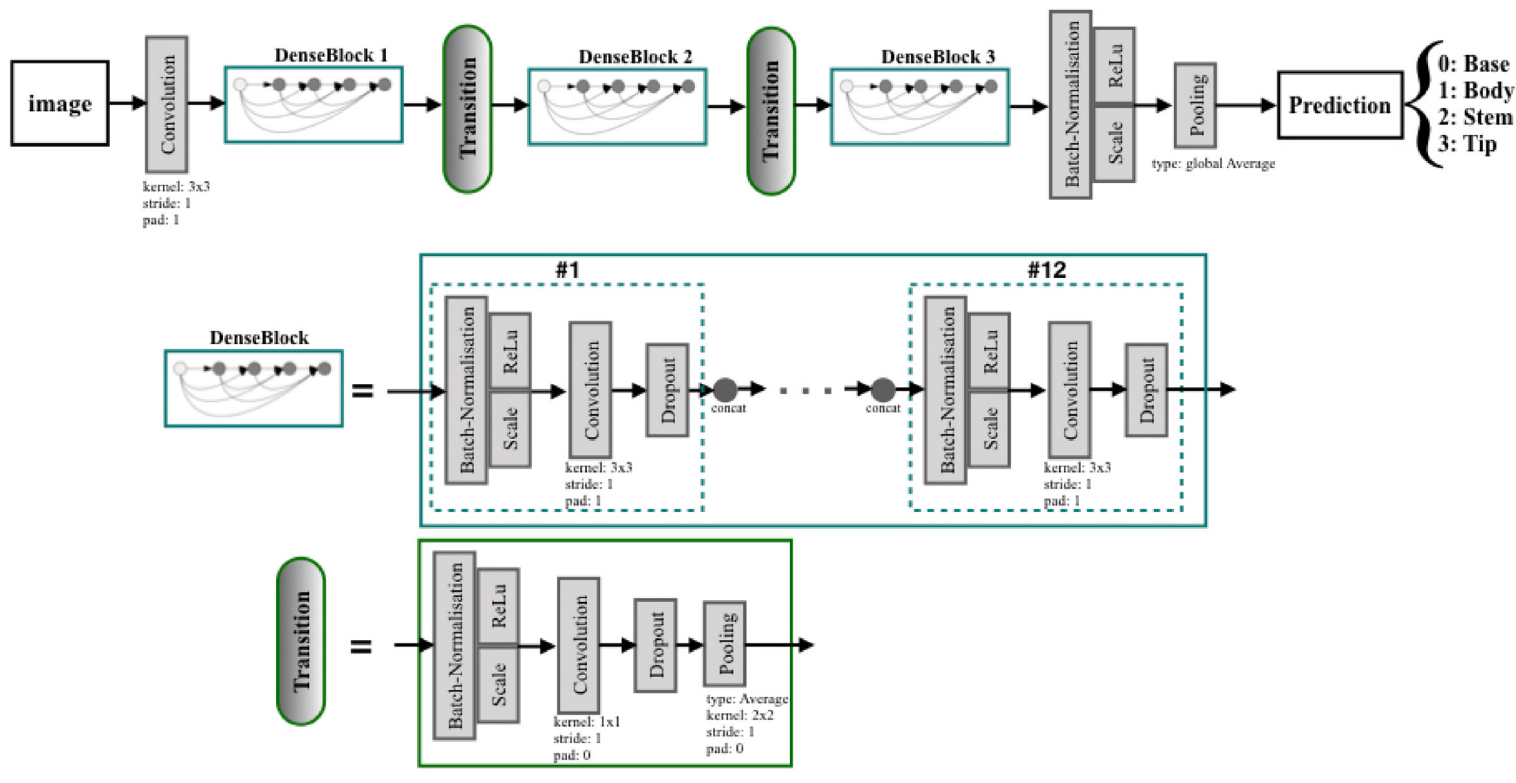
The DenseNet-Basic architecture used for patch-based *Arabidopsis* structural part classification. The feature map sizes in the 3 dense blocks were 32 × 32, 16 × 16, and 8 × 8, respectively.

DenseNet is a model notable for its key characteristic of bypassing signals from preceding layers to subsequent layers that enforces optimal information flow in the form of feature maps. Amongst DenseNet variants [44], DenseNet-Basic is a successful model proposed for the CIFAR10 [34] image classification challenge. Hereafter, DenseNet-Basic is referred to as “DenseNet.” A simple DenseNet is made up of a total of *L* layers, while each layer is responsible for implementing a specific non-linear transformation, which is a composite function of different operations such as batch normalization, rectified linear unit, pooling, and convolution [42, 44]. Within a dense block that consists of multiple densely connected layers with such composite functions, all layers are directly connected to each other, and each layer receives inputs (i.e., feature maps) from all preceding layers (as illustrated in the middle row of Fig. 6). The number of feature maps generated from the composite function layer is usually fixed and is called the growth rate (*k*) for the DenseNet.

To facilitate down-sampling for CNNs, the network used for our experiment consisted of multiple dense blocks. These dense blocks were connected to each other through transition layers (composed of a batch normalization layer, a 1 × 1 convolutional layer, a dropout layer, and a 2 × 2 average pooling layer as shown in the bottom row of Fig. 6).

The growth rate (*k*) was set to 12 for all dense blocks in order to generate narrow layers within the overall DenseNet structure (i.e., 3 dense blocks with equal number of layers and 2 transition layers). A relatively small growth rate (of 12) was found to be sufficient to obtain satisfying results on our target datasets. The initial convolution layer incorporated 16 convolutions of size 3 × 3 on the input images. The number of feature maps in all other layers follows the setting for *k*.

At the end of the last dense block (third dense block), a global average pooling was performed to minimize over-fitting by reducing the total number of parameters in the model. The final Softmax classifier of 4 output nodes will predict the probability for each class on the basis of the extracted features in the network. The rest of the model’s parameters with regards to the kernel, stride, and padding sizes were kept as default as detailed in Huang et al. [44].

#### Training

In our experiments with LeNet and DenseNet, a configuration similar to that in Huang et al. [44] has been applied. Both models were trained via a stochastic gradient descent solver with the parameters set to Gamma = 0.1 (for the learning rate decreasing factor), momentum = 0.9 (for weight update from the previous iteration), and weight-decay factor = 10^−5^. We trained LeNet and DenseNet with mini-batches of size 64 and 8 (according to our hardware specifications), respectively. Both models were trained using an initial learning rate of 0.001 with 33% step-down policy. LeNet was trained for 15 epochs, and DenseNet, for 30 epochs. In our implementation, the LeNet and DenseNet models pretrained on the CIFAR10 dataset [34] were used to initialize the weights, whilst the networks were fine-tuned using prepared training data from the silique dataset. In the pre-processing step for each model, the mean patch calculated on the training set patches was subtracted for each sample patch being fed.

All CNN training and testing was performed within the Caffe framework [45]. The computations were carried out using an NVIDIA GeForce GTX 1080 GPU, Intel Core i7-4790 processor, and Ubuntu 16.04 operating system.

Table 3 shows the classification accuracy and loss for both networks on the validation data from Set-1 after training.

**Table 3.** Classification results on the validation samples

	LeNet	DenseNet
Accuracy	80.55%	86.80%
Loss	0.64	0.37

#### Performance on patch-based classification

In the initial evaluation, we used the test samples in our model development data Set-1 to evaluate the classification and detection performance of both the shallow and deep networks. The aim of this comparative evaluation was to choose the best model for correct classification of patches and estimating silique counts on the smaller development dataset.

The classification results of both networks are presented in Tables 4 and 5 in terms of a confusion matrix, per-class precision and recall, and total classification precision and recall. Note that only annotated patches have been considered for this evaluation. The DenseNet network has higher representational power due to its deeper architecture and its use of features of multiple levels for classification in comparison to the LeNet network; its efficacy in the learning task has also been evidenced by its higher accuracy in classifying plant parts (as shown in Tables 4 and 5).

**Table 4.** Performance of patch-based classification on the testing images for model development using LeNet network

Predicted	Actual				Precision (%)	Recall (%)
	Base	Body	Stem	Tip		
Base	344	12	52	4	74.1	83.5
Body	15	280	26	30	79.5	79.8
Stem	14	29	270	4	75.8	85.2
Tip	91	31	8	169	81.6	56.5

Total precision = 77.8%; total recall = 76.2%.

**Table 5.** Performance of patch-based classification on the testing images for model development using DenseNet network

Predicted	Actual				Precision (%)	Recall (%)
	Base	Body	Stem	Tip		
Base	392	4	14	2	93.6	95.1
Body	15	290	13	33	93.2	82.6
Stem	11	14	290	2	91.5	91.5
Tip	1	3	0	295	88.9	98.7

Total precision = 91.8%; total recall = 92%.

## Post-processing for Silique Localization and Counting

### Image reconstruction

Given the classification of various patches in an image, post-processing can be applied to reconstruct the image and detect probable silique appearances. The plant regions are first identified from the background (including borders) using simple thresholding methods. Then the plant regions are further segmented into 4 classes based on labelling of the patches of interest.

**Figure 7:**
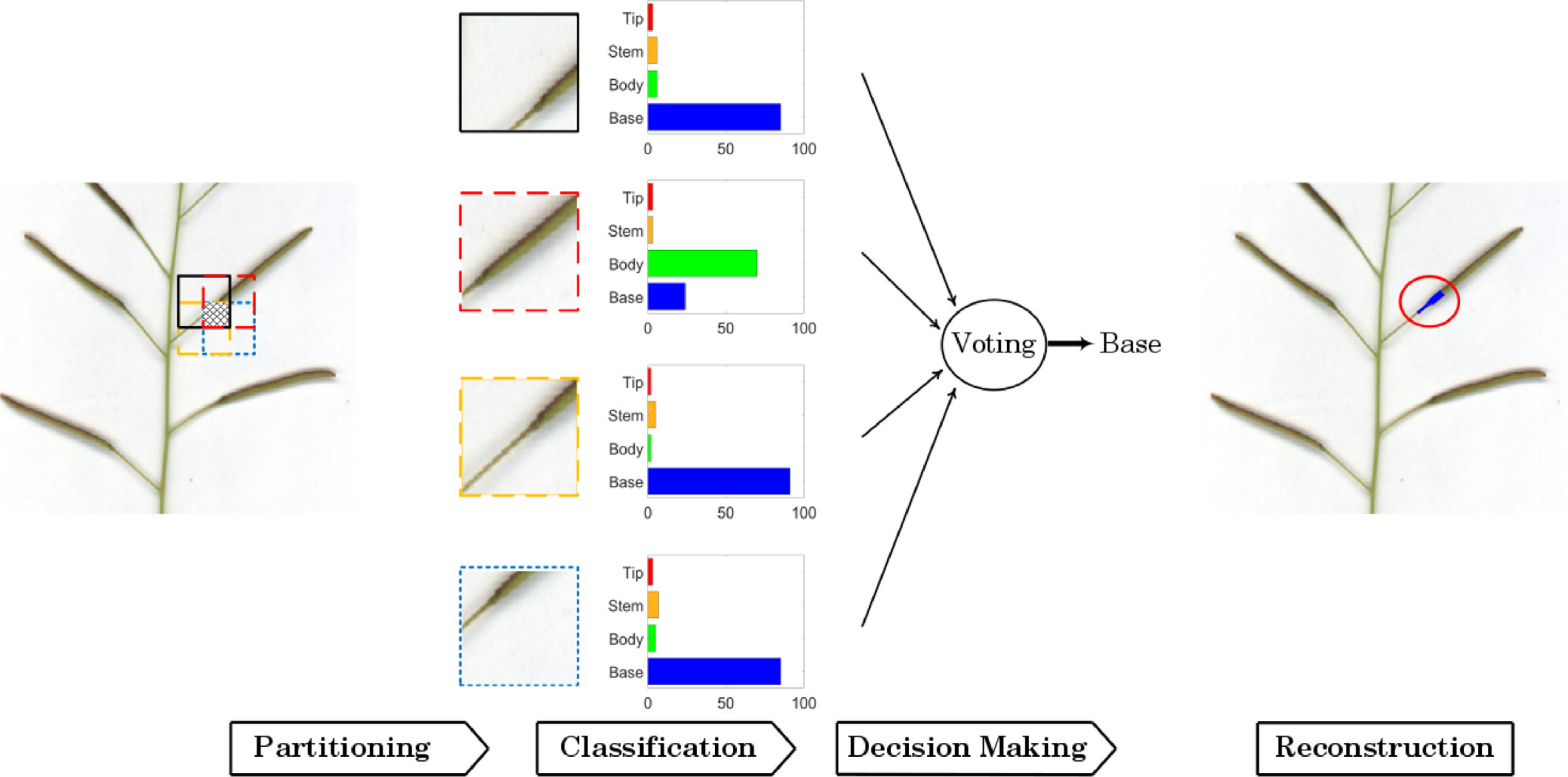
Flow chart of the sub-patch labelling step for image reconstruction.

Because the patches for a test image are generated with $50\%$ overlap along both the horizontal and vertical directions, each patch consists of 4 squares of equal size (16 × 16), called sub-patches. Each sub-patch has 4 class predictions from 4 adjacent patches, the final decision is inferred through majority vote, and the label for each pixel in the sub-patch was determined accordingly (see Fig. 7). In case of a tied vote for several classes, the average probability of those classes for the image will be assigned to the sub-patch and its pixels.

### Silique counting

To count siliques in the reconstructed image, a silique is defined as an area composed of 3 interconnected parts: 1 tip, 1 body, and 1 base in such a way that the body is located between the tip and the base (Fig. 1). In the areas where tips and bodies presenting shared borders were initially identified, these tip-body areas were extended through shared borders to search for the connected tips, which eventually established a combined area for a silique object.

In practice, many touching or overlapping siliques were observed in the captured images, which was a problem for detecting individual siliques accurately. In the case where 1 silique object area contained multiple tips or bases, the angle between the potentially overlaid siliques was calculated, using a cross-product between the different vectors linking the bases to the corresponding tips. For example, for the case of 2 siliques overlaying (often with the same apparent base or tip), the centres of tips and bases were computed; then using a cross-product, the centres were connected in order to calculate the angle between overlaid siliques. If the measured angle was larger than a predetermined threshold, the region was considered as 2 distinctive siliques, otherwise as a single silique. The value of the threshold was set to 0.05 rad in our experiments according to the resolution of the images. See supplementary Figure S1 for an illustrative example on how to detect/count individual siliques with overlapping regions.

## Test Results for Silique Counting

### Results on the test data for model development

**Figure 8:**
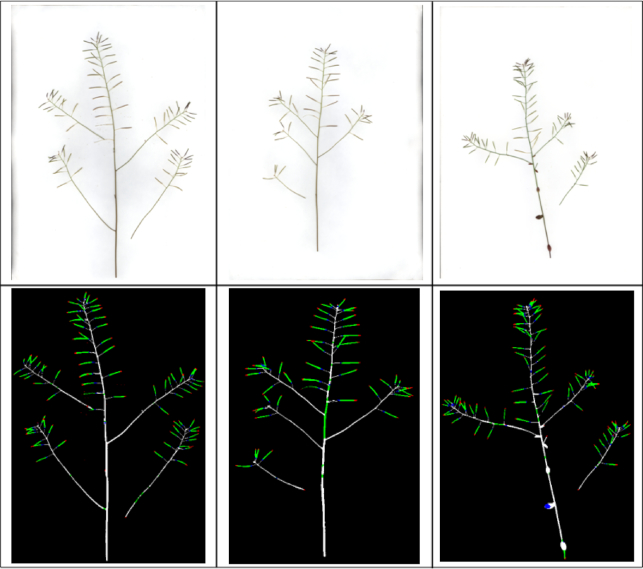
Three example results of labelling on the reconstructed plant images based the DenseNet patch-based classification. Tips, bodies, bases, and stems are indicated in red, green, blue, and white, respectively.

Fig. 8 shows the results of image reconstruction for several randomly selected images after patch classification (using the DenseNet network), with colours indicating different structural parts of the plant.

**Figure 9:**
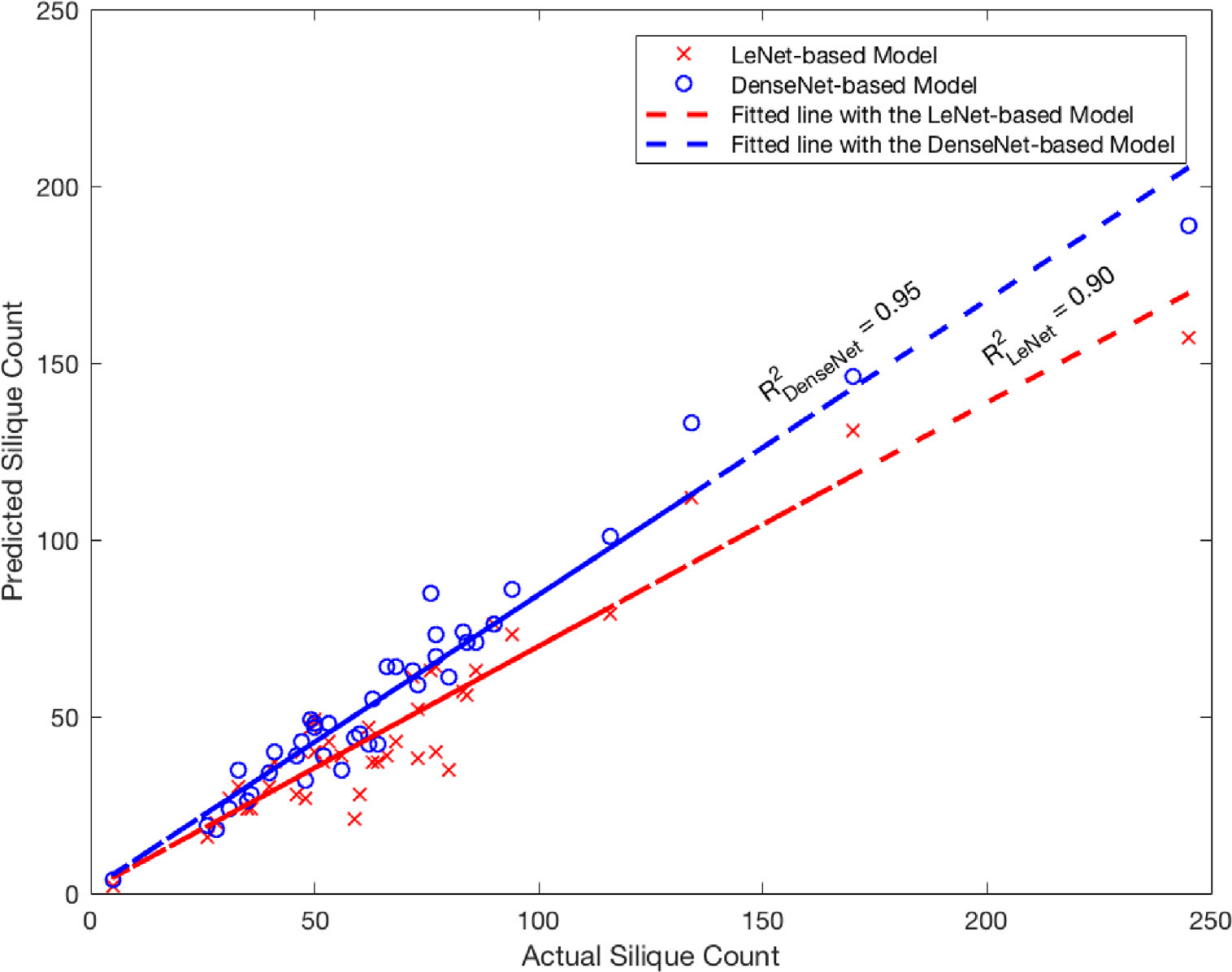
Predicted counts using the 2 models using validation and testing samples. *R*^2^ = 0.90 for the LeNet-based model and *R*^2^ = 0.95 for the DenseNet-based model.

**Figure 10:**
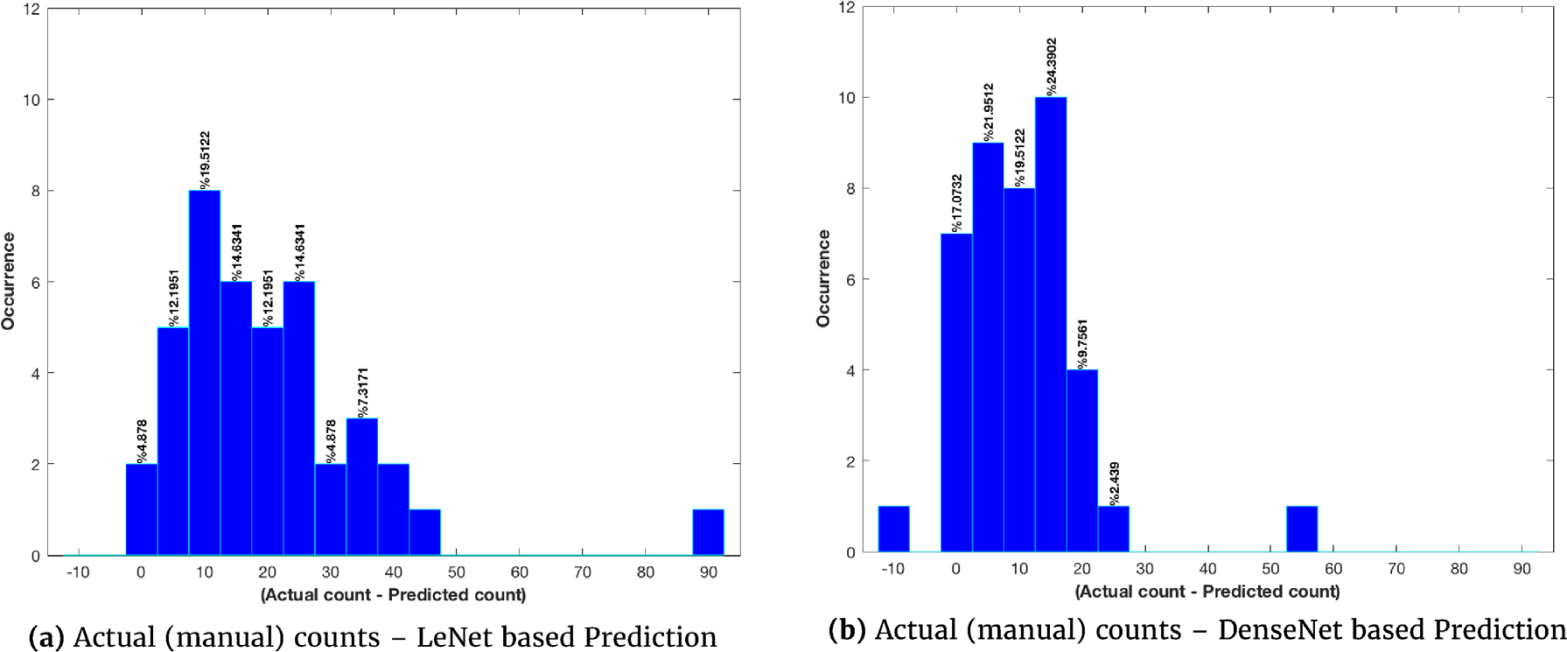
The histograms of errors in silique count prediction for the LeNet and DenseNet models.

Table 6 reports the performance of silique count prediction using the 2 different trained networks. In this table, the correlation coefficient (for the linear relationship between the prediction and the manual counts) shows that the prediction using the deeper model (DenseNet) is more accurate than that using the shallower model (LeNet). This linear correlation can be better seen in Fig. 9, which shows the scatter plots of the actual vs automated silique counts. We also examined the distribution of the errors (actual − prediction) in silique counting; see Fig. 10 for the histograms of errors for the 2 trained models. It appears that both LeNet and DenseNet underestimated the counts compared to manual counting in most cases.

**Table 6.** Performance for silique count prediction compared to manual counting on the 22 test images for model development

Metric	LeNet	DenseNet
Correlation coefficient	0.932	0.954
Root mean squared error	20.35	12.45

Comparing a shallow and a deep network for classifying image patches, we concluded that the classification results and the quality of the count estimation show improvement from using the deeper architecture. Therefore, DenseNet has been selected for identifying siliques because it seemed to be more robust to the variations in shape and size. This is probably in part a consequence of using a training set of images from diverse individuals harvested at different stages of silique maturation.

### Results on the separate test data

**Figure 11:**
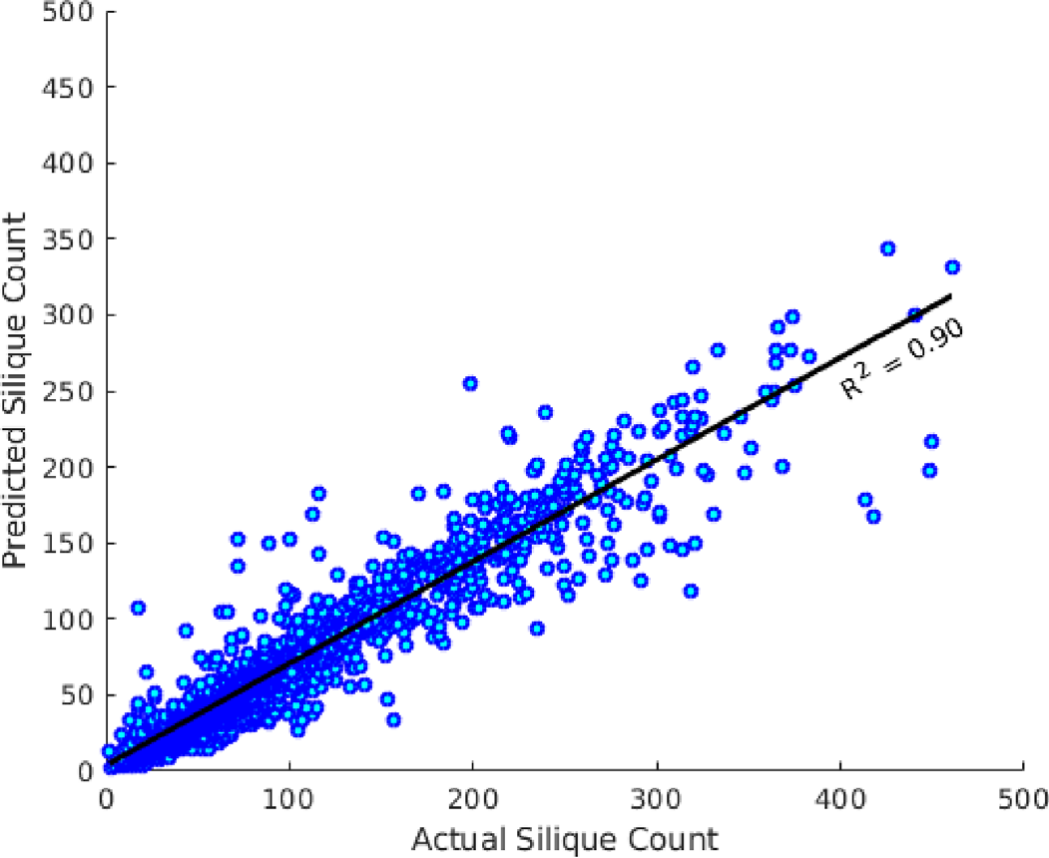
Predicted silique count and manual counting from Set-2 testing samples including 2,408 images. *R*^2^ = 0.90.

To further evaluate the proposed framework, we used a separate large dataset of 2,408 images available within the NPPC. The scatter plot in Fig. 11 shows a high positive correlation (Pearson correlation coefficient *R*^2^ = 0.90) between the manual counts and automated counts. With the reconstructed silique objects, additional morphological features could be extracted including silique length. Predicted silique number and statistics for silique length (mean, maximum, and minimum) per image are reported in Supplementary Data S1. Preliminary validation of the mean length estimate has been given in Supplementary Figure S2 and Supplementary Data S2 and S3.

**Figure 12:**
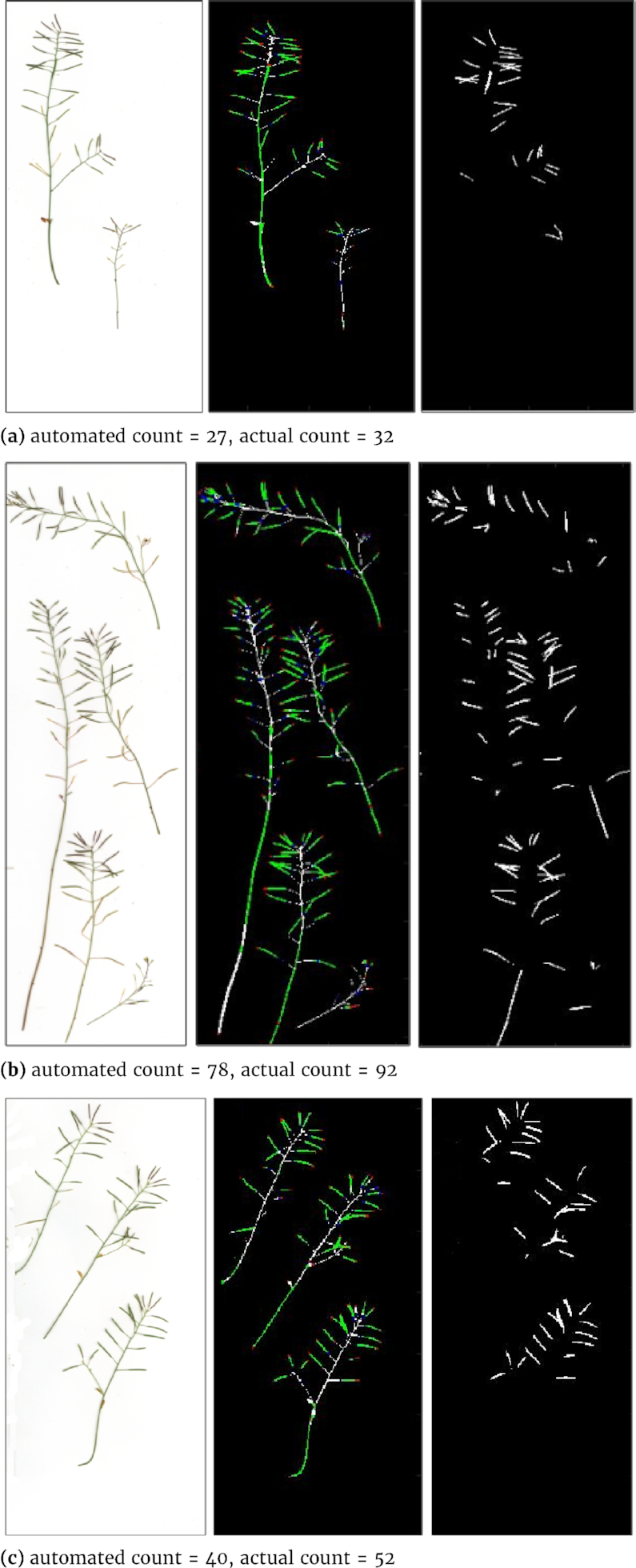
Results of the DenseNet framework applied to some random samples from the larger testing dataset. *From left to right*: original plant images, sub-patch labelling and image reconstruction (Tips, bodies, bases, and stems are indicated in red, green, blue, and white, respectively), and silique region detection (in white).

The CNN-based prediction tends to underestimate compared to actual manual counting. To better understand where the problem lies, detailed detection results have also been visualized; see Fig. 12 for some random examples. It seems that the current-post processing method might have difficulty in detecting some small or overlapping siliques.

## Discussion

A recent computer vision approach to fruit number estimation involves linear regression using selected skeleton descriptors (such as junction numbers and number of triple points) extracted after segmentation and 2D skeletonization, resulting in a validation correlation of *R*^2^ = 0.91 between observed and automated values for the best-performing model on the 100 examples [2] in a development dataset. When applied on the dataset from a separate experiment, although the model can qualitatively capture the main phenotype under investigation, its accuracy against the manual counts decreased significantly to *R*^2^ of 0.7. This suggests that this regression approach to fruit counting might not be generalized to other conditions as effectively as our object recognition approach. Apparently this non-deep learning approach used only “handcrafted” global features, with resulting models more specific to the conditions for training, whereas our approach used both local features (for patch classification) and some more global features (for object reconstruction).

On the basis of our test results on silique counting, we expect our method to be useful for species with similar fruit morphology such as canola (oilseed rape) and other brassicas. However, the CNN will most likely need to be fine-tuned for diverse silique morphology and imaging conditions.

There are several promising directions for future work for which the developed software can be improved such as the detection of other traits like silique length or branch number. These 2 traits have been reported to be a good proxy of seed number and therefore could be important for estimating productivity [46]. The following considerations should be taken into account in future to improve the classification and detection performance:

- The robustness of the representations in both networks relied largely on the quality and quantity of the training and test data. Increased variety in the training samples (along with artificial augmentation) should provide more robust learned representations and may facilitate extension to other species.
- Deep learning models can take the whole image or the patches as input. In this study, a patch-based classifier was used and the image was scanned over with a sliding window, classifying the patches. However, feeding all patches to the network was time-consuming and the designated patch overlap produces substantial redundancy. To overcome these issues, deep neural networks taking the whole image as input for object detection can be explored.
- Generative adversarial networks [47] have been widely used in segmentation problems on real-world [48, 49] and medical data (see our recent application of these models on medical images [50, 51]). To avoid the need for post-processing (which affects the performance), different types of generative adversarial networks should be investigated.
- DeepPod can be used to accelerate the development of even more robust fruit recognition approaches. DeepPod can rapidly provide more annotated images because the output of the proposed DenseNet model can be used to automatically generate detailed fruit annotation suggestions. A human annotator would then focus on correcting false-negative results (by adding missed siliques) and false-positive results (or removing falsely detected ones) instead of spending so much time on marking each fruit contour individually.

## Conclusion

In summary, we have developed DeepPod, an image-based deep learning framework for fruit counting. We have demonstrated DeepPod’s effectiveness in silique detection and counting for *Arabidopsis*, as well as challenges due to presence of overlapping siliques and variability in fruit morphology. The pipeline developed has been shown to be cost-effective in image annotation for model development. To further improve the pipeline, more robust and scale-invariant methods will be investigated for object detection and for extraction of more morphological traits. In addition, active learning and transfer learning could be applied for more effective data annotation and machine learning modelling.

## Availability of Source Code and Requirements

- Project name: DeepPod
- Project home page: https://github.com/AzmHmd/DeepPod.git
- Operating system(s): Platform independent
- Programming language: MATLAB
- Other requirements: CUDA version 8.0, CuDNN version v5.1, BLAS: atlas, Caffe version 1.0.0-rc3, DIGITS version 5.1-dev, MATLAB version 9.3 or above
- License: MIT
- The annotation toolbox (also included in DeepPod project) has been registered in SciCrunch as RRID:SCR_017413

## Availability of Supporting Data and Materials

The dataset for model development (Set-1, including 144 raw images and their annotations, and manual silique counts) and the dataset for testing (Set-2, including 2,408 raw images and their manual silique counts) are available in the Aberystwyth research data repository [53]. Snapshots of our code and other supporting data are available in the *GigaScience* repository, GigaDB [52].

## Additional Files

**Supplementary Figure S1:** An illustrative example on identification of individual siliques with overlapping regions

**Supplementary Figure S2:** Comparison of predicted mean silique length with manual estimate

**Supplementary Data S1:** CSV file reporting the predicted silique count, mean, and range of silique length (in pixels) for each image in Set-2

**Supplementary Data S2:** CSV file reporting the manual measurement of individual silique length (2,359 siliques out of the 32 images randomly selected from Set-2)

**Supplementary Data S3:** CSV file for the data used to evaluate the predicted mean silique length with the manual measure for the 32 annotated images in Set-2

giaa012_GIGA-D-19-00251_Original_Submission

giaa012_GIGA-D-19-00251_Revision_1

giaa012_GIGA-D-19-00251_Revision_2

giaa012_GIGA-D-19-00251_Revision_3

giaa012_Response_to_Reviewer_Comments_Original_Submission

giaa012_Response_to_Reviewer_Comments_Revision_1

giaa012_Response_to_Reviewer_Comments_Revision_2

giaa012_Reviewer_1_Report_Original_Submission Chris Armit -- 7/31/2019 Reviewed

giaa012_Reviewer_1_Report_Revision_1 Chris Armit -- 11/26/2019 Reviewed

giaa012_Reviewer_2_Report_Original_Submission Dijun Chen -- 8/8/2019 Reviewed

giaa012_Reviewer_2_Report_Revision_1 Dijun Chen -- 11/26/2019 Reviewed

giaa012_Reviewer_3_Report_Original_Submission Andrew French -- 8/21/2019 Reviewed

giaa012_Reviewer_3_Report_Revision_1 Andrew French -- 12/6/2019 Reviewed

giaa012_Supplemental_Files

## Abbreviations

Caffe: Convolutional Architecture for Fast Feature Embedding; CNN: convolutional neural network; GUI: graphical user interface; LSTM: long short-term memory; NPPC: National Plant Phenomic Centre; RCNN: regional CNN; SSD: Single shot multibox detector; YOLO: you only look once.

## Competing Interests

The authors declare that they have no competing interests.

## Authors' Contributions

J.H.D., F.C., and C.L. designed the study and provided the images. G.G.-M. performed manual counting and manual annotations. A.H. developed the annotation toolbox and performed the deep learning and data analysis, testing, and evaluation tasks. A.H. and M.G. carried out the post-processing analysis. A.H. and G.G.-M. drafted the manuscript. All the authors provided comments and corrected the manuscript.

## Acknowledgements

The authors gratefully acknowledge Sandy Spence and Alun Jones for their support and maintenance of the GPU and the systems used for this research. We acknowledge Dr Jay Biernaskie from University of Oxford for allowing us to use a previously unpublished image dataset (Set-1 and Set-2). We also thank the team of the National Plant Phenomics Centre, mainly Lina Avila Clasen for her help in acquiring the images and manual counting; Jason Brook for running the PSI small plant platform, and Karen Askew for assisting with plant care. G.G.-M. acknowledges receipt of a AberDoc scholarship from Aberystwyth University; J.H.D., F.C., and C.L. funding from BBSRC (grants BB/CAP1730/1, BB/P013376/1, and BB/P003095/1; and J.H.D. acknowledge support from the National Science Foundation (cROP project: 1340112).
