# Supplementary material for: DeepPod: a convolutional neural network based quantification of fruit number in Arabidopsis
Source: Gigascience. 2020 Mar 4;9(3):giaa012. doi: 10.1093/gigascience/giaa012 (PMC7055469; doi:10.1093/gigascience/giaa012)
Supplement: giaa012_GIGA-D-19-00251_Original_Submission [file giaa012_giga-d-19-00251_original_submission.pdf]

GigaScience

DeepPod: A Convolutional Neural Network Based Quantification of Fruit Number in Arabidopsis

--Manuscript Draft--

**Manuscript Number:**	GIGA-D-19-00251
**Full Title:**	DeepPod: A Convolutional Neural Network Based Quantification of Fruit Number in Arabidopsis
**Article Type:**	Technical Note
**Funding Information:**	Aberystwyth University (AberDoc Scholarship) — Ms Azam Hamidinekoo; Ms Gina A. Garzón-Martínez; Mr Morteza Ghahremani<br>Biotechnology and Biological Sciences Research Council (BB/CAP1730/1) — Prof John H. Doonan<br>Biotechnology and Biological Sciences Research Council (BB/P013376/1) — Prof John H. Doonan<br>Biotechnology and Biological Sciences Research Council (BB/P003095/1) — Prof John H. Doonan
**Abstract:**	High-throughput phenotyping based on non-destructive imaging has great potential in plant biology and breeding programs. However, efficient feature extraction and quantification from image data remains a bottleneck that needs to be addressed. Advances in sensor technology have led to the increasing use of imaging to monitor and measure a range of plants including the model Arabidopsis thaliana. These extensive datasets contain diverse trait information but feature extraction is often still implemented using approaches requiring substantial manual input. The computational detection and segmentation of individual fruits from images is a challenging task, for which we have developed DeepPod, a patch based two-phase deep learning framework. The associated manual annotation task is simple and cost effective without the need for detailed segmentation or bounding boxes. Convolutional neural networks (CNNs) are used for classifying different parts of the plant inflorescence, including the tip, base and body of the siliques and the stem inflorescence. In a post processing step, different parts of the same silique are joined together for silique detection and localisation, whilst taking into account possible overlapping among the siliques. The proposed framework is further validated on a separate test dataset of 2,408 images. Comparisons of the CNN based prediction with manual counting (R2 = 0.90) showed the desired capability of methods for estimating silique number.
**Corresponding Author:**	Chuan Lu<br>Aberystwyth University<br>Aberystwyth, Ceredigion UNITED KINGDOM
**Corresponding Author Secondary Information:**	
**Corresponding Author's Institution:**	Aberystwyth University
**Corresponding Author's Secondary Institution:**	
**First Author:**	Azam Hamidinekoo
**First Author Secondary Information:**	
**Order of Authors:**	Azam Hamidinekoo<br>Gina A. Garzón-Martínez<br>Morteza Ghahremani<br>Fiona M. K. Corke

Powered by Editorial Manager® and ProduXion Manager® from Aries Systems Corporation

	Reyer Zwiggelaar
	John H. Doonan
	Chuan Lu
**Order of Authors Secondary Information:**	
**Additional Information:**	
**Question**	**Response**
Are you submitting this manuscript to a special series or article collection?	No
**Experimental design and statistics**<br><br>Full details of the experimental design and statistical methods used should be given in the Methods section, as detailed in our Minimum Standards Reporting Checklist. Information essential to interpreting the data presented should be made available in the figure legends.<br><br>Have you included all the information requested in your manuscript?	Yes
**Resources**<br><br>A description of all resources used, including antibodies, cell lines, animals and software tools, with enough information to allow them to be uniquely identified, should be included in the Methods section. Authors are strongly encouraged to cite Research Resource Identifiers (RRIDs) for antibodies, model organisms and tools, where possible.<br><br>Have you included the information requested as detailed in our Minimum Standards Reporting Checklist?	Yes
**Availability of data and materials**<br><br>All datasets and code on which the conclusions of the paper rely must be either included in your submission or deposited in publicly available repositories (where available and ethically	Yes

appropriate), referencing such data using a unique identifier in the references and in the “Availability of Data and Materials” section of your manuscript.<br><br>Have you have met the above requirement as detailed in our Minimum Standards Reporting Checklist?	

manuscript
Click here to download Manuscript deeppod_manuscript.pdf

GigaScience, 2019, 1–12

doi: xx.xxxx/xxxx
Manuscript in Preparation
Paper

PAPER

DeepPod: A Convolutional Neural Network Based Quantification of Fruit Number in *Arabidopsis*

Azam Hamidinekoo[1,*,†], Gina A. Garzón-Martínez[2,*,†], Morteza Ghahremani[1], Fiona M. K. Corke[2], Reyer Zwiggelaar[1], John H. Doonan[2] and Chuan Lu[1, ‡]

[1]Department of Computer Science, Aberystwyth University, Aberystwyth, United Kingdom and [2]National Plant Phenomics Centre, Institute of Biological, Environmental and Rural Sciences, Aberystwyth University, Aberystwyth, United Kingdom

[*]azh2@aber.ac.uk; gig7@aber.ac.uk; cul@aber.ac.uk
[†]Contributed equally.
[‡]Corresponding author.

Abstract

High-throughput phenotyping based on non-destructive imaging has great potential in plant biology and breeding programs. However, efficient feature extraction and quantification from image data remains a bottleneck that needs to be addressed. Advances in sensor technology have led to the increasing use of imaging to monitor and measure a range of plants including the model *Arabidopsis thaliana*. These extensive datasets contain diverse trait information but feature extraction is often still implemented using approaches requiring substantial manual input. The computational detection and segmentation of individual fruits from images is a challenging task, for which we have developed DeepPod, a patch based two-phase deep learning framework. The associated manual annotation task is simple and cost effective without the need for detailed segmentation or bounding boxes. Convolutional neural networks (CNNs) are used for classifying different parts of the plant inflorescence, including the tip, base and body of the siliques and the stem inflorescence. In a post processing step, different parts of the same silique are joined together for silique detection and localisation, whilst taking into account possible overlapping among the siliques. The proposed framework is further validated on a separate test dataset of 2,408 images. Comparisons of the CNN based prediction with manual counting (R^2 = 0.90) showed the desired capability of methods for estimating silique number.

Key words: Plant phenotyping; image analysis; deep learning; object detection, fruit counting; *Arabidopsis*

Introduction

Photometrics (imaging following by computationally assisted feature extraction and measurement) promises to revolutionise biological research and agricultural production systems [1, 2, 3, 4, 5]. Automation of workflows remains a key challenge in the scaling of these approaches to cope with the requirements of large genetic experiments or, indeed, food production systems. Phenotyping aims to measure observable plant features, often as a response of environmental cues and/or variability between individuals. Traditionally, phenotyping has been a labour-intensive and costly process, usually manual and often destructive. High-throughput phenotyping technologies aim to address this problem by the use of non-destructive approaches either in glasshouses [1, 6, 2] or directly in the field [4, 7] integrating imaging, robotics, spectroscopy, high tech sensors and high-performance computing [8, 3].

Imaging has the potential to generate an enormous volume of data in real time, while image analysis to extract useful information is currently the main bottleneck. The ex-

Compiled on: July 10, 2019.
Draft manuscript prepared by the author.

traction of quantitative traits relies on the development and use of improved software techniques. Machine learning tools have been used to identify patterns in large biological datasets [8, 9, 10, 11, 12]. Recently, deep learning tools have been applied to accurately extract features from plant images [13, 14, 15, 16, 17, 18, 19, 20, 21].

Model organisms have been widely used to dissect different biological processes and provide a useful means to test and develop new methods that can subsequently be more widely applied to crop and ecological scenarios. *Arabidopsis thaliana* is a small, flowering plant widely used to address questions related to plant genetics, molecular, evolution, ecology, physiology, among others [22, 23, 24]. The seedling produces a small rosette that increases in size by addition of leaves. The central meristem produces an inflorescence that produces flowers and then fruits. The fruits are also known as pods or siliques [24]. The measurement of traits, such as growth rate, flowering and fruit number are key to evaluate plant performance and reproductive fitness [25]. However, many high-throughput imaging studies focus on growth dynamics of the rosette [26, 27, 9, 28], despite the importance of fruit production in reproductive and evolutionary processes [2, 29, 30, 31].

This work demonstrates that deep learning can be used to estimate fruit number from images. In particular, we have developed DeepPod, a framework for *Arabidopsis* silique detection that involves a deep neural network for patch based classification and an object reconstructor for silique localisation and counting. The framework has been validated using a separate dataset of 2408 images from biological experiments. This allowed the analysis of large numbers of plants inflorescences in an accurate and effective way providing a cost effective alternative to manual counting.

Background

Convolutional Neural Networks (CNNs) have become the dominant type of models for image classification [32]. The input for a CNN, typically an image, can be represented as a three-dimensional array of height×width×channels. A CNN contains convolutional layers, where inputs are passed through various filters for extracting features that are arranged as feature maps, prior to using the fully connected layers for classification or regression. The weights or parameters of the filters are shared among the neurons of the convolutional layers [33], not only to encourage detection of repeated patterns in the image but also to reduce the number of parameters for the network to learn. Other types of layers such as pooling are also often used in combination with convolutional layers to reduce the dimensionality of feature maps. A CNN can be trained using a back-propagation algorithm to update the weights in an iterative process, in order to minimise the loss function that measures the discrepancy between the predicted output and actual output for the training examples. What makes CNNs particularly attractive in computer vision is that they can directly extract features from images without the need for time-consuming, hand-crafted pre-processing or feature extraction steps, unlike classical machine learning approaches [34].

Recent publications have reported the application of deep learning in various plant phenotyping tasks such as leaf counting, age estimation, mutant classification, disease detection, fruit classification and plant organ localisation [14, 20, 19, 21, 18, 16, 15, 13]. Mohanty *et al.* [14] trained deep convolutional neural networks to identify 14 crop species and 26 diseases using a publicly available plant disease dataset. They built models with architectures of AlexNet [35] and GoogleNet [36] using transfer learning. Wang *et al.* [20] employed CNNs to establish disease severity in apple black rot images. Deep learning meta-architectures have also been considered for more complex scenarios. Fuentes *et al.* [19] demonstrated a combination of CNNs and deep feature extractors to recognise different diseases and pests in tomatoes, which dealt with inter- and intra-class variations. Deep learning was also used for cassava disease detection via mobile devices [21]. Pawara *et al.* [18] applied CNNs to classify leaf, fruits and flowers in field images. They compared the performance of classical classifiers to CNNs, where architectures such as GoogleNet and AlexNet gave the best results in the plant-related datasets used. Taghavi *et al.* [16] proposed a CNN-LSTM (-Long Short Term Memory) framework for plant classification using temporal sequence of images. Particularly the model features were learned using CNNs and the plant growth variation over time were modeled with LSTMs. Ubbens *et al.* [15] used CNNs for regression to perform leaf counting. They used rendered images of synthetic plants to augment *Arabidopsis* rosette dataset and concluded that the augmentation with high-quality 3D synthetic plants improved the performance of leaf counting while real and synthetic plants could be interchangeably used for training a neural network. Pound *et al.* [13] demonstrated wheat root and shoot feature identification and localisation using a shallow CNN architecture for patch classification. The model had two convolutional layers followed by max pooling layers. They found that the leaf tips represented the hardest classification problem compared to the leaf base due to the existing variations in orientation, size, shape and colour of tips in their dataset.

Our proposed framework treats the silique (or pod) counting problem as an object detection and segmentation problem followed by counting. One popular approach of deep learning frameworks for object detection is to train a single convolutional neural network to jointly perform object classification and localisation tasks, where the object localisations are usually defined by bounding boxes. Examples of such networks include Fast-RCNN (Regional-CNN), SSD (Single Shot Multibox Detector), YOLO (You Only Look Once) [37]. However training of such networks require labelled data with detailed segmentation or bounding boxes of individual objects, which are obtained usually through a very tedious manual process. Moreover, the image size allowed for the network input is limited due to the complexity of network architecture and the available memory.

In our case, the resolution of the raw images needs to be sufficiently high in order to preserve details of pods that are small and narrow, often overlapping. A single image can also contain a wide variation in the number of fruits from 0 to near 400, which poses further challenges for deep learning models when the available labelled data is limited.

To address these issues, we adopted an alternative approach that performs patch based classification and localisation in two separate phases. The first step is to classify a region of a suitable size in the original image into different parts of the inflorescences. In the localisation phase, each original image will be scanned and each patch classified as silique/not silique (i.e. as one of the four classes including the tip, base or body of siliques, and the stem inflorescence). Given an accurate classification of patches as silique/not silique, one could then estimate the number of siliques and their lengths to a good precision. The manual annotation task for the proposed framework was simple, involving collection of sufficient pixels from different defined structural parts of the plant.

Data Acquisition

A set of 2,552 images of mature inflorescences from different individuals and available within the National Plant Phenomics Centre (NPPC) (Aberystwtyh University, United King-

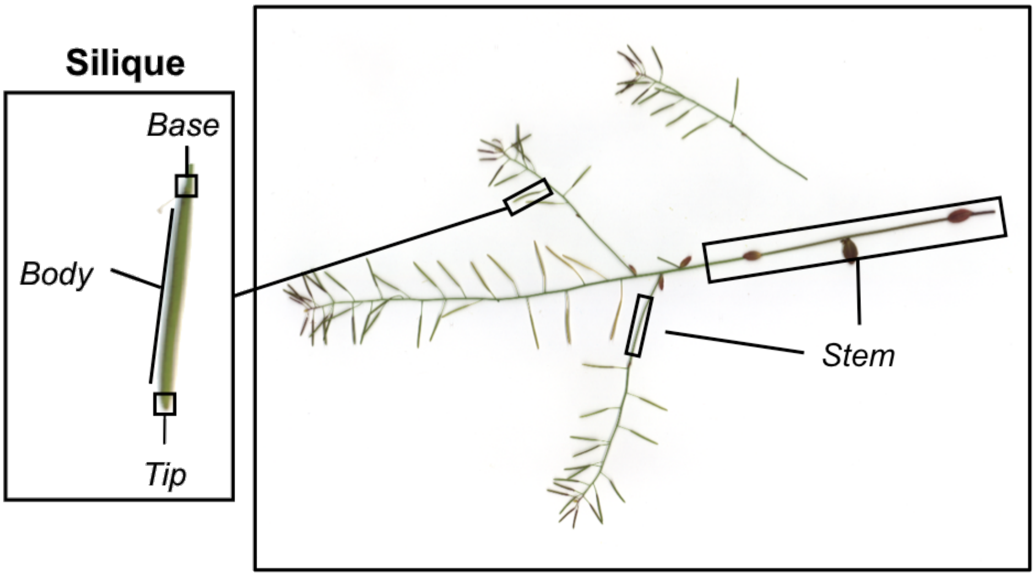

Figure 1. An illustrative example of image and features (the important parts of the plants) annotated for patch based classification..

dom), were used to establish and test the CNN pipeline. A subset of this dataset (referred to as Set-1 = 144 images) was randomly selected for manual annotation and then used to train one shallow and one deep convolutional neural network. A total of 2,408 images (referred to as Set-2) were used to test the performance of the selected model. The mature inflorescence or stem of each plant, with attached fruits, was imaged in a flatbed scanner (Plustek, OpticPro A320) at 300 dpi and stored in .PNG format with image size equal to 3600×5100. A sample image is shown in Figure 1. Manual counting of fruits from the images was done by a single person, minimizing operator variation.

Patch based Classification using CNNs

Data Preparation for Model Development

Data Annotation

An annotation tool with a graphical user interface (GUI) was built (in MatLab) to assist with manual annotation of different parts of the inflorescence. Figure 2 shows some screenshots of annotation via the GUI. The user selects the class type (tip, base, body of the silique and stem) and clicks on the respective parts on each input image. The annotated parts (points clicked) were saved as defined locations based on image coordinates. An example annotated image illustrating the predefined parts of the silique (tip, base, body and stem) is given in Figure 3. This tool was used to manually annotate Set-1, which was used to develop the patch classifiers (see Section Patch Based Classification Problem).

The main advantages of this annotation platform include its relatively low cost and ease of use. Compared to other annotation approaches that require detailed segmentation, polygons or bounding boxes, this approach requires annotation of just four main structural elements. Using this platform, Set-1 was manually annotated by a single person in a total of 36 working hours.

Table 1 shows the number of annotations performed per class (before augmentation). This dataset was used in the initial training step for classifying whole inflorescence into defined parts. In order to prepare patches for classification, Set-1 was randomly split into training, validation and test sets as rounded of 65%, 20% and 15% of the 144 images.

Table 1. Summary statistics for data annotation performed on Set-1.

Feature	Number of manual annotations
Silique Tip	7299
Silique Base	8058
Silique Body	11187
Stem	10266

Patch Generation & Augmentation

Using the annotated data to prepare training samples, square bounding box patches were extracted while being centred at the manually annotated points. Subsequently, data augmentation [38, 39] was performed to increase the amount of training data via specific transformations as well as considering frames different from the centred ones. The patches of size 50 × 50 were first extracted. Then, random 32 × 32 pixel crops followed by random mirroring or rotation were performed. For preprocessing, we normalised the data using the channel means and standard deviations on the training set. For validation samples, no augmentation was undertaken and the 32 × 32 patches centred at the annotated points were extracted. Figure 4 shows various examples of each class that were used in the training procedure.

Data Preparation for Testing

The training patches were centred at the annotated points followed by augmentation, as described earlier. To prepare test samples, the difference in the distribution between the testing data and the training/ validation data (that were used during training time) was taken into account. First, the whole image was scanned over with a sliding window and tiled into 32 × 32 patches with 50% overlap both in the vertical and horizontal direction (see Figure 7). Most pixels within the area of interest (plant area) would hence be included in four different patches. The patches belonging to the white background (lacking plant pixels) were excluded by thresholding.

The rationale behind selecting overlapping regions was (1) to increase the number of patches by a factor of four compared to without overlapping; (2) to make the patch classification more robust by combining multiple predictions and (3) to exclude ambiguous patch examples.

When applying the model to test data, the difference be-

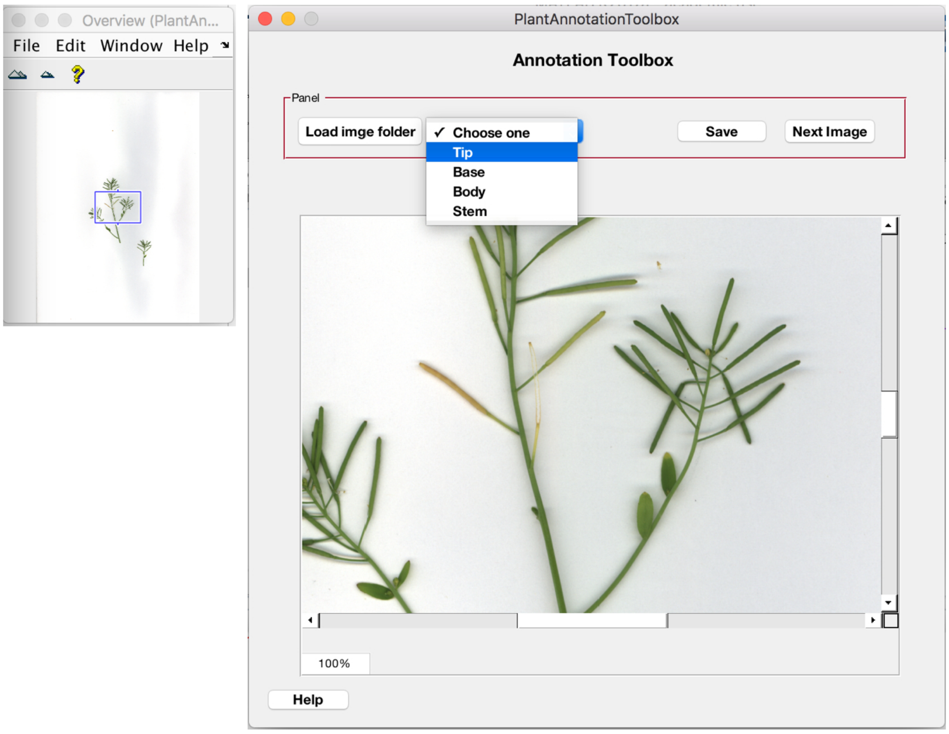

Figure 2. The developed GUI used for manually annotating plant parts.

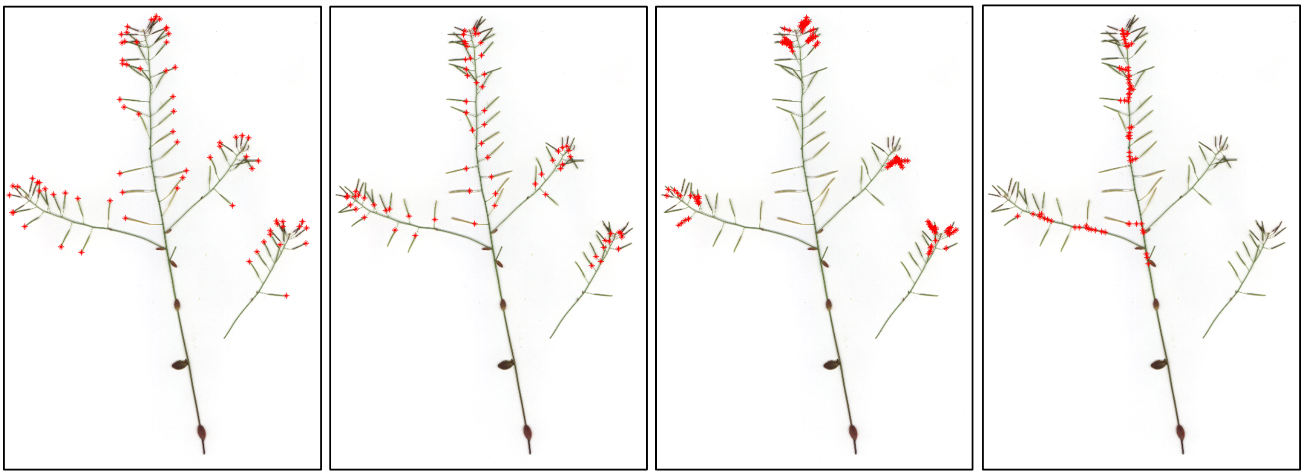

Figure 3. Example annotated images (from left to right) for tip, base, body and stem.

tween the sample distribution for training and that for testing, i.e. presence of potential covariate and dataset shift, can adversely affect the model generalisation performance. To address this issue, each test image will also be normalised using the channel wise mean and standard deviation of the training set.

Then the resultant patches were fed to the trained networks and the classification outcomes for each sample patch (tip, base, stem, body) were computed.

Building CNN Classifiers

In the next step, CNN-based classifiers were built to take extracted patches of interest as input, and to output probability scores for different labels $\{0, 1, 2, 3\}$ indicating the probability that the input patch contains a base, body, stem and tip, respectively.

Network Architecture

LeNet is a pioneering convolutional network that was proposed to classify handwriting digits [40]. LeNet architecture [41], is a set of three convolutional layers stacked on top of each other, followed by two fully connected layers and finally ending with a Softmax layer (see Figure 5).

DenseNet is a model notable for its key characteristic of bypassing signals from preceding layers to subsequent layers that enforces optimal information flow in the form of feature maps. Amongst DenseNet variants [42], DenseNet-Basic is a successful model proposed for the CIFAR10 [34] image classification challenge. Hereafter, DenseNet-Basic will be referred to as "DenseNet". A simple DenseNet is made up of a total of L layers, while each layer is responsible for implementing a specific non-

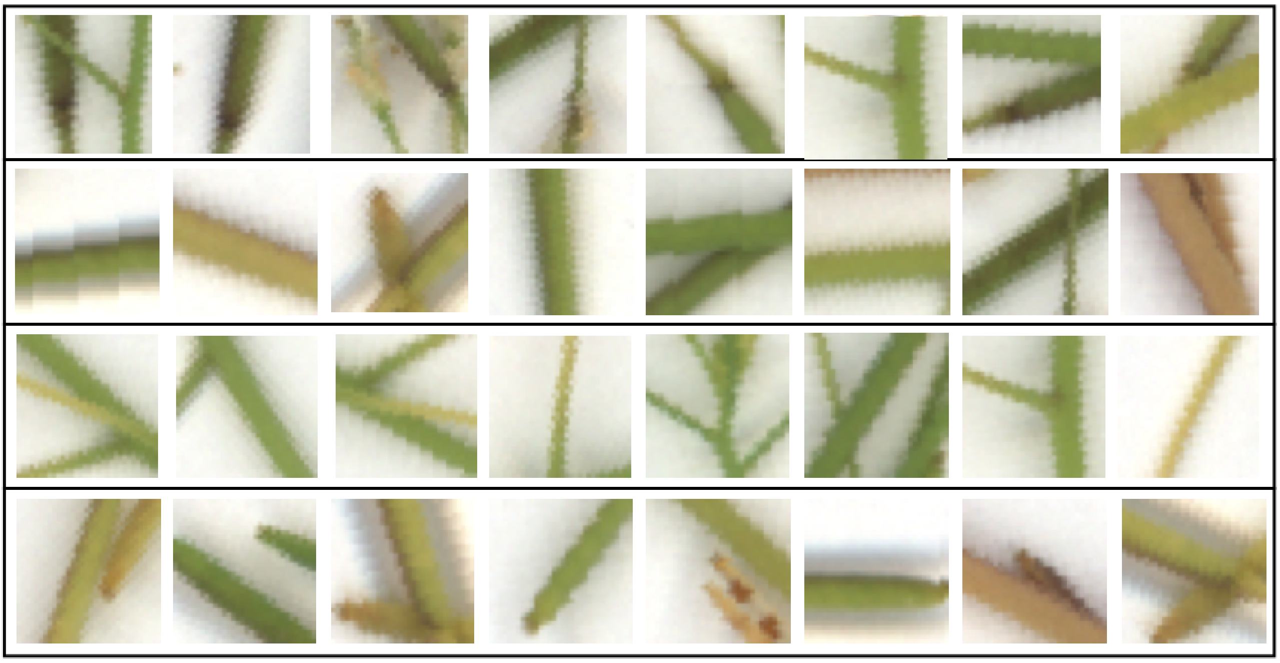

Figure 4. Example extracted patches using manual annotations. From top to bottom: samples of base, body, stem and tip, respectively. Note that, the key structural elements are not always centered in the patches due to the random cropping process for patch extraction.

linear transformation, which is a composite function of different operations such as Batch Normalisation, Rectified Linear Unit, Pooling and Convolution [40, 42]. Within a dense block that consists of multiple densely connected layers with such composite functions, all layers are directly connected to each other, and each layer receives inputs (i.e. feature maps) from all preceding layers (as illustrated in the middle row of Figure 6). The number of feature maps generated from the composite function layer is usually fixed and is called the growth rate (k) for the DenseNet.

To facilitate down-sampling for CNNs, the network used for our experiment consisted of multiple dense blocks. And the dense blocks were connected to each other through transition layers (composed of a batch normalisation layer, a 1×1 convolutional layer, dropout layer and a 2×2 average pooling layer as shown in the bottom row of Figure 6).

The growth rate (k) was set to 12 for all dense blocks in order to generate narrow layers within the overall DenseNet structure (i.e. 3 dense-blocks with equal number of layers and 2 transition layers). A relatively small growth rate (of 12) was found to be sufficient to obtain satisfying results on our target datasets. The initial convolution layer incorporated 16 convolutions of size 3×3 on the input images. The number of feature-maps in all other layers follow the setting for k.

At the end of the last dense block (3rd dense block), a global average pooling was performed to minimize over-fitting by reducing the total number of parameters in the model. The final Softmax classifier of four output nodes will predict the probability for each class based on the extracted features in the network. The rest of the model's parameters with regards to the kernel, stride and padding sizes were kept as default as detailed in [42].

Training

In our experiments with LeNet and DenseNet, similar configuration has been applied as in Huang et al. [42]. Both models were trained via a stochastic gradient descent solver with the parameters set to Gamma = 0.1 (for the learning rate decreasing factor), momentum = 0.9 (for weight update from the previous iteration) and the weight-decay factor = 10^{-5}. We trained LeNet and DenseNet with mini-batches of size 64 and 8 (according to our hardware specifications), respectively. Both models were trained using an initial learning rate of 0.001 with 33% step down policy. LeNet was trained for 15 epochs and DenseNet was trained for 30 epochs. In our implementation, the LeNet and DenseNet models pretrained on the CIFAR10 dataset [34] were used to initialise the weights, whilst the networks were fine-tuned using prepared training data from the silique dataset. In the pre-processing step for each model, the mean patch calculated on the training set patches was subtracted for each sample patch being fed.

Table 2. Classification results on the validation samples.

	LeNet	DenseNet
Accuracy	80.55%	86.80%
Loss	0.64	0.37

All CNN training and testing was performed within the Caffe framework [43]. The computations were carried out using a NVIDIA GeForce GTX 1080 GPU, Intel Core i7-4790 Processor and Ubuntu 16.04 operating system.

Table 2 shows the classification accuracy and loss for both networks on the validation data from Set-1 after training.

Performance on Patch Based Classification

In the initial evaluation, we used the test samples in our model development data Set-1 to evaluate the classification and detection performance of both the shallow and deep networks. The aim of this comparative evaluation was to choose the best model for correct classification of patches and estimating silique counts on the smaller development dataset.

The classification results of both networks are presented in Tables 3 and 4 in terms of a confusion matrix, per-class accuracy and total classification accuracy. Note that only annotated patches have been considered for this evaluation. The DenseNet network showed higher representation learning capacity and thus higher accuracy to classify plant parts as compared to the LeNet network.

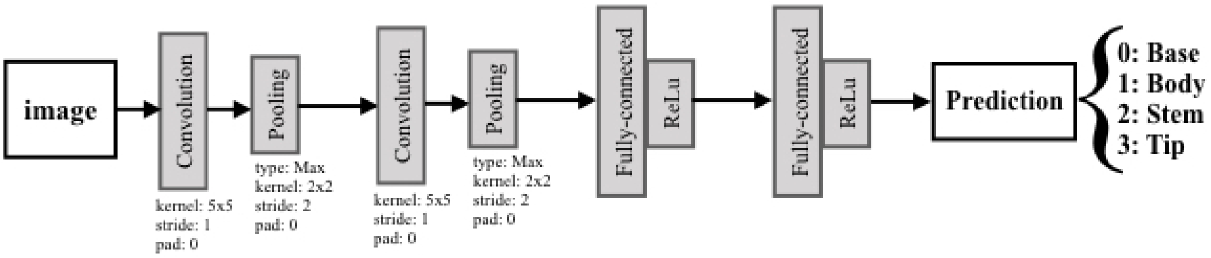

Figure 5. LeNet architecture.

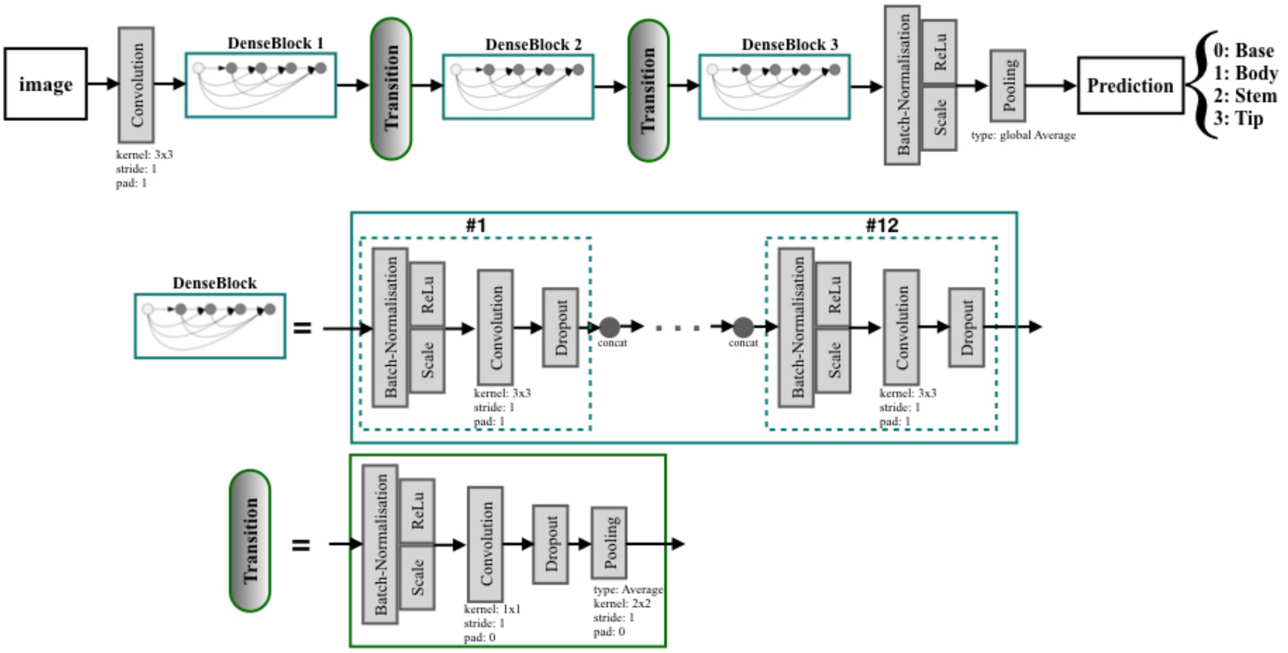

Figure 6. The DenseNet-Basic architecture used for patch based *Arabidopsis* structural part classification. The feature-map sizes in the three dense-blocks were 32×32, 16×16, and 8×8, respectively.

Table 3. Performance of patch based classification on the testing images for model development using LeNet network.

	Base	Body	Stem	Tip	Per-class accuracy
Base	344	12	52	4	83.5%
Body	15	280	26	30	79.77%
Stem	14	29	270	4	85.17%
Tip	91	31	8	169	56.52%
Accuracy = 77.08 %					

Table 4. Performance of patch based classification on the testing images for model development using DenseNet network.

	Base	Body	Stem	Tip	Per-class accuracy
Base	392	4	14	2	95.15%
Body	15	290	13	33	82.62%
Stem	11	14	290	2	91.48%
Tip	1	3	0	295	98.66%
Accuracy = 91.88 %					

Post-processing for Silique Localisation & Counting

Image Reconstruction

Given the classification of various patches in an image, post processing can be applied to reconstruct the image and detect probable silique appearances. The plant regions are first identified from the background (including borders) using simple thresholding methods. Then the plant regions are further segmented into four classes based on labelling of the patches of interest.

As the patches for a test image are generated with 50% overlap along both the horizontal and vertical direction, each patch consists of four squares of equal size (16×16), called sub-patches. Each sub-patch has four class predictions from four adjacent patches, the final decision is inferred through majority vote and the label for each pixel in the sub-patch was determined accordingly (See Figure 7). In case of a tied vote for several classes, the average probability of those classes for the image will be assigned to the sub-patch and its pixels.

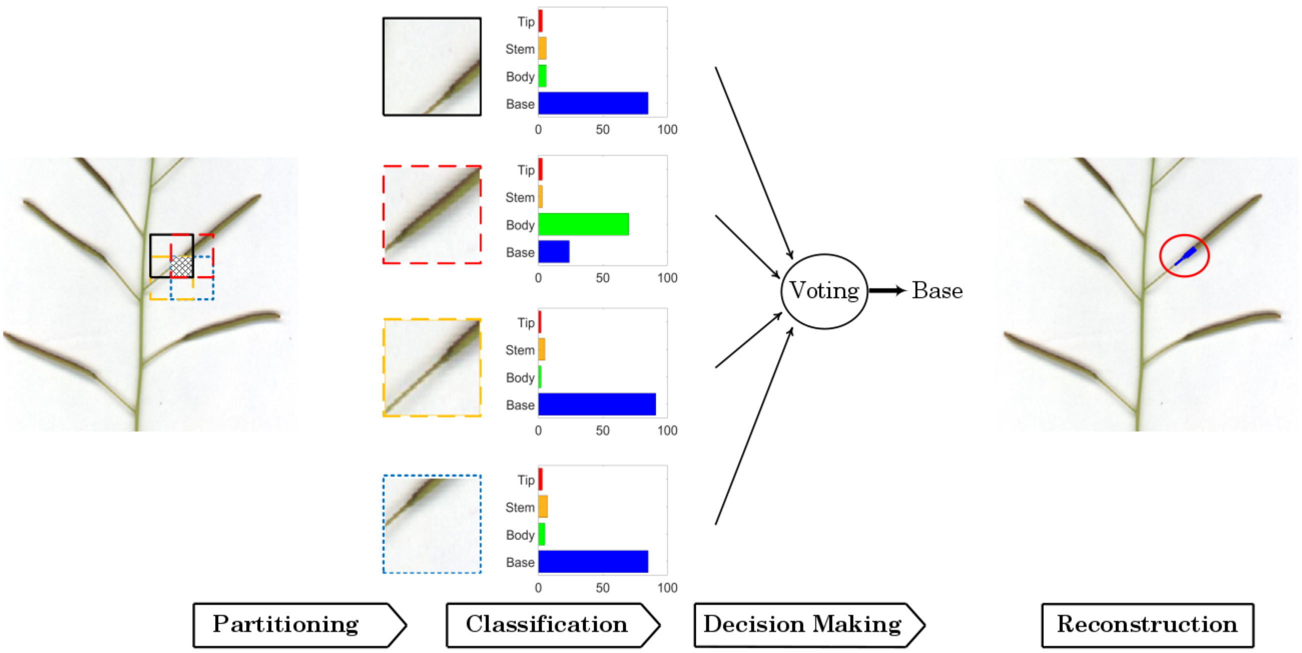

Figure 7. Flowchart of the sub-patch labelling step for image reconstruction.

Silique Counting

To count siliques in the reconstructed image, a silique is defined as an area composed of three interconnected parts: one tip, one body and one base in such a way that the body is located between the tip and the base (Figure 1). The areas where tips and bodies presenting shared borders are initially identified, these tip-body areas were extended through shared borders to search for the connected tips, which eventually established a combined area for a silique object.

In practice, many touching or overlapping siliques were observed in the captured images, which was a problem for detecting individual siliques accurately. In the case where one silique object area contained multiple tips or bases, the angle between the potentially overlaid siliques was calculated, using a cross product between the different vectors linking the bases to the corresponding tips. For example, for the case of two siliques overlaying (often with the same apparent base or tip), the centers of tips and bases were computed; then using a cross product, the centers were connected in order to calculate the angle between overlaid siliques. If the measured angle was larger than a predetermined threshold, the region was considered as two distinctive siliques, otherwise as a single silique. The value of the threshold was set to 0.05 in our experiments according to the resolution of the images.

Test results for Silique Counting

Results on the test data for model development

Figure 8 shows the results of image reconstruction for several randomly selected images after patch classification (using the DenseNet network), with colors indicating different structural parts of the plant.

Table 5 reports the performance of silique count prediction using the two different trained networks. In this table, the correlation coefficient (for the linear relationship between the prediction and the manual counts) shows that the prediction using the deeper model (DenseNet) is more accurate than using the shallower model (LeNet). This linear correlation can be better seen in Figure 9 showing the scatter plots of the actual vs automated silique counts. We also examined the distribution of the errors (actual-prediction) in silique counting, see Figure 10 for the histograms of errors for the two trained models. It appears that both LeNet and DenseNet under estimated the counts compared to manual counting in most cases.

Comparing a shallow and a deep network for classifying image patches, we concluded that the classification results and the quality of the count estimation show improvement from using the deeper architecture. Therefore, DenseNet has been selected for identifying siliques, as it appeared to be more robust to the variations in shape and size. This is probably in part a consequence of using a training set of images from diverse individuals harvested at different stages of silique maturation.

Results on the separate test data

To further evaluate the the proposed framework, we used a separate large dataset of 2,408 images available within the NPPC. The scatter plot in Figure 11 shows a high positive correlation (R^2 = 0.90) between the manual counts and automated counts. A recent computer vision approach to measure growth dynamics and fruit number consisted of skeletonization of the images with cross-validation and linear regression models, resulted in similar correlation (R^2 = 0.91) between observed and automated values on 100 individuals [2].

The CNN-based prediction tends to under-estimate compared to actual manual counting. To better understand where the problem lies, detailed detection results have also been visualised, see Figure 12 for some random examples. It seems that the current post processing method might have difficulty in detecting some small or overlapping siliques.

Discussion

Based on our test results on silique counting, we expect our method to be useful for species with similar fruit morphology such as Canola (Oilseed Rape) and other brassicas. How-

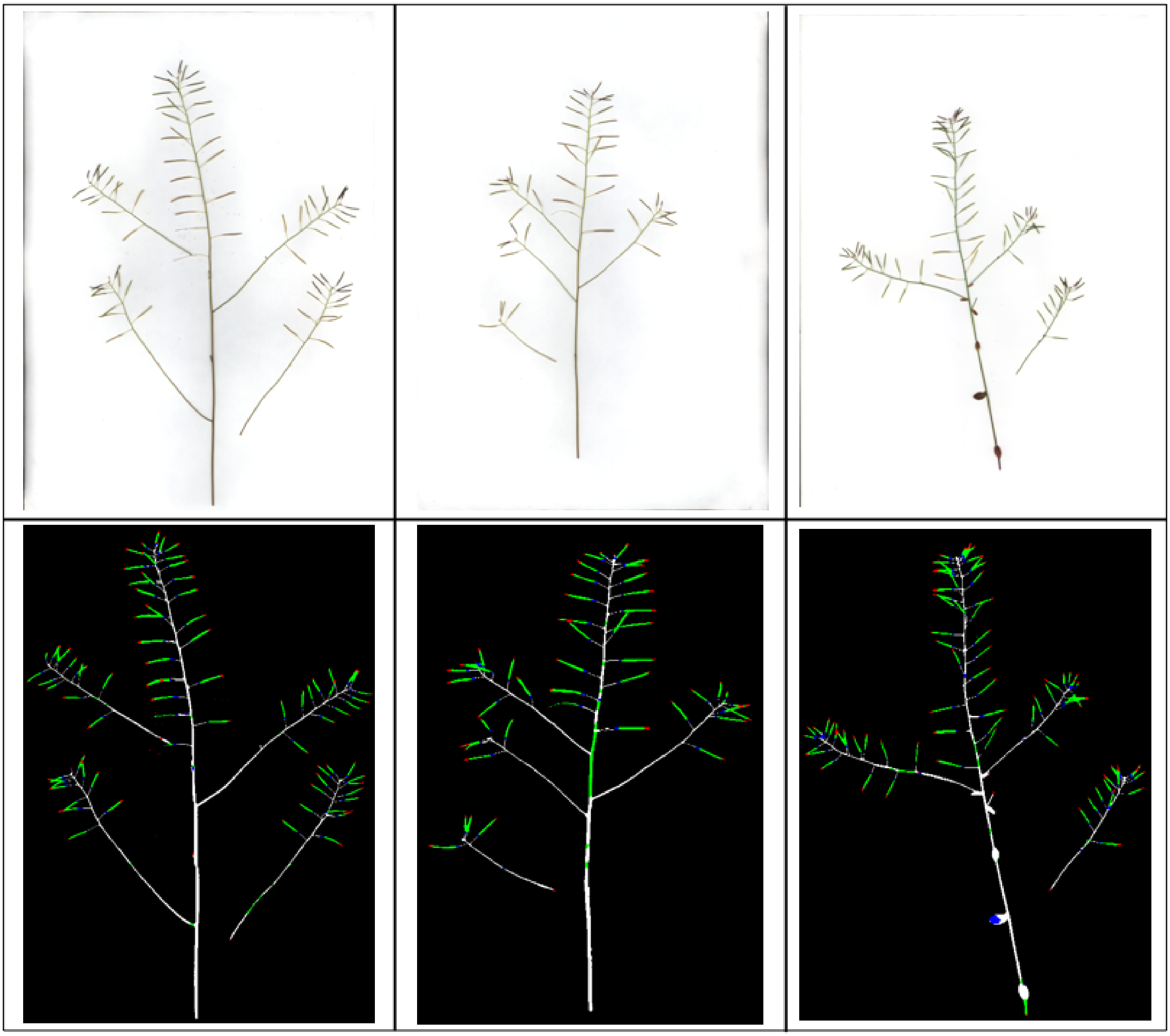

Figure 8. Three example results of labelling on the reconstructed plant images based the DenseNet patch based classification. Tips, bodies, bases and stems are indicated in red, green, blue and white, respectively.

ever, the CNN will most likely need to be fine-tuned for diverse silique morphology and imaging conditions.

There are several promising directions for future work for which the developed software can be improved such as the detection of other traits like silique length or branch number. These two traits have been reported to be a good proxy of seed number and therefore could be important for estimating productivity [44]. The following considerations should be taken into account in future to improve the classification and detection performance:

i. The robustness of the representations in both networks relied largely on the quality and quantity of the training and test data. Increased variety in the training samples (along with artificial augmentation) should provide more robust learned representations and may facilitate extension to other species.
ii. Deep learning models can take the whole image or the patches as input. In this study, a patch based classifier was used and the image was scanned over with a sliding window, classifying the patches. However, feeding all patches to the network was time-consuming and the designated patch overlap produces substantial redundancy. To overcome these issues, deep neural networks taking the whole image as input for object detection can be explored.
iii. Generative adversarial networks (GANs) [45] have been widely used in segmentation problems on real world [46, 47] and medical data (see our recent application of these models on medical images [48, 49]). To avoid the need for post-processing (which affects the performance), different types of GANs should be investigated.
iv. DeepPod can be used to accelerate the development of even more robust fruit recognition approaches. DeepPod can rapidly provide more annotated images as the output of the proposed DenseNet model can be used to automatically generate detailed fruit annotation suggestions. A human annotator would then focus on correcting false negatives (by adding missed siliques) and false positives (or removing falsely detected ones) instead of spending so much time on marking each fruit contour individually.

Table 5. Performance for silique count prediction compared to manual counting on the 22 test images for model development.

Metric	LeNet	DenseNet
Correlation coefficient	0.932	0.954
Root mean squared error	20.35	12.45

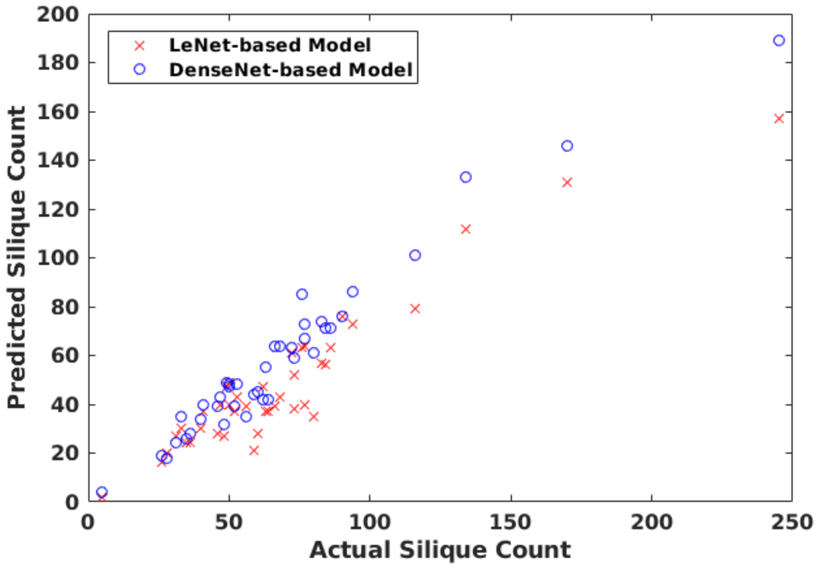

Figure 9. Automated counts using the two models using validation and testing samples. R^2 = 0.90 for the LeNet-based model and R^2 = 0.95 for the DenseNet-based model.

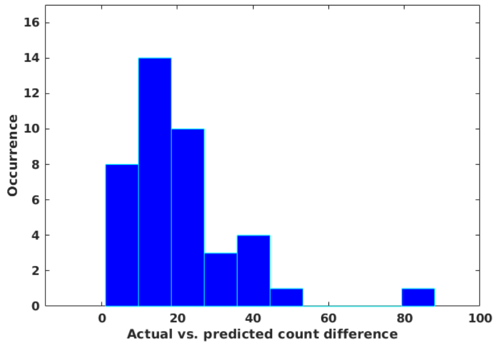

(a) Actual (manual) counts – LeNet based Prediction

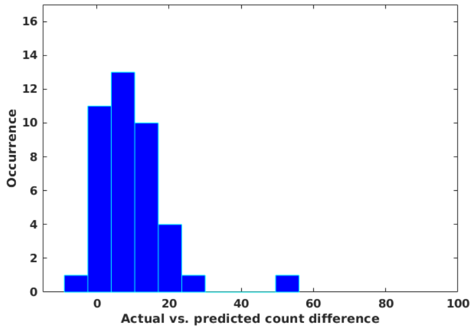

(b) Actual (manual) counts – DenseNet based Prediction

Figure 10. The histograms of errors in silique count prediction for the LeNet and DenseNet models.

Conclusion

In summary, we have developed DeepPod, an image-based deep learning framework for fruit counting. We have demonstrated DeepPod's effectiveness in silique detection and counting for *Arabidopsis*, as well as challenges due to presence of overlapping siliques and variability in fruit morphology. The pipeline developed has been shown to be cost effective in image annotation for model development. To further improve the pipeline, more robust and scale invariant methods will be investigated for object detection and for extraction of more morphological traits. Additionally active learning and transfer learning could be applied for more effective data annotation and machine learning modelling.

Availability of source code and requirements

- Project name: DeepPod
- Project home page: `https://github.com/AzmHmd/DeepPod.git`
- Operating system(s): Platform independent
- Programming language: MATLAB, Python
- Other requirements: CUDA version: 8.0, CuDNN version: v5.1, BLAS: atlas, CAFFE version: 1.0.0-rc3, DIGITS version: 5.1-dev, Python version: 2.7, MATLAB version: 9.3 or above
- License: MIT

Availability of supporting data and materials

The dataset for model development (Set-1, including 144 raw images and their annotations, and manual silique counts), and the dataset for testing (Set-2, including 2,408 raw images and their manual silique counts), are available in the Aberystwyth research data repository, DOI:10.20391/21154739-f718-457b-96ff-838408f2b696

Declarations

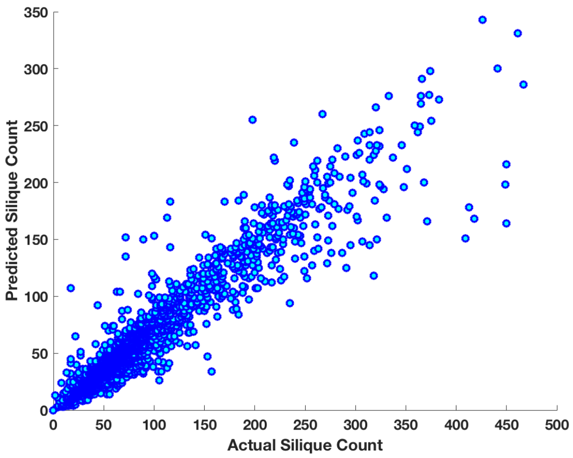

Figure 11. Automated silique count and manual counting from data Set-2 testing samples including 2,408 images. R^2 = 0.90

List of abbreviations

CNN	Convolutional Neural Network
GUI	Graphical User Interface
LSTM	Long Short Term Memory
NPPC	National Plant Phenomic Centre
RCNN	Regional CNN
SSD	Single Shot Multibox Detector
YOLO	You Only Look Once

Consent for publication

Not applicable

Competing Interests

The authors declare that they have no competing interests

Author's Contributions

J.D., F.C. and C.L designed the study and provided the images. G.G.-M. performed manual counting and manual annotations. A.H. developed the annotation toolbox, performed the deep learning and data analysis, testing and evaluation tasks. A.H. and M.G. carried out the post processing analysis. AH and G.G.-M. drafted the manuscript. All the authors provided comments and corrected the manuscript.

Acknowledgements

The authors would like to gratefully acknowledge Sandy Spence and Alun Jones for their support and maintenance of the GPU and the systems used for this research. We also acknowledge the team of the National Plant Phenomics Centre for the image dataset, mainly to Lina Avila Clasen for her help in acquiring the images and manual counting. GG-M acknowledges receipt of a AberDoc scholarship from Aberystwyth University; JHD, FC and CL funding from BBSRC (grants BB/CAP1730/1, BB/P013376/1 and BB/P003095/1).

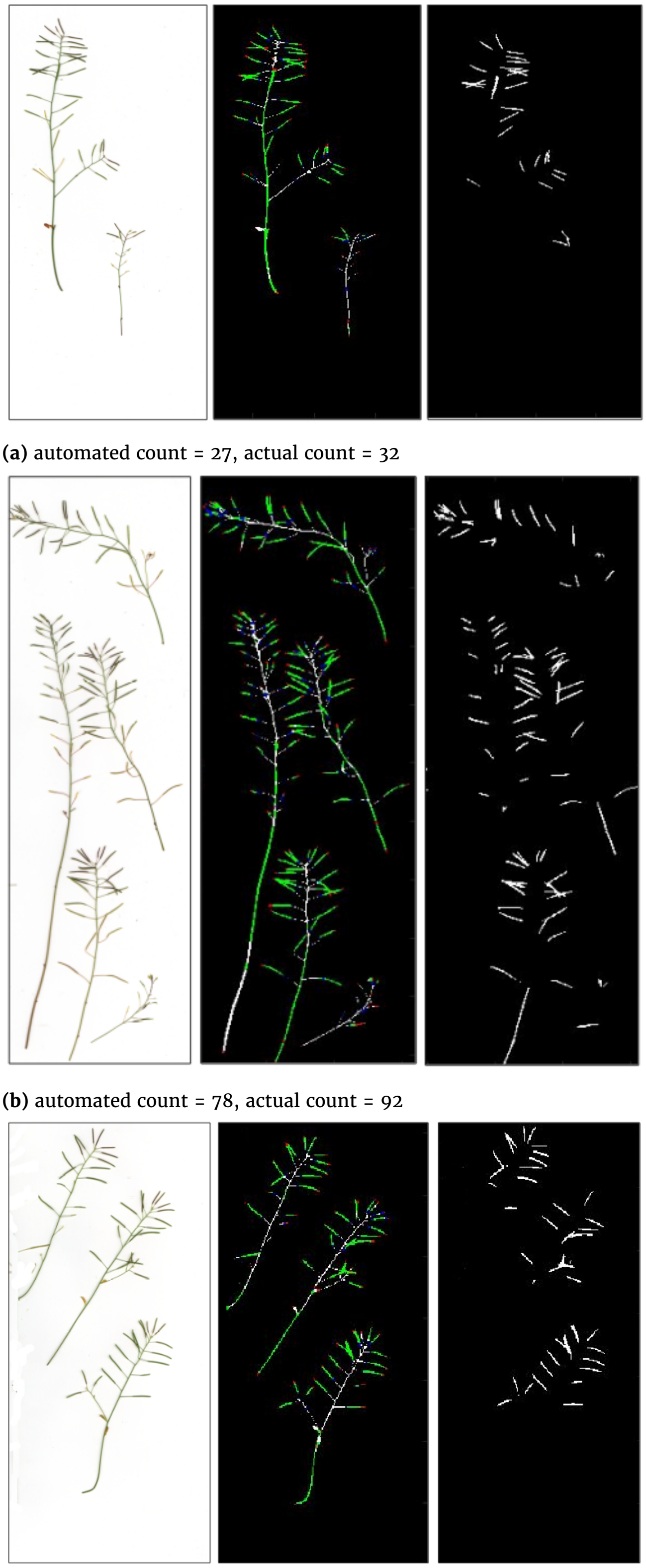

(a) automated count = 27, actual count = 32

(b) automated count = 78, actual count = 92

(c) automated count = 40, actual count = 52

Figure 12. Results of the DenseNet framework applied to some random samples from the larger testing dataset. From left to right: original plant images, sub-patch labelling and image reconstruction (Tips, bodies, bases and stems are indicated in red, green, blue and white, respectively), silique region detection (in white).

References

1. Chen D, Neumann K, Friedel S, Kilian B, Chen M, Altmann T, et al. Dissecting the phenotypic compo-

nents of crop plant growth and drought responses based on high-throughput image analysis. The Plant Cell 2014;26(12):4636–4655.
2. Vasseur F, Bresson J, Wang G, Schwab R, Weigel D. Image-based methods for phenotyping growth dynamics and fitness components in Arabidopsis thaliana. Plant Methods 2018;14(1):63.
3. Furbank RT, Tester M. Phenomics–technologies to relieve the phenotyping bottleneck. Trends in Plant Science 2011;16(12):635–644.
4. Pauli D, Andrade-Sanchez P, Carmo-Silva AE, Gazave E, French AN, Heun J, et al. Field-based high-throughput plant phenotyping reveals the temporal patterns of quantitative trait loci associated with stress-responsive traits in cotton. G3: Genes, Genomes, Genetics 2016;6(4):865–879.
5. Shakoor N, Lee S, Mockler TC. High throughput phenotyping to accelerate crop breeding and monitoring of diseases in the field. Current Opinion in Plant Biology 2017;38:184–192.
6. Camargo AV, Mott R, Gardner KA, Mackay IJ, Corke F, Doonan JH, et al. Determining phenological patterns associated with the onset of senescence in a wheat MAGIC mapping population. Frontiers in Plant Science 2016;7:1540.
7. Liebisch F, Kirchgessner N, Schneider D, Walter A, Hund A. Remote, aerial phenotyping of maize traits with a mobile multi-sensor approach. Plant Methods 2015;11(1):9.
8. Singh A, Ganapathysubramanian B, Singh AK, Sarkar S. Machine learning for high-throughput stress phenotyping in plants. Trends in Plant Science 2016;21(2):110–124.
9. Pape JM, Klukas C. Utilizing machine learning approaches to improve the prediction of leaf counts and individual leaf segmentation of rosette plant images. Proceedings of the Computer Vision Problems in Plant Phenotyping (CVPPP) 2015;p. 1–12.
10. Naik HS, Zhang J, Lofquist A, Assefa T, Sarkar S, Ackerman D, et al. A real-time phenotyping framework using machine learning for plant stress severity rating in soybean. Plant Methods 2017;13(1):23.
11. Atkinson JA, Lobet G, Noll M, Meyer PE, Griffiths M, Wells DM. Combining semi-automated image analysis techniques with machine learning algorithms to accelerate large scale genetic studies. GigaScience 2017;.
12. Arinkin V, Digel I, Porst D, Artmann AT, Artmann GM. Phenotyping date palm varieties via leaflet cross-sectional imaging and artificial neural network application. BMC Bioinformatics 2014;15(1):55.
13. Pound MP, Atkinson JA, Townsend AJ, Wilson MH, Griffiths M, Jackson AS, et al. Deep machine learning provides state-of-the-art performance in image-based plant phenotyping. Gigascience 2017;6(10):gix083.
14. Mohanty SP, Hughes DP, Salathé M. Using deep learning for image-based plant disease detection. Frontiers in Plant Science 2016;7.
15. Ubbens J, Cieslak M, Prusinkiewicz P, Stavness I. The use of plant models in deep learning: an application to leaf counting in rosette plants. Plant Methods 2018;14(1):6.
16. Namin ST, Esmaeilzadeh M, Najafi M, Brown TB, Borevitz JO. Deep phenotyping: deep learning for temporal phenotype/genotype classification. Plant Methods 2018;14(1):66.
17. Ubbens JR, Stavness I. Deep plant phenomics: a deep learning platform for complex plant phenotyping tasks. Frontiers in Plant Science 2017;8:1190.
18. Pawara P, Okafor E, Surinta O, Schomaker L, Wiering M. Comparing Local Descriptors and Bags of Visual Words to Deep Convolutional Neural Networks for Plant Recognition. In: ICPRAM; 2017. p. 479–486.
19. Fuentes A, Yoon S, Kim SC, Park DS. A robust deep-learning-based detector for real-time tomato plant diseases and pests recognition. Sensors 2017;17(9):2022.
20. Wang G, Sun Y, Wang J. Automatic image-based plant disease severity estimation using deep learning. Computational Intelligence and Neuroscience 2017;2017.
21. Ramcharan A, Baranowski K, McCloskey P, Ahamed B, Legg J, Hughes D. Transfer Learning for Image-Based Cassava Disease Detection. Frontiers in Plant Science 2017;8:1852.
22. Mitchell-Olds T. Arabidopsis thaliana and its wild relatives: a model system for ecology and evolution. Trends in Ecology & Evolution 2001;16(12):693–700.
23. Koornneef M, Meinke D. The development of Arabidopsis as a model plant. The Plant Journal 2010;61(6):909–921.
24. Krämer U, Planting molecular functions in an ecological context with Arabidopsis thaliana. PubMed PMID: 25807084; PubMed Central PMCID: PMC4373673.; 2015.
25. Reboud X, Le Corre V, Scarcelli N, Roux F, David J, Bataillon T, et al. Natural variation among accessions of Arabidopsis thaliana: beyond the flowering date, what morphological traits are relevant to study adaptation. Plant Adaptation: Molecular Genetics and Ecology, edited by QCB Cronk, J Whitton, RH Ree and IEP Taylor NRC Research Press, Ottawa, Ontario, Canada 2004;p. 135–142.
26. Bac-Molenaar JA, Vreugdenhil D, Granier C, Keurentjes JJ. Genome-wide association mapping of growth dynamics detects time-specific and general quantitative trait loci. Journal of Experimental Botany 2015;66(18):5567–5580.
27. Bac-Molenaar JA, Granier C, Keurentjes JJ, Vreugdenhil D. Genome-wide association mapping of time-dependent growth responses to moderate drought stress in Arabidopsis. Plant, Cell & Environment 2016;39(1):88–102.
28. Minervini M, Abdelsamea MM, Tsaftaris SA. Image-based plant phenotyping with incremental learning and active contours. Ecological Informatics 2014;23:35–48.
29. Augustin M, Haxhimusa Y, Busch W, Kropatsch WG. A framework for the extraction of quantitative traits from 2D images of mature Arabidopsis thaliana. Machine Vision and Applications 2016;27(5):647–661.
30. Bush MS, Crowe N, Zheng T, Doonan JH. The RNA helicase, eIF 4A-1, is required for ovule development and cell size homeostasis in Arabidopsis. The Plant Journal 2015;84(5):989–1004.
31. Zheng T, Nibau C, Phillips DW, Jenkins G, Armstrong SJ, Doonan JH. CDKG1 protein kinase is essential for synapsis and male meiosis at high ambient temperature in Arabidopsis thaliana. Proceedings of the National Academy of SSciences 2014;111(6):2182–2187.
32. LeCun Y, Kavukcuoglu K, Farabet C. Convolutional networks and applications in vision. In: Proceedings of IEEE International Symposium on Circuits and Systems (ISCAS),; 2010. p. 253–256.
33. Simonyan K, Zisserman A. Very deep convolutional networks for large-scale image recognition. International Conference on Learning Representations, arXiv preprint arXiv:14091556 2014;.
34. Krizhevsky A, Hinton G. Learning multiple layers of features from tiny images. Technical Report, University of Toronto 2009;(4).
35. Krizhevsky A, Sutskever I, Hinton GE. Imagenet classification with deep convolutional neural networks. In: Advances in Neural Information Processing Systems; 2012. p. 1097–1105.
36. Szegedy C, Liu W, Jia Y, Sermanet P, Reed S, Anguelov D, et al. Going deeper with convolutions. In: Proceedings of the IEEE Conference on Computer Vision and Pattern Recognition; 2015. p. 1–9.
37. Redmon J, Divvala S, Girshick R, Farhadi A. You only look

once: Unified, real-time object detection. In: Proceedings of the IEEE Conference on Computer Vision and Pattern Recognition; 2016. p. 779–788.
38. Zhang C, Bengio S, Hardt M, Recht B, Vinyals O. Understanding deep learning requires rethinking generalization. arXiv preprint arXiv:161103530 2016;.
39. Hamidinekoo A, Suhail Z, Qaiser T, Zwiggelaar R. Investigating the effect of various augmentations on the input data fed to a convolutional neural network for the task of mammographic mass classification. In: Annual Conference on Medical Image Understanding and Analysis Springer; 2017. p. 398–409.
40. LeCun Y, Bengio Y, Hinton G. Deep learning. Nature 2015;521(7553):436–444.
41. LeCun Y, Bottou L, Bengio Y, Haffner P. Gradient-based learning applied to document recognition. Proceedings of the IEEE 1998;86(11):2278–2324.
42. Huang G, Liu Z, van der Maaten L, Weinberger KQ. Densely connected convolutional networks. In: Proceedings of the IEEE Conference on Computer Vision and Pattern Recognition; 2017. .
43. Jia Y, Shelhamer E, Donahue J, Karayev S, Long J, Girshick R, et al. Caffe: Convolutional architecture for fast feature embedding. In: Proceedings of the 22nd ACM International Conference on Multimedia ACM; 2014. p. 675–678.
44. Bac-Molenaar JA, Fradin EF, Becker FF, Rienstra JA, van der Schoot J, Vreugdenhil D, et al. Genome-wide association mapping of fertility reduction upon heat stress reveals developmental stage-specific QTLs in Arabidopsis thaliana. The Plant Cell 2015;p. tpc–15.
45. Goodfellow I, Pouget-Abadie J, Mirza M, Xu B, Warde-Farley D, Ozair S, et al. Generative adversarial nets. In: Advances in Neural Information Processing Systems; 2014. p. 2672–2680.
46. Isola P, Zhu JY, Zhou T, Efros AA. Image-to-image translation with conditional adversarial networks. In: Proceedings of the IEEE Conference on Computer Vision and Pattern Recognition; 2017. p. 1125–1134.
47. Luc P, Couprie C, Chintala S, Verbeek J. Semantic segmentation using adversarial networks. arXiv preprint arXiv:161108408 2016;.
48. Hamidinekoo A, Denton E, Rampun A, Honnor K, Zwiggelaar R. Deep learning in mammography and breast histology, an overview and future trends. Medical Image Analysis 2018;47:45–67.
49. Grall A, Hamidinekoo A, Malcolm P, Zwiggelaar R. Using a conditional Generative Adversarial Network (cGAN) for Prostate Segmentation. In: 23rd Conference on Medical Image Understanding and Analysis; 2019. .
