# Supplementary material for: DeepPod: a convolutional neural network based quantification of fruit number in Arabidopsis
Source: Gigascience. 2020 Mar 4;9(3):giaa012. doi: 10.1093/gigascience/giaa012 (PMC7055469; doi:10.1093/gigascience/giaa012)
Supplement: giaa012_GIGA-D-19-00251_Revision_1 [file giaa012_giga-d-19-00251_revision_1.pdf]

GigaScience

DeepPod: A Convolutional Neural Network Based Quantification of Fruit Number in Arabidopsis

--Manuscript Draft--

**Manuscript Number:**	GIGA-D-19-00251R1
**Full Title:**	DeepPod: A Convolutional Neural Network Based Quantification of Fruit Number in Arabidopsis
**Article Type:**	Technical Note
**Funding Information:**	Biotechnology and Biological Sciences Research Council (BB/CAP1730/1) — Prof John H. Doonan<br>Biotechnology and Biological Sciences Research Council (BB/P003095/1) — Prof John H. Doonan<br>Aberystwyth University (AberDoc Scholarship) — Ms Azam Hamidinekoo, Ms Gina A. Garzón-Martínez, Mr Morteza Ghahremani<br>Biotechnology and Biological Sciences Research Council (BB/P013376/1) — Prof John H. Doonan
**Abstract:**	High-throughput phenotyping based on non-destructive imaging has great potential in plant biology and breeding programs. However, efficient feature extraction and quantification from image data remains a bottleneck that needs to be addressed. Advances in sensor technology have led to the increasing use of imaging to monitor and measure a range of plants including the model Arabidopsis thaliana. These extensive datasets contain diverse trait information but feature extraction is often still implemented using approaches requiring substantial manual input. The computational detection and segmentation of individual fruits from images is a challenging task, for which we have developed DeepPod, a patch based two-phase deep learning framework. The associated manual annotation task is simple and cost effective without the need for detailed segmentation or bounding boxes. Convolutional neural networks (CNNs) are used for classifying different parts of the plant inflorescence, including the tip, base and body of the siliques and the stem inflorescence. In a post processing step, different parts of the same silique are joined together for silique detection and localisation, whilst taking into account possible overlapping among the siliques. The proposed framework is further validated on a separate test dataset of 2,408 images. Comparisons of the CNN based prediction with manual counting (R2 = 0.90) showed the desired capability of methods for estimating silique number.
**Corresponding Author:**	Chuan Lu<br>Aberystwyth University<br>Aberystwyth, Ceredigion UNITED KINGDOM
**Corresponding Author Secondary Information:**	
**Corresponding Author's Institution:**	Aberystwyth University
**Corresponding Author's Secondary Institution:**	
**First Author:**	Azam Hamidinekoo
**First Author Secondary Information:**	
**Order of Authors:**	Azam Hamidinekoo
	Gina A. Garzón-Martínez
	Morteza Ghahremani
	Fiona M. K. Corke

	Reyer Zwiggelaar
	John H. Doonan
	Chuan Lu
**Order of Authors Secondary Information:**	

Response to Reviewers:

Dear Dr. Nogoy:

We are very grateful to the reviewers for their positive and constructive comments on our manuscript. Their valuable suggestions have helped us to improve the manuscript. We have updated the manuscript accordingly and provide below point-by-point responses to the reviewers' comments.

We hope that you find the revised manuscript acceptable for publication in GigaScience.

Thank you for your consideration.

Sincerely,

Chuan Lu, on behalf of the co-authors
Aberystwyth University, UK

Reviewer #1:

Reviewer #1: In this Technical Note the authors report on DeepPod, which is image analysis software that utilises Convolutional Neural Network (CNN) architecture to classify Arabidopsis thaliana plant components into 4 classes. A key feature of the DeepPod image analysis process is the use of patch-based classification to detect and count fruits of this plant. This was followed by the use of CNN-based classifiers to extract patches of interest, and to output the probability that these patches contain any of four components, namely base, body, stem, or tip. The manuscript is well written, and the DeepPod software is available on GitHub with an OSI-approved MIT license that ensures that the software is open and accessible. Images used for manual annotation and training the convolutional neural network, and for testing the performance of the model, are available from a stable DOI link at Aberystwyth University.

- 1. DeepPod software requires Python 2.7, which will not be supported beyond 2020. More details about this are available at the following link: https://pythonclock.org/ As reuse is a major objective of GigaScience, I invite the authors to provide a detailed plan of how they will ensure continued support for DeepPod software in the longer term. For long-term reusability, the authors should consider updating the code to a version of Python 3 that will be supported long term.

Thank you for your comments on this important issue on reusability. We have revised the software requirements for DeepPod that excludes python. The Python scripts (which could be run in Python 3 as well) were only used for generating performance tables for the manuscript, but not required in the DeepPod pipeline. DeepPod requires Caffe, a deep learning framework for CNN classificatin model training and prediction, and Matlab for image annotation, data preparation as well as image reconstruction and silique counting.

Moreover, DeepPod is an ongoing project for which we will provide longer term support and undertake further development both in methodology and software. We believe that DeepPod, as an open source project hosted on GitHub with annotated data freely available, will invite more contributions from the community and make it more sustainable in the future.

- 2. Some of the image files that are publicly available at DOI:10.20391/21154739-f718-457b-96ff-838408f2b696 cannot be opened using ImageJ/Fiji. The authors need to provide md5 checksum values for all image files so that I can identify whether this is

a file transfer issue with the 13GB Set-2 dataset, or whether there are corrupt files in the Set-2 directory of 2,408 raw images.

Thank you for your valuable comments. This issue has been sorted with the updated data download webpage, where md5 checksum values have been provided for both images sets.

- Minor issue 1. The images and readme file on DOI:10.20391/21154739-f718-457b-96ff-838408f2b696 have been ascribed a CC-BY 4.0 license, and therefore GigaScience cannot archive a copy of these data. To ensure reuse, the authors should consider ascribing a CC0 license to these data.

The license from from Set_1 (144 images for model development) and readme file have been changed to CC0. Set-2 had been registered with CC-BY 4.0. All files are available in the Aberystwyth Research Repository with DOI number: 10.20391/21154739-f718-457b-96ff-838408f2b696. Also, Set_1 will be available at GigaDB and a link to Set_2 will be reference in the GigaDB readme.

- 2. There are spaces in the image filenames. These filenames - with spaces - are additionally referred to in the Set-1_Manual_counting.csv and Set-2_manual.csv files. It is the recommendation of the GigaScience Database that filenames do not include spaces (see http://gigadb.org/site/guide).

Fixed. All the spaces from folders and files were replaced with an underline.

Reviewer #2:

In the study, Hamidinekoo et al. developed a deep learning-based method (DeepPod) to count fruit number. Overall, the paper is well written and is easy to follow.
Some minor comments:

- 1) The authors compared the performance of LeNet and DenseNet (Tables 2-5 and Figs 9-10). Is it possible to use the ROC metric show the overall performance?

Table 3 provided the accuracy and loss for the validation data during CNN training, which will not be used as a generalisation performance estimate and therefore no further ROC analysis has been performed on this validation data during training. We have updated Table 4 and 5: besides confusion tables for the four classes, we have also added precision and recall rate for different classes, which should serve as better performance measure compared to accuracy alone. The final overall performance of the pipeline will be evaluated on the silique counting problem using separate test dataset.

- 2) To compare the prediction by LeNet and DenseNet, a similar plot like Fig. 8 is required to show the output of LeNet.

LeNet performed significantly worse than DenseNet on patch classification (total recall of 0.76 and precision of 0.78 on the development test set, in comparison to recall of 0.92 and precision of 0.92 for DenseNet, see Table 4 and 5). Hence only the DenseNet model-based pipeline has been chosen for further evaluation and visual inspection.

- 3) In Figs 9 and 11, it would be useful to add fitted lines and correlation coefficient values in the plots.

Thank you for your valuable suggestion. Figure 9 and 11 have been updated to add fitted lines and the correlation coefficients in the plots.

- 4) How was the manual counting data in Fig 11 obtained? I didn't find relevant information from the main text.

We have added details about manual counting to the end of the section on Data Acquisition (page 3). “Manual counting of viable fruits in images was undertaken by a single person to minimize operator variation. ImageJ [39] was used to track the

counting by setting a label to each fruit as it was counted”

Reviewer #3:

This paper describes the development of a deep learning plant phenotyping system. Specifically, the system uses a CNN approach to detecting and counting fruit (seeds/siliques) of the model plant Arabidopsis. A substantial dataset from the National Plant Phenomics Centre is used to build and test the networks. Two network architectures are tested, based on existing formats.

The paper is interesting, but the contribution from the deep learning aspects of the paper are in my opinion small. Two existing (albeit tweaked) network architectures are used and evaluated. One of these is very old. I can not see any novelty in the deep learning application here, so it is either unclear or does not exist. That is not to say there is no novelty in the paper as a whole. The application domain is relevant to plant phenotyping/food security, and I have seen very little existing research on silique detection/counting compared to, say, leaves or roots. The dataset itself is large and will be of value when released. The post-processing steps to reconstruct siliques from patches is novel.

- Major points:
* Why is detecting of the base/stem/body/tip necessary? Why not just detect "siliques" as a whole? This would remove the need for the post processing.

We have now explained our motivation behind the choice of the two-phase approach over the alternative one that detects the siliques as a whole in the last three paragraphs of the Background section. The main reason is that, “training of such networks require labelled data with detailed segmentation or bounding boxes of individual objects, which are obtained usually through a very tedious manual process. Moreover, the image size allowed for the network input is limited due to the complexity of network architecture and the available memory.”

There are other reasons for separate detection of the component parts, mainly concerned with future development and refinement of the platform. This will allow us (and hopefully others) to continue investigating alternative approaches that could improve the pipeline for fruit counting and other relevant morphological feature extraction, with more annotated data being available in the future.

- * Following this, the advantage of detecting tips/bases is that silique length can be measured (as mentioned in the future work section). I would suggest this would be a much stronger paper if the processing for doing this was included here. This would justify the detection of sub-features of the siliques, and also provide more novel software development. It would also add much more power to the DeepPod system, making it more useful for the phenotyping community. Given the authors already have the detection system and annotations, only the image processing steps remain to be developed and evaluated.

Thank you for the suggestion. We do have estimates of silique length for all the test examples in Set-2, and have now provided the results (including the mean and range of the silique length) in CSV file as Supplemetary Data S1.

- Minor points:
-LeNet is now a very dated architecture. Please justify better why you are using this as one of the two comparison architectures.

We have added justification for the choice of LeNet architecture to the section on Network Architecture. “LeNet is a simple shallow network with only two convolutional layers, and has been chosen as a baseline model in this study, considering the potentially higher computational resource needs for running more complex deep learning models.”

- Was the data collected specifically for this paper or was it already in existence?

Powered by Editorial Manager® and ProduXion Manager® from Aries Systems Corporation

Please make this more clear. Also, whilst the scanning setup is detailed, descriptions of the growth conditions etc. seem to be missing, and may be helpful if added. Also, I believe all data should be deposited at Gigascience rather than on institution servers (I assume this will happen anyway).

The image data set was pre-existing, having been produced as part of an on-going collaboration with Dr Biernaskie, University of Oxford. The identity of the plant material (i.e. the "RIL" number) is embedded within the image file names, where the first three numbers after the name of the experiment (eg. AT0XX_152XXX) corresponds to a subset of RILs described by Kover et al. 2009. This is now explained both in the paper as well as the on-line DOI. Details of growth conditions have been added (See Data acquisition section, page 3). Dr Biernaskie is now acknowledged in the text. The data will also be available through Gigascience.

- Two datasets are used, referred to as Set-1 (144 images, manually annotated, used for training) and Set-2(2408 images, used for final testing.) Please clarify, was the final test set (Set-2) annotated too, then? The dataset description on p3 makes it sound like only Set-1 is annotated. If it is not annotated, how exactly is Set-2 used for testing? Later text states "Set-1...was split into train, validation and test sets...". Again it is not clear how Set-2 is going to be used. (To note, further on, on p7, Set-2 is used as a further test set - which means it must be annotated or at least has a manual "inspection" count - please can you detail which of these is the case.)

Set-2 was not annotated to provide labels of silique structural elements (base, tip, stem and body). The structural element labelling was only provided for Set-1 , which was used to train and validate the patch based classification model. However Set-2 has been given manual silique counts for each image. This has been clarified by providing additional details in Table 1 on available annotation and their use in this work for different datasets.

-much of the approach using images patches (including the scanning window, and sub cropping within a larger window for augmentation etc) is similar to our previous work [13]. This is referenced in the introduction, but it might be helpful to also point to this paper in the methods section as there is a lot of similarity in the basic approach.

We have added the reference [13] to the Method section for data preparation and model development, and pointed out that similar approaches have been followed here as in [13] for image patch generation and augmentation.

-Also to note: The authors have referenced our previous work [13] second column p2, but don't quite have the details right. It is not a shallow CNN with 2 x conv layers (please see Supplemental 2 in [13] for full architecture). Please also add details explaining how the approach here is different from the existing approach.

We have revised the manuscript to describe the existing work in [13] in more detail, in particular, to better explain on how the existing approach differs from ours. See the revised paragraph in the Background Section below.

"Pound et al. [13] demonstrated wheat root and shoot feature identification and localisation using two different standard CNN architectures for patch classification. For shoot features, they found that the leaf tips represented the hardest classification problem compared to the leaf base due to the existing variations in orientation, size, shape and colour of tips in their dataset. Further reconstruction from the classification results of overlapping patches allows localisation of separate structural regions such as leaf tips and bases. However, the objects of interest as a whole (such as leaves) are yet to be identified in order to extract more morphological features (e.g. leaf length and shape). "

-p5. What does the sentence "Note that only annotated patches have been considered for evaluation" mean? Are some not annotated?

Our annotation approach does not require detailed segmentation of the images, only

Powered by Editorial Manager® and ProduXion Manager® from Aries Systems Corporation

pixel/point sampling for the main structural regions are required. Although most tips and bases have been annotated, only a small portion of patches for body or stems have been labelled (see Figure 3). Also we exclude patches in the background for classification in order to speed up the process for image scanning and reconstruction process. This makes our pipeline actually more cost effective in annotation.

-p5,. "DenseNet showed higher representation learning capacity" - is there evidence for this, or is it a hypothesis?

We have revised the wording to avoid confusion here. “The DenseNet network has higher representational power due to its deeper architecture and its use of features of multiple levels for classification in comparison to the LeNet network; its efficacy in the learning task has also been evidenced by its higher accuracy in classifying plant parts (as shown in Tables 4 and 5).”

- it would be helpful to have a figure (or further supplemental info) illustrating the strategy for dealing with overlapping siliques (p7, "Sillique counting")

Thank you for your valuable suggestion. We have added Supplementary Figure S1 to provide an illustrative example with more details on our strategy for detection and counting siliques with overlapping regions.

-Table 5. It may be more insightful to have some more metrics, e.g. % exactly right, % within 1 count of the groundtruth, % within 5 counts of GT etc. as correlation can be hard to interpret, and is sensitive to outliers (e.g. the the right-most three points in fig 9 may possibly be skewing the correlation)

We have modified Figure 10, the histograms for prediction errors (actual count – predicted count) with finer bins (<5), which will provide a better idea on the distribution of the errors.

- In the results (p7) it seems like a recent non-deep learning approach actually performed better (r^2 0.91 versus 0.9). This definitely warrants further discussion.

We have added more discussion on the non-deep learning approach and how it compares to ours. And their performance of R2=0.91 is only achieved on the cross-validation, when applied on a separate experiment, the R2 actually dropped to 0.7. See below the paragraph added to the Discussion section.

“A recent computer vision approach [2] to fruit number estimation involves linear regression using selected skeleton descriptors (such as junction numbers and number of triple points) extracted after segmentation and 2D skeletonisation, resulted in a Pearson correlation coefficient (R2) of 0.91 between observed and automated values for the best performing model on 100 cross-validated examples. When applied on the dataset from a separate experiment, the model prediction can qualitatively capture the main phenotype under investigation, however the correlation of the prediction with manual counts R2 dropped to 0.7 [2]. This suggests that the regression approach to fruit counting might not be generalized to other conditions as effectively as our object recognition approach. Apparently, the non-deep learning approach used only “handcrafted” global features with resulting models more specific to the conditions for training, whilst our approach used both local features (for patch classification) as well as more global features (for object reconstruction).

-Is the annotation GUI being released?

This toolbox is now registered in the www.SciCrunch.org data sharing and display platform with the Research Resource Identification Initiative ID (RRID) number of “SCR_017413” , under the name of “Plant Phenotyping AnnotationToolbox” . This information is added to the section on source code availability.

-p2 "augment [an] Arabidopsis rosette dataset" (wording)

Fixed.

	-p3 "the difference in distribution between testing and training"... please clarify which difference in which distribution you are referring too. (density)<br><br>We are referring to the pixel density distribution here. This has been clarified in the revision.<br><br>-p3 " (3) to exclude ambiguous patch examples" - sorry I'm not sure of the meaning here.<br><br>We have removed this statement in the revision.<br>--
**Additional Information:**	
**Question**	**Response**
Are you submitting this manuscript to a special series or article collection?	No
**Experimental design and statistics**<br><br>Full details of the experimental design and statistical methods used should be given in the Methods section, as detailed in our Minimum Standards Reporting Checklist. Information essential to interpreting the data presented should be made available in the figure legends.<br><br>Have you included all the information requested in your manuscript?	Yes
**Resources**<br><br>A description of all resources used, including antibodies, cell lines, animals and software tools, with enough information to allow them to be uniquely identified, should be included in the Methods section. Authors are strongly encouraged to cite Research Resource Identifiers (RRIDs) for antibodies, model organisms and tools, where possible.<br><br>Have you included the information requested as detailed in our Minimum Standards Reporting Checklist?	Yes
**Availability of data and materials**	Yes

All datasets and code on which the conclusions of the paper rely must be either included in your submission or deposited in publicly available repositories (where available and ethically appropriate), referencing such data using a unique identifier in the references and in the “Availability of Data and Materials” section of your manuscript.<br><br>Have you have met the above requirement as detailed in our Minimum Standards Reporting Checklist?	

Manuscript Revision1

Click here to view linked References

Click here to access/download;Manuscript;DeepPod_manuscript_R1.pdf

OXFORD

Placeholder for journal logo gigascience-logo.pdf

GigaScience, 2019, 1–13

doi: xx.xxxx/xxxx
Manuscript in Preparation
Paper

PAPER

DeepPod: A Convolutional Neural Network Based Quantification of Fruit Number in *Arabidopsis*

Azam Hamidinekoo[1,*,†], Gina A. Garzón-Martínez[2,*,†], Morteza Ghahremani[1,2], Fiona M. K. Corke[2], Reyer Zwiggelaar[1], John H. Doonan[2] and Chuan Lu[1, *, ‡]

[1]Department of Computer Science, Aberystwyth University, Aberystwyth, United Kingdom and [2]National Plant Phenomics Centre, Institute of Biological, Environmental and Rural Sciences, Aberystwyth University, Aberystwyth, United Kingdom

[*]azh2@aber.ac.uk; gig7@aber.ac.uk; cul@aber.ac.uk
[†]Contributed equally.
[‡]Corresponding author.

Abstract

High-throughput phenotyping based on non-destructive imaging has great potential in plant biology and breeding programs. However, efficient feature extraction and quantification from image data remains a bottleneck that needs to be addressed. Advances in sensor technology have led to the increasing use of imaging to monitor and measure a range of plants including the model *Arabidopsis thaliana*. These extensive datasets contain diverse trait information but feature extraction is often still implemented using approaches requiring substantial manual input. The computational detection and segmentation of individual fruits from images is a challenging task, for which we have developed DeepPod, a patch based two-phase deep learning framework. The associated manual annotation task is simple and cost effective without the need for detailed segmentation or bounding boxes. Convolutional neural networks (CNNs) are used for classifying different parts of the plant inflorescence, including the tip, base and body of the siliques and the stem inflorescence. In a post processing step, different parts of the same silique are joined together for silique detection and localisation, whilst taking into account possible overlapping among the siliques. The proposed framework is further validated on a separate test dataset of 2,408 images. Comparisons of the CNN based prediction with manual counting (R^2 = 0.90) showed the desired capability of methods for estimating silique number.

Key words: Plant phenotyping; image analysis; deep learning; object detection; fruit counting; *Arabidopsis*

Introduction

Photometrics (imaging following by computationally assisted feature extraction and measurement) promises to revolutionise biological research and agricultural production systems [1, 2, 3, 4, 5]. Automation of workflows remains a key challenge in the scaling of these approaches to cope with the requirements of large genetic experiments or, indeed, food production systems. Phenotyping aims to measure observable plant features, often as a response of environmental cues and/or variability between individuals. Traditionally, phenotyping has been a labour-intensive and costly process, usually manual and often destructive. High-throughput phenotyping technologies aim to address this problem by the use of non-destructive approaches either in glasshouses [1, 6, 2] or directly in the field [4, 7] integrating imaging, robotics, spectroscopy, high tech sensors and high-performance computing [8, 3].

Imaging has the potential to generate an enormous volume of data in real time, while image analysis to extract useful information is currently the main bottleneck. The ex-

Compiled on: November 18, 2019.
Draft manuscript prepared by the author.

traction of quantitative traits relies on the development and use of improved software techniques. Machine learning tools have been used to identify patterns in large biological datasets [8, 9, 10, 11, 12]. Recently, deep learning tools have been applied to accurately extract features from plant images [13, 14, 15, 16, 17, 18, 19, 20, 21].

Model organisms have been widely used to dissect different biological processes and provide a useful means to test and develop new methods that can subsequently be more widely applied to crop and ecological scenarios. *Arabidopsis thaliana* is a small, flowering plant widely used to address questions related to plant genetics, molecular, evolution, ecology, physiology, among others [22, 23, 24]. The seedling produces a small rosette that increases in size by addition of leaves. The central meristem produces an inflorescence that produces flowers and then fruits. The fruits are also known as pods or siliques [24]. The measurement of traits, such as growth rate, flowering and fruit number are key to evaluate plant performance and reproductive fitness [25]. However, many high-throughput imaging studies focus on growth dynamics of the rosette [26, 27, 9, 28], despite the importance of fruit production in reproductive and evolutionary processes [2, 29, 30, 31].

This work demonstrates that deep learning can be used to estimate fruit number from images. In particular, we have developed DeepPod, a framework for *Arabidopsis* silique detection that involves a deep neural network for patch based classification and an object reconstructor for silique localisation and counting. The framework has been validated using a separate dataset of 2408 images from biological experiments. This allowed the analysis of large numbers of plants inflorescences in an accurate and effective way providing a cost effective alternative to manual counting.

Background

Convolutional Neural Networks (CNNs) have become the dominant type of models for image classification [32]. The input for a CNN, typically an image, can be represented as a three-dimensional array of height$\times$width$\times$channels. A CNN contains convolutional layers, where inputs are passed through various filters for extracting features that are arranged as feature maps, prior to using the fully connected layers for classification or regression. The weights or parameters of the filters are shared among the neurons of the convolutional layers [33], not only to encourage detection of repeated patterns in the image but also to reduce the number of parameters for the network to learn. Other types of layers such as pooling are also often used in combination with convolutional layers to reduce the dimensionality of feature maps. A CNN can be trained using a back-propagation algorithm to update the weights in an iterative process, in order to minimise the loss function that measures the discrepancy between the predicted output and actual output for the training examples. What makes CNNs particularly attractive in computer vision is that they can directly extract features from images without the need for time-consuming, hand-crafted pre-processing or feature extraction steps, unlike classical machine learning approaches [34].

Recent publications have reported the application of deep learning in various plant phenotyping tasks such as leaf counting, age estimation, mutant classification, disease detection, fruit classification and plant organ localisation [14, 20, 19, 21, 18, 16, 15, 13]. Mohanty *et al.* [14] trained deep convolutional neural networks to identify 14 crop species and 26 diseases using a publicly available plant disease dataset. They built models with architectures of AlexNet [35] and GoogleNet [36] using transfer learning. Wang *et al.* [20] employed CNNs to establish disease severity in apple black rot images. Deep learning meta-architectures have also been considered for more complex scenarios. Fuentes *et al.* [19] demonstrated a combination of CNNs and deep feature extractors to recognise different diseases and pests in tomatoes, which dealt with inter- and intra-class variations. Deep learning was also used for cassava disease detection via mobile devices [21]. Pawara *et al.* [18] applied CNNs to classify leaf, fruits and flowers in field images. They compared the performance of classical classifiers to CNNs, where architectures such as GoogleNet and AlexNet gave the best results in the plant-related datasets used. Taghavi *et al.* [16] proposed a CNN-LSTM (-Long Short Term Memory) framework for plant classification using temporal sequence of images. Particularly the model features were learned using CNNs and the plant growth variation over time were modeled with LSTMs. Ubbens *et al.* [15] used CNNs for regression to perform leaf counting. They used rendered images of synthetic plants to augment an *Arabidopsis* rosette dataset and concluded that the augmentation with high-quality 3D synthetic plants improved the performance of leaf counting while real and synthetic plants could be interchangeably used for training a neural network. Pound *et al.* [13] demonstrated wheat root and shoot feature identification and localisation using two different standard CNN architectures for patch classification. For shoot features, they found that the leaf tips represented the hardest classification problem compared to the leaf base due to the existing variations in orientation, size, shape and colour of tips in their dataset. Further reconstruction from the classification results of the overlapping patches allows localisation of separate structural regions such as leaf tips and bases. However the objects of interest as a whole (such as leaves) are yet to be identified in order to extract more morphological features (e.g. leaf length and shape)

Our proposed framework treats the silique (or pod) counting problem as an object detection and segmentation problem followed by counting. One popular approach of deep learning frameworks for object detection is to train a single convolutional neural network to jointly perform object classification and localisation tasks, where the object localisations are usually defined by bounding boxes. Examples of such networks include Fast-RCNN (Regional-CNN), SSD (Single Shot Multibox Detector), YOLO (You Only Look Once) [37]. However training of such networks require labelled data with detailed segmentation or bounding boxes of individual objects, which are obtained usually through a very tedious manual process. Moreover, the image size allowed for the network input is limited due to the complexity of network architecture and the available memory.

In our case, the resolution of the raw images needs to be sufficiently high in order to preserve details of pods that are small and narrow, often overlapping. A single image can also contain a wide variation in the number of fruits from 0 to near 400, which poses further challenges for deep learning models when the available labelled data is limited.

To address these issues, we adopted an alternative approach that performs patch based classification and localisation in two separate phases. The first step is to classify a region of a suitable size in the original image into different parts of the inflorescences. In the localisation phase, each original image will be scanned and each patch classified as silique/not silique (i.e. as one of the four classes including the tip, base or body of siliques, and the stem inflorescence). Given an accurate classification of patches as silique/not silique, one could then estimate the number of siliques and their lengths to a good precision. The manual annotation task for the proposed framework was simple, involving collection of sufficient pixels from different defined structural parts of the plant.

Table 1. Information about the dedicated data for different tasks.

Dataset name	Number of images	Provided annotation	Used task
Set-1	144	Silique main structural elements, Silique count	Developing classification model, Developing counting pipeline
Set-2	2,408	Silique count	Evaluating counting pipeline

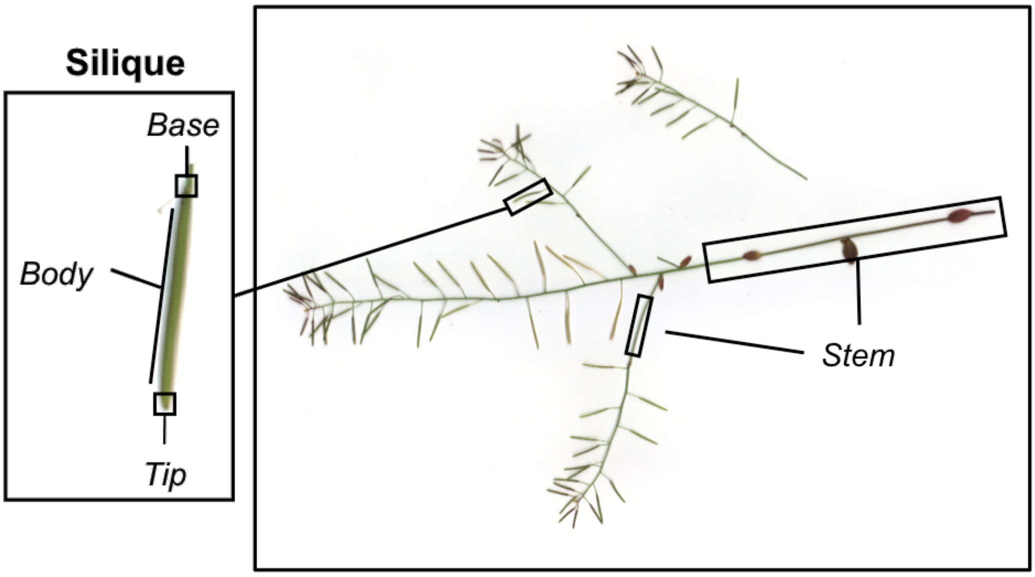

Figure 1. An illustrative example of image and features (the important parts of the plants) annotated for patch based classification..

Data Acquisition

A set of 2,552 images of mature inflorescences taken from a subset of the Multiparent Advanced Generation Inter-Cross (MAGIC) RIL population [38] were used to establish and test the CNN pipeline. A subset (referred to as Set-1 = 144 images) of this dataset was randomly selected for manual annotation and then used to train one shallow and one deep convolutional neural network. A total of 2,408 images (referred to as Set-2) were used to test the performance of the selected model. Information about the dedicated data for different tasks is stated in Table 1.

Plants were grown on an automatic watering platform within the National Plant Phenomics Centre (NPPC) (Aberystwyth University, United Kingdom) in 6cm diameter pots half filled with vermiculite and the upper half with 30% grit/sand: 70% Levington F1 (peat based compost). The vermiculite was used to restrict plant growth. Pots were filled to a uniform weight. Each plant was automatically weighed and irrigated from above to a 75% gravimetric water content daily.

The mature inflorescence or stem of each plant, with attached fruits was harvest and imaged in a flatbed scanner (Plustek, OpticPro A320). Images were saved at 300 dpi and stored in .PNG format with image size equal to 3600$\times$5100. The image file name includes the identification number for the line (e.g. ATxxx_001xxx represents RIL001) according to Kover *et al.* [38]. A sample image is shown in Figure 1. Manual counting of viable fruits in images was undertaken by a single person to minimize operator variation. ImageJ [39] was used to track the counting by setting a label to each fruit as it was counted.

Patch based Classification using CNNs

Data Preparation for Model Development

Data Annotation

An annotation tool with a graphical user interface (GUI) was built (in MatLab) to assist with manual annotation of different parts of the inflorescence. Figure 2 shows the schematic of this GUI with some screenshots of annotation. The user selects the class type (tip, base, body of the silique and stem) and clicks on the respective parts on each input image. The annotated parts (points clicked) were saved as defined locations based on image coordinates. An example annotated image illustrating the predefined parts of the silique (tip, base, body and stem) is given in Figure 3. This tool was used to manually annotate Set-1, which was used to develop the patch classifiers (see Section Patch Based Classification Problem).

The main advantages of this annotation platform include its relatively low cost and ease of use. Compared to other annotation approaches that require detailed segmentation, polygons or bounding boxes, this approach requires annotation of just four main structural elements. Using this platform, Set-1 was manually annotated by a single person in a total of 36 working hours.

Table 2 shows the number of annotations performed per class (before augmentation). This dataset was used in the initial training step for classifying whole inflorescence into defined parts. In order to prepare patches for classification, Set-1 was randomly split into training, validation and test sets as rounded of 65%, 20% and 15% of the 144 images.

Patch Generation & Augmentation

Similar approach to what was proposed in [13] has been followed for image patch generation and augmentation. Using the annotated data to prepare training samples, square bounding box patches were extracted while being centred at the manually annotated points. Subsequently, data augmentation [40, 41] was performed to increase the amount of training data via specific transformations as well as considering frames different from the centred ones. The patches of size 50 $\times$ 50 were first

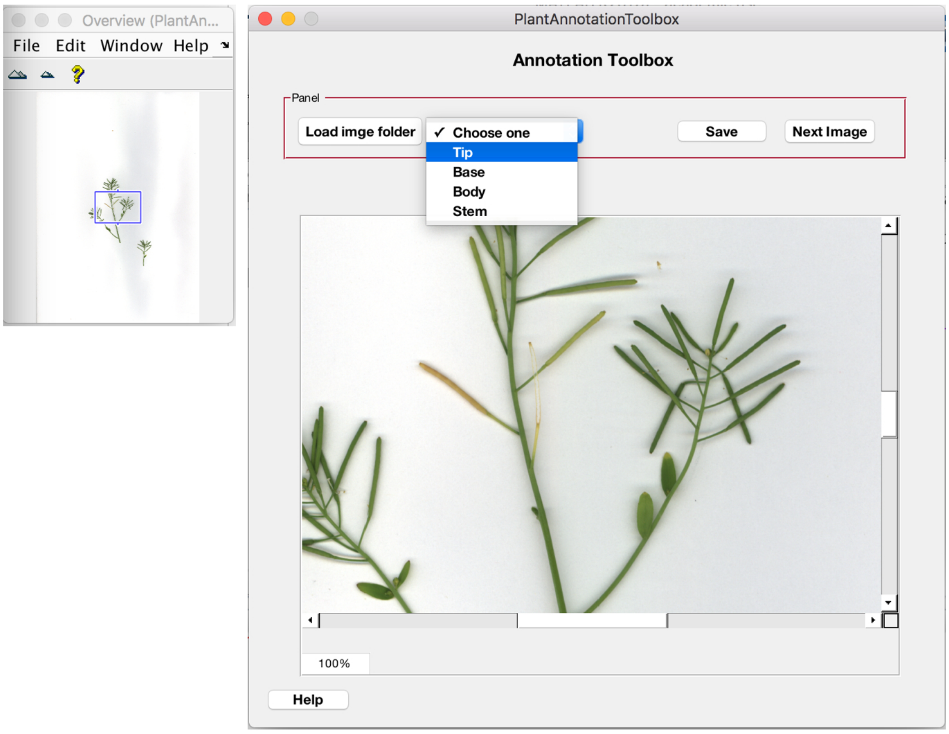

Figure 2. The developed GUI used for manually annotating plant parts.

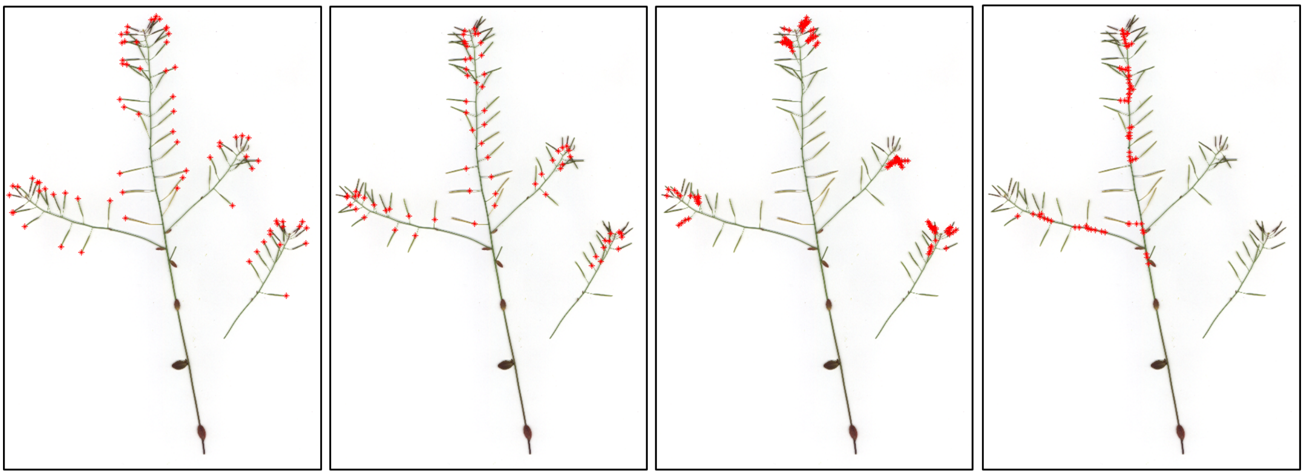

Figure 3. Example annotated images (from left to right) for tip, base, body and stem.

Table 2. Summary statistics for data annotation performed on Set-1.

Feature	Number of manual annotations
Silique Tip	7299
Silique Base	8058
Silique Body	11187
Stem	10266

extracted. Then, random 32×32 pixel crops followed by random mirroring or rotation were performed. For pre-processing, we normalised the data using the channel means and standard deviations on the training set. For validation samples, no augmentation was undertaken and the 32 × 32 patches centred at the annotated points were extracted. Figure 4 shows various examples of each class that were used in the training procedure.

Data Preparation for Testing

The training patches were centred at the annotated points followed by augmentation, as described earlier. To prepare test samples, the difference in the pixel intensity distribution between the testing data and the training/ validation data (that were used during training time) was taken into account. First, the whole image was scanned over with a sliding window and tiled into 32 × 32 patches with 50% overlap both in the vertical and horizontal direction (see Figure 7). Most pixels within the area of interest (plant area) would hence be included in four different patches. The patches belonging to the white background (lacking plant pixels) were excluded by thresholding.

The rationale behind selecting overlapping regions was (1) to increase the number of patches by a factor of four compared to without overlapping; (2) to make the patch classification

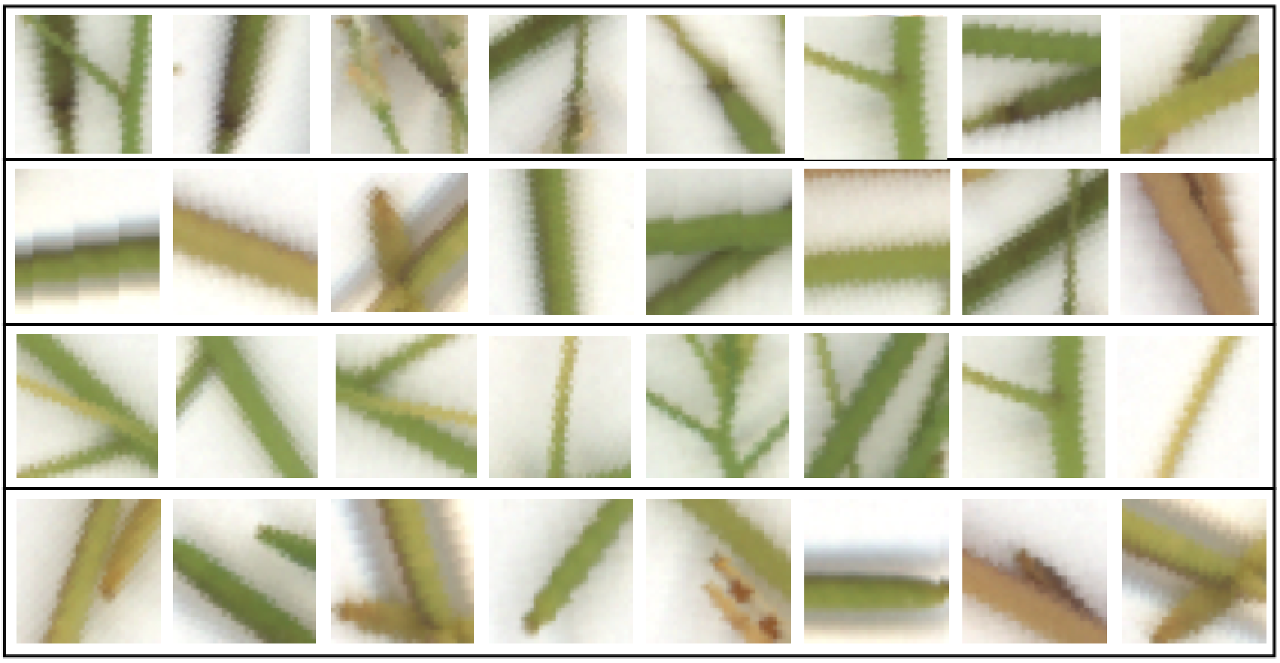

Figure 4. Example extracted patches using manual annotations. From top to bottom: samples of base, body, stem and tip, respectively. Note that, the key structural elements are not always centered in the patches due to the random cropping process for patch extraction.

more robust by combining multiple predictions.

When applying the model to test data, the difference between the sample distribution for training and that for testing, i.e. presence of potential covariate and dataset shift, can adversely affect the model generalisation performance. To address this issue, each test image will also be normalised using the channel wise mean and standard deviation of the training set.

Then the resultant patches were fed to the trained networks and the classification outcomes for each sample patch (tip, base, stem, body) were computed.

Building CNN Classifiers

In the next step, CNN-based classifiers were built to take extracted patches of interest as input, and to output probability scores for different labels $\{0, 1, 2, 3\}$ indicating the probability that the input patch contains a base, body, stem and tip, respectively.

Network Architecture

LeNet is a pioneering convolutional network that was proposed to classify handwriting digits [42]. LeNet architecture [43], consists of two sets of convolutional and pooling layers stacked on top of each other, followed by two fully connected layers and finally ending with a Softmax layer (see Figure 5). LeNet is a simple shallow network and has been chosen as a baseline model in this study, considering the potentially higher computational resource needs for running more complex deep learning models.

DenseNet is a model notable for its key characteristic of bypassing signals from preceding layers to subsequent layers that enforces optimal information flow in the form of feature maps. Amongst DenseNet variants [44], DenseNet-Basic is a successful model proposed for the CIFAR10 [34] image classification challenge. Hereafter, DenseNet-Basic will be referred to as "DenseNet". A simple DenseNet is made up of a total of L layers, while each layer is responsible for implementing a specific non-linear transformation, which is a composite function of different operations such as Batch Normalisation, Rectified Linear Unit, Pooling and Convolution [42, 44]. Within a dense block that consists of multiple densely connected layers with such composite functions, all layers are directly connected to each other, and each layer receives inputs (i.e. feature maps) from all preceding layers (as illustrated in the middle row of Figure 6). The number of feature maps generated from the composite function layer is usually fixed and is called the growth rate (k) for the DenseNet.

To facilitate down-sampling for CNNs, the network used for our experiment consisted of multiple dense blocks. And the dense blocks were connected to each other through transition layers (composed of a batch normalisation layer, a 1×1 convolutional layer, dropout layer and a 2×2 average pooling layer as shown in the bottom row of Figure 6).

The growth rate (k) was set to 12 for all dense blocks in order to generate narrow layers within the overall DenseNet structure (i.e. 3 dense-blocks with equal number of layers and 2 transition layers). A relatively small growth rate (of 12) was found to be sufficient to obtain satisfying results on our target datasets. The initial convolution layer incorporated 16 convolutions of size 3×3 on the input images. The number of feature-maps in all other layers follow the setting for k.

At the end of the last dense block (3rd dense block), a global average pooling was performed to minimize over-fitting by reducing the total number of parameters in the model. The final Softmax classifier of four output nodes will predict the probability for each class based on the extracted features in the network. The rest of the model's parameters with regards to the kernel, stride and padding sizes were kept as default as detailed in [44].

Training

In our experiments with LeNet and DenseNet, similar configuration has been applied as in Huang et al. [44]. Both models were trained via a stochastic gradient descent solver with the parameters set to Gamma = 0.1 (for the learning rate decreasing factor), momentum = 0.9 (for weight update from the previous iteration) and the weight-decay factor = 10^{-5}. We

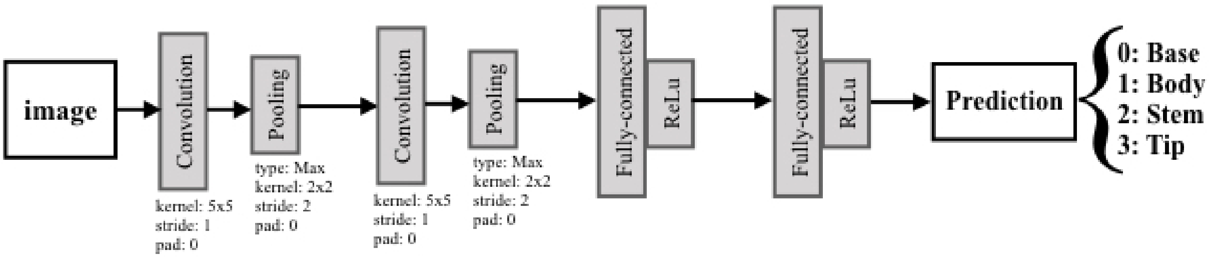

Figure 5. LeNet architecture.

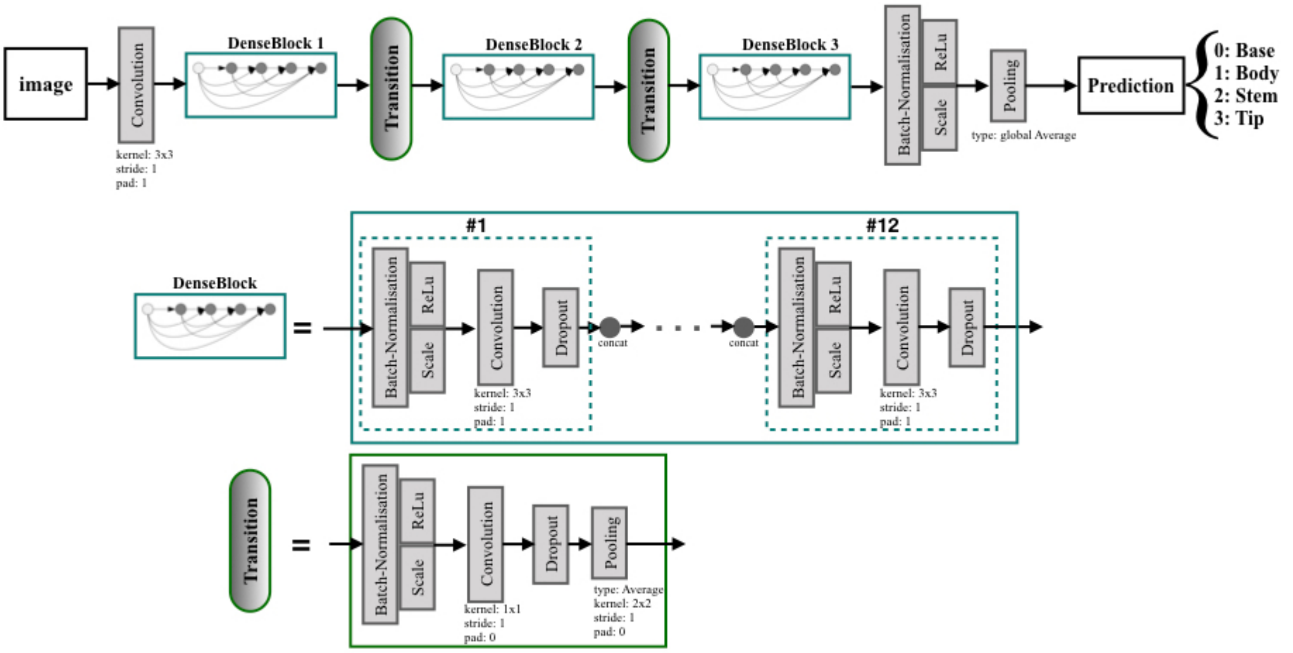

Figure 6. The DenseNet-Basic architecture used for patch based *Arabidopsis* structural part classification. The feature-map sizes in the three dense-blocks were 32 × 32, 16 × 16, and 8 × 8, respectively.

Table 3. Classification results on the validation samples.

	LeNet	DenseNet
Accuracy	80.55%	86.80%
Loss	0.64	0.37

trained LeNet and DenseNet with mini-batches of size 64 and 8 (according to our hardware specifications), respectively. Both models were trained using an initial learning rate of 0.001 with 33% step down policy. LeNet was trained for 15 epochs and DenseNet was trained for 30 epochs. In our implementation, the LeNet and DenseNet models pretrained on the CIFAR10 dataset [34] were used to initialise the weights, whilst the networks were fine-tuned using prepared training data from the silique dataset. In the pre-processing step for each model, the mean patch calculated on the training set patches was subtracted for each sample patch being fed.

All CNN training and testing was performed within the Caffe framework [45]. The computations were carried out using a NVIDIA GeForce GTX 1080 GPU, Intel Core i7-4790 Processor and Ubuntu 16.04 operating system.

Table 3 shows the classification accuracy and loss for both networks on the validation data from Set-1 after training.

Performance on Patch Based Classification

In the initial evaluation, we used the test samples in our model development data Set-1 to evaluate the classification and detection performance of both the shallow and deep networks. The aim of this comparative evaluation was to choose the best model for correct classification of patches and estimating silique counts on the smaller development dataset.

The classification results of both networks are presented in Tables 4 and 5 in terms of a confusion matrix, per-class precision and recall, and total classification precision and recall. Note that only annotated patches have been considered for this evaluation. The DenseNet network has higher representational power due to its deeper architecture and its use of features of multiple levels for classification in comparison to the LeNet network; its efficacy in the learning task has also been evidenced by its higher accuracy in classifying plant parts (as shown in Tables 4 and 5).

Post-processing for Silique Localisation & Counting

Table 4. Performance of patch based classification on the testing images for model development using LeNet network.

Actual \ Predicted	Base	Body	Stem	Tip	Precision (%)	Recall (%)
Base	344	12	52	4	74.1	83.5
Body	15	280	26	30	79.5	79.8
Stem	14	29	270	4	75.8	85.2
Tip	91	31	8	169	81.6	56.5
Total Precision = 77.8 %, Total Recall = 76.2 %						

Table 5. Performance of patch based classification on the testing images for model development using DenseNet network.

Actual \ Predicted	Base	Body	Stem	Tip	Precision (%)	Recall (%)
Base	392	4	14	2	93.6	95.1
Body	15	290	13	33	93.2	82.6
Stem	11	14	290	2	91.5	91.5
Tip	1	3	0	295	88.9	98.7
Total Precision = 91.8 %, Total Recall = 92 %						

Image Reconstruction

Given the classification of various patches in an image, post processing can be applied to reconstruct the image and detect probable silique appearances. The plant regions are first identified from the background (including borders) using simple thresholding methods. Then the plant regions are further segmented into four classes based on labelling of the patches of interest.

As the patches for a test image are generated with 50% overlap along both the horizontal and vertical direction, each patch consists of four squares of equal size (16×16), called sub-patches. Each sub-patch has four class predictions from four adjacent patches, the final decision is inferred through majority vote and the label for each pixel in the sub-patch was determined accordingly (See Figure 7). In case of a tied vote for several classes, the average probability of those classes for the image will be assigned to the sub-patch and its pixels.

Silique Counting

To count siliques in the reconstructed image, a silique is defined as an area composed of three interconnected parts: one tip, one body and one base in such a way that the body is located between the tip and the base (Figure 1). The areas where tips and bodies presenting shared borders are initially identified, these tip-body areas were extended through shared borders to search for the connected tips, which eventually established a combined area for a silique object.

In practice, many touching or overlapping siliques were observed in the captured images, which was a problem for detecting individual siliques accurately. In the case where one silique object area contained multiple tips or bases, the angle between the potentially overlaid siliques was calculated, using a cross product between the different vectors linking the bases to the corresponding tips. For example, for the case of two siliques overlaying (often with the same apparent base or tip), the centers of tips and bases were computed; then using a cross product, the centers were connected in order to calculate the angle between overlaid siliques. If the measured angle was larger than a predetermined threshold, the region was considered as two distinctive siliques, otherwise as a single silique. The value of the threshold was set to 0.05 *Radian* in our experiments according to the resolution of the images. Please see supplementary Figure S1 for an illustrative example on how to detect/count individual siliques with overlapping regions.

Test results for Silique Counting

Results on the test data for model development

Figure 8 shows the results of image reconstruction for several randomly selected images after patch classification (using the DenseNet network), with colours indicating different structural parts of the plant.

Table 6 reports the performance of silique count prediction using the two different trained networks. In this table, the correlation coefficient (for the linear relationship between the prediction and the manual counts) shows that the prediction using the deeper model (DenseNet) is more accurate than using the shallower model (LeNet). This linear correlation can be better seen in Figure 9 showing the scatter plots of the actual vs automated silique counts. We also examined the distribution of the errors (actual−prediction) in silique counting, see Figure 10 for the histograms of errors for the two trained models. It appears that both LeNet and DenseNet under estimated the counts compared to manual counting in most cases.

Comparing a shallow and a deep network for classifying image patches, we concluded that the classification results and the quality of the count estimation show improvement from using the deeper architecture. Therefore, DenseNet has been selected for identifying siliques, as it appeared to be more robust to the variations in shape and size. This is probably in part a consequence of using a training set of images from diverse individuals harvested at different stages of silique maturation.

Results on the separate test data

To further evaluate the proposed framework, we used a separate large dataset of 2,408 images available within the NPPC. The scatter plot in Figure 11 shows a high positive correlation (Pearson correlation coefficient $R^2 = 0.90$) between the manual counts and automated counts. With the reconstructed silique objects, additional morphological features could be extracted including silique length. Predicted silique number and statistics for silique length (mean, maximum and minimum) per image are reported in Supplementary Data S1.

The CNN-based prediction tends to under-estimate compared to actual manual counting. To better understand where the problem lies, detailed detection results have also been visualised, see Figure 12 for some random examples. It seems that the current post processing method might have difficulty in detecting some small or overlapping siliques.

Discussion

A recent computer vision approach to fruit number estimation involves linear regression using selected skeleton descriptors (such as junction numbers and number of triple points) extracted after segmentation and 2D skeletonisation, resulting in a validation correlation of $R^2 = 0.91$ between observed and automated values for the best performing model on the 100 examples [2] in a development dataset. When applied on the dataset from a separate experiment, although the model can qualitatively capture the main phenotype under investigation, its accuracy against the manual counts dropped significantly to R^2 of 0.7. This suggests that this regression approach to fruit counting might not be generalised to other conditions as

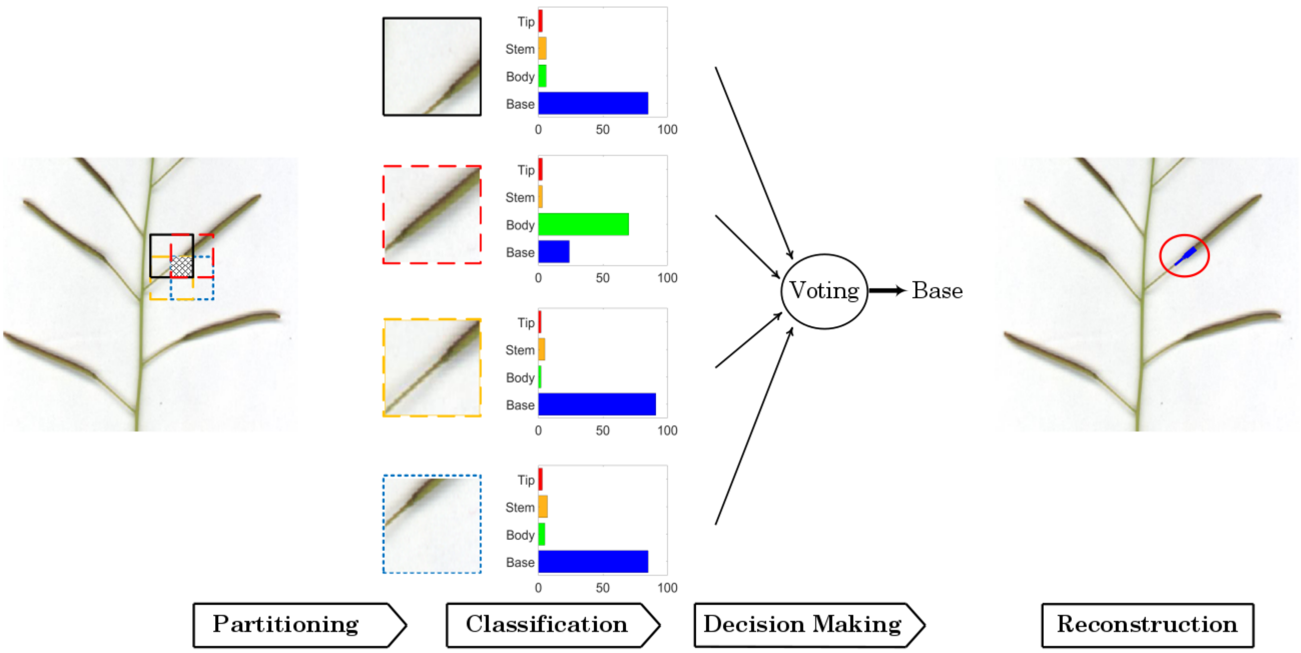

Figure 7. Flowchart of the sub-patch labelling step for image reconstruction.

Table 6. Performance for silique count prediction compared to manual counting on the 22 test images for model development.

Metric	LeNet	DenseNet
Correlation coefficient	0.932	0.954
Root mean squared error	20.35	12.45

effectively as our object recognition approach. Apparently this non-deep learning approach used only "handcrafted" global features with resulting models more specific to the conditions for training, whereas our approach used both local features (for patch classification) and some more global features (for object reconstruction).

Based on our test results on silique counting, we expect our method to be useful for species with similar fruit morphology such as Canola (Oilseed Rape) and other brassicas. However, the CNN will most likely need to be fine-tuned for diverse silique morphology and imaging conditions.

There are several promising directions for future work for which the developed software can be improved such as the detection of other traits like silique length or branch number. These two traits have been reported to be a good proxy of seed number and therefore could be important for estimating productivity [46]. The following considerations should be taken into account in future to improve the classification and detection performance:

i. The robustness of the representations in both networks relied largely on the quality and quantity of the training and test data. Increased variety in the training samples (along with artificial augmentation) should provide more robust learned representations and may facilitate extension to other species.
ii. Deep learning models can take the whole image or the patches as input. In this study, a patch based classifier was used and the image was scanned over with a sliding window, classifying the patches. However, feeding all patches to the network was time-consuming and the designated patch overlap produces substantial redundancy. To overcome these issues, deep neural networks taking the whole image as input for object detection can be explored.
iii. Generative adversarial networks (GANs) [47] have been widely used in segmentation problems on real world [48, 49] and medical data (see our recent application of these models on medical images [50, 51]). To avoid the need for post-processing (which affects the performance), different types of GANs should be investigated.
iv. DeepPod can be used to accelerate the development of even more robust fruit recognition approaches. DeepPod can rapidly provide more annotated images as the output of the proposed DenseNet model can be used to automatically generate detailed fruit annotation suggestions. A human annotator would then focus on correcting false negatives (by adding missed siliques) and false positives (or removing falsely detected ones) instead of spending so much time on marking each fruit contour individually.

Conclusion

In summary, we have developed DeepPod, an image-based deep learning framework for fruit counting. We have demonstrated DeepPod's effectiveness in silique detection and counting for *Arabidopsis*, as well as challenges due to presence of overlapping siliques and variability in fruit morphology. The pipeline developed has been shown to be cost effective in image annotation for model development. To further improve the pipeline, more robust and scale invariant methods will be investigated for object detection and for extraction of more morphological traits. Additionally active learning and transfer learning could be applied for more effective data annotation and machine learning modelling.

Availability of source code and requirements

- Project name: DeepPod

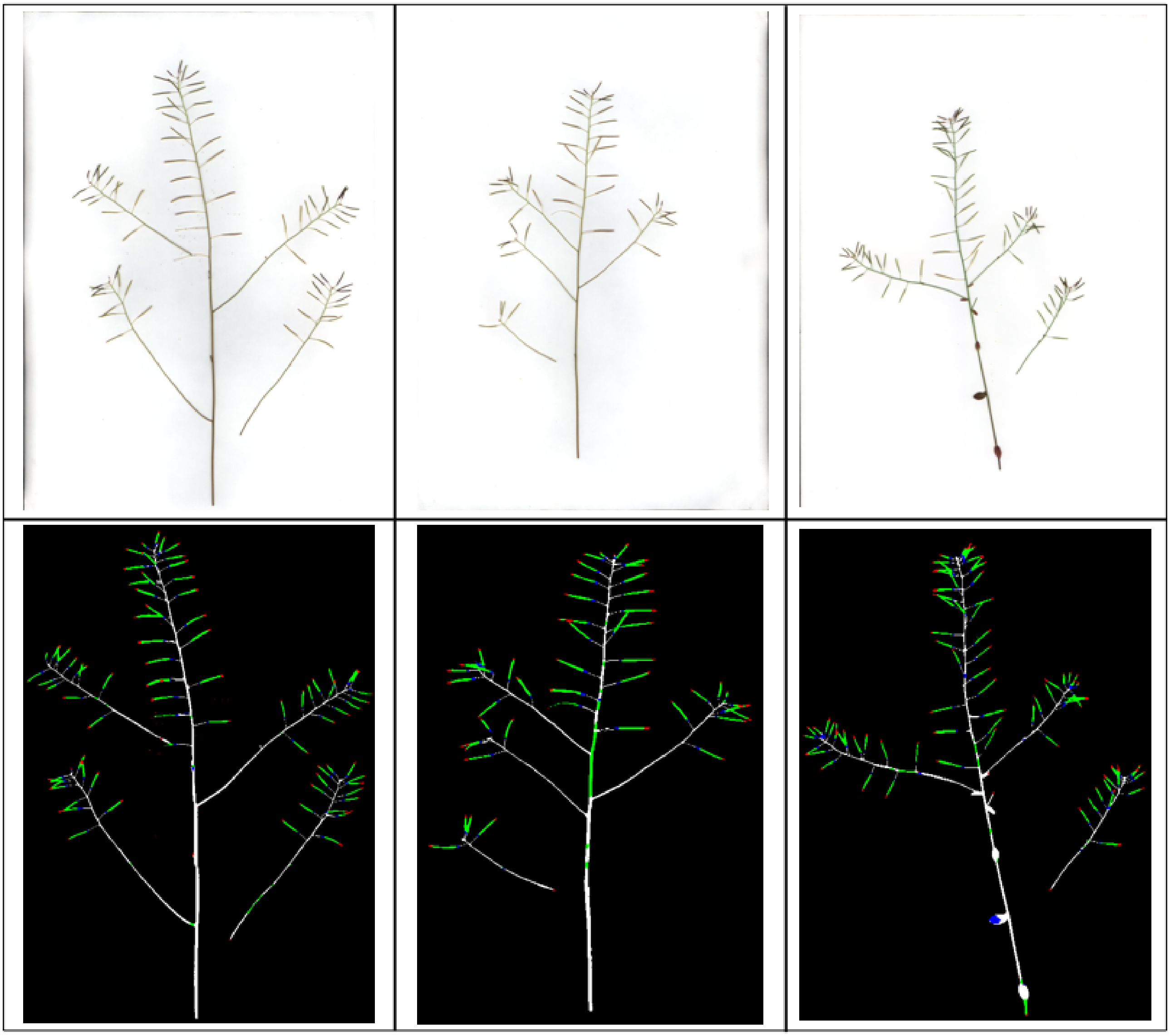

Figure 8. Three example results of labelling on the reconstructed plant images based the DenseNet patch based classification. Tips, bodies, bases and stems are indicated in red, green, blue and white, respectively.

- Project home page: https://github.com/AzmHmd/DeepPod.git
- Operating system(s): Platform independent
- Programming language: MATLAB
- Other requirements: CUDA version: 8.0, CuDNN version: v5.1, BLAS: atlas, CAFFE version: 1.0.0-rc3, DIGITS version: 5.1-dev, MATLAB version: 9.3 or above
- License: MIT
- The annotation toolbox (also included in DeepPod project) has been registered in the www.SciCrunch.org data sharing and display platform with the Research Resource Identification Initiative ID (RRID) number of *"SCR_017413"*, under the name of *"Plant Phenotyping Annotation Toolbox"* .

Availability of supporting data and materials

The dataset for model development (Set-1, including 144 raw images and their annotations, and manual silique counts), and the dataset for testing (Set-2, including 2,408 raw images and their manual silique counts), are available in the Aberystwyth research data repository, DOI:10.20391/21154739-f718-457b-96ff-838408f2b696.

Additional files

- Supplementary Figure S1: an illustrative example on identification of individual siliques with overlapping regions
- Supplementary Data S1: a CSV file reporting the predicted silique count, mean and range of silique length (in pixels) for each image in Set-2.

Declarations

List of abbreviations

CNN	Convolutional Neural Network
GUI	Graphical User Interface
LSTM	Long Short Term Memory
NPPC	National Plant Phenomic Centre
RCNN	Regional CNN
SSD	Single Shot Multibox Detector
YOLO	You Only Look Once

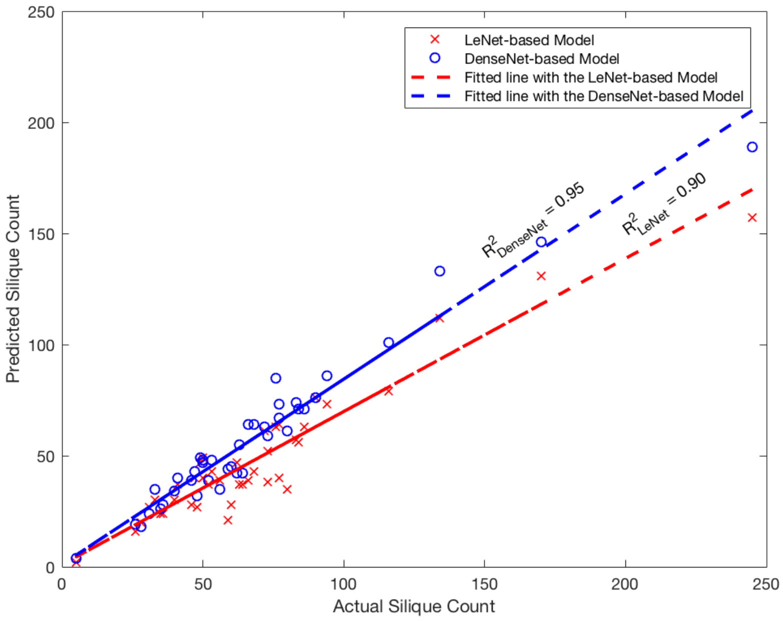

Figure 9. Predicted counts using the two models using validation and testing samples. $R^2 = 0.90$ for the LeNet-based model and $R^2 = 0.95$ for the DenseNet-based model.

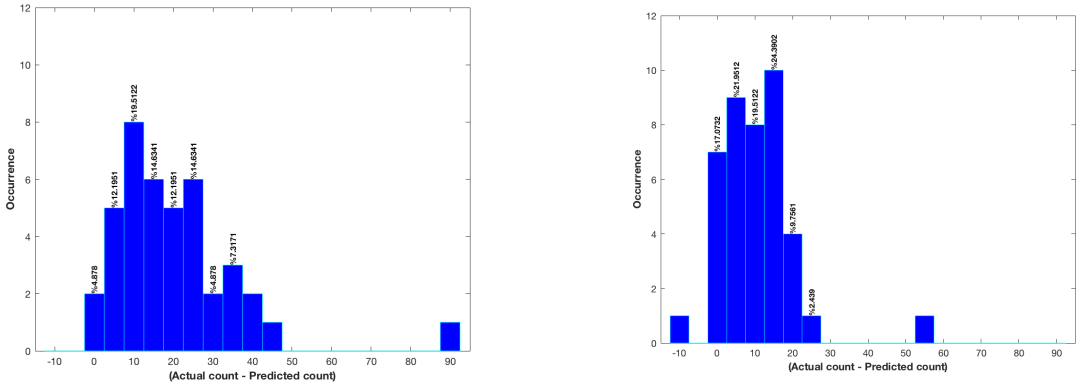

(a) Actual (manual) counts – LeNet based Prediction

(b) Actual (manual) counts – DenseNet based Prediction

Figure 10. The histograms of errors in silique count prediction for the LeNet and DenseNet models.

Consent for publication

Not applicable

Competing Interests

The authors declare that they have no competing interests

Author's Contributions

J.D., F.C. and C.L designed the study and provided the images. G.G.-M. performed manual counting and manual annotations. A.H. developed the annotation toolbox, performed the deep learning and data analysis, testing and evaluation tasks. A.H. and M.G. carried out the post processing analysis. AH and G.G.-M. drafted the manuscript. All the authors provided comments and corrected the manuscript.

Acknowledgements

The authors would like to gratefully acknowledge Sandy Spence and Alun Jones for their support and maintenance of the GPU and the systems used for this research. We acknowledge Dr Jay Biernaskie from University of Oxford for allowing us to use previously unpublished image dataset (Set-1 and Set-2). We also acknowledge the team of the National Plant Phenomics Centre, mainly to Lina Avila Clasen for her help in acquiring the images and manual counting. GG-M acknowledges receipt of a AberDoc scholarship from Aberystwyth University; JHD, FC and CL funding from BBSRC (grants BB/CAP1730/1, BB/P013376/1 and BB/P003095/1).

References

1. Chen D, Neumann K, Friedel S, Kilian B, Chen M, Altmann T, et al. Dissecting the phenotypic components of crop plant growth and drought responses based

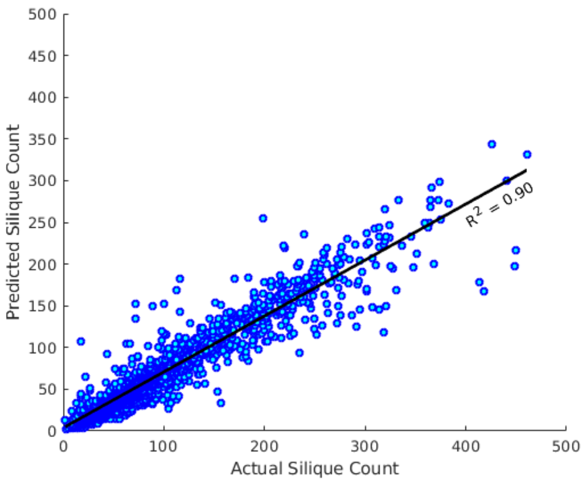

Figure 11. Predicted silique count and manual counting from Set-2 testing samples including 2,408 images. R^2 = 0.90

on high-throughput image analysis. The Plant Cell 2014;26(12):4636–4655.

2. Vasseur F, Bresson J, Wang G, Schwab R, Weigel D. Image-based methods for phenotyping growth dynamics and fitness components in Arabidopsis thaliana. Plant Methods 2018;14(1):63.
3. Furbank RT, Tester M. Phenomics–technologies to relieve the phenotyping bottleneck. Trends in Plant Science 2011;16(12):635–644.
4. Pauli D, Andrade-Sanchez P, Carmo-Silva AE, Gazave E, French AN, Heun J, et al. Field-based high-throughput plant phenotyping reveals the temporal patterns of quantitative trait loci associated with stress-responsive traits in cotton. G3: Genes, Genomes, Genetics 2016;6(4):865–879.
5. Shakoor N, Lee S, Mockler TC. High throughput phenotyping to accelerate crop breeding and monitoring of diseases in the field. Current Opinion in Plant Biology 2017;38:184–192.
6. Camargo AV, Mott R, Gardner KA, Mackay IJ, Corke F, Doonan JH, et al. Determining phenological patterns associated with the onset of senescence in a wheat MAGIC mapping population. Frontiers in Plant Science 2016;7:1540.
7. Liebisch F, Kirchgessner N, Schneider D, Walter A, Hund A. Remote, aerial phenotyping of maize traits with a mobile multi-sensor approach. Plant Methods 2015;11(1):9.
8. Singh A, Ganapathysubramanian B, Singh AK, Sarkar S. Machine learning for high-throughput stress phenotyping in plants. Trends in Plant Science 2016;21(2):110–124.
9. Pape JM, Klukas C. Utilizing machine learning approaches to improve the prediction of leaf counts and individual leaf segmentation of rosette plant images. Proceedings of the Computer Vision Problems in Plant Phenotyping (CVPPP) 2015;p. 1–12.
10. Naik HS, Zhang J, Lofquist A, Assefa T, Sarkar S, Ackerman D, et al. A real-time phenotyping framework using machine learning for plant stress severity rating in soybean. Plant Methods 2017;13(1):23.
11. Atkinson JA, Lobet G, Noll M, Meyer PE, Griffiths M, Wells DM. Combining semi-automated image analysis techniques with machine learning algorithms to accelerate large scale genetic studies. GigaScience 2017;.
12. Arinkin V, Digel I, Porst D, Artmann AT, Artmann GM. Phenotyping date palm varieties via leaflet cross-sectional imaging and artificial neural network application. BMC Bioinformatics 2014;15(1):55.

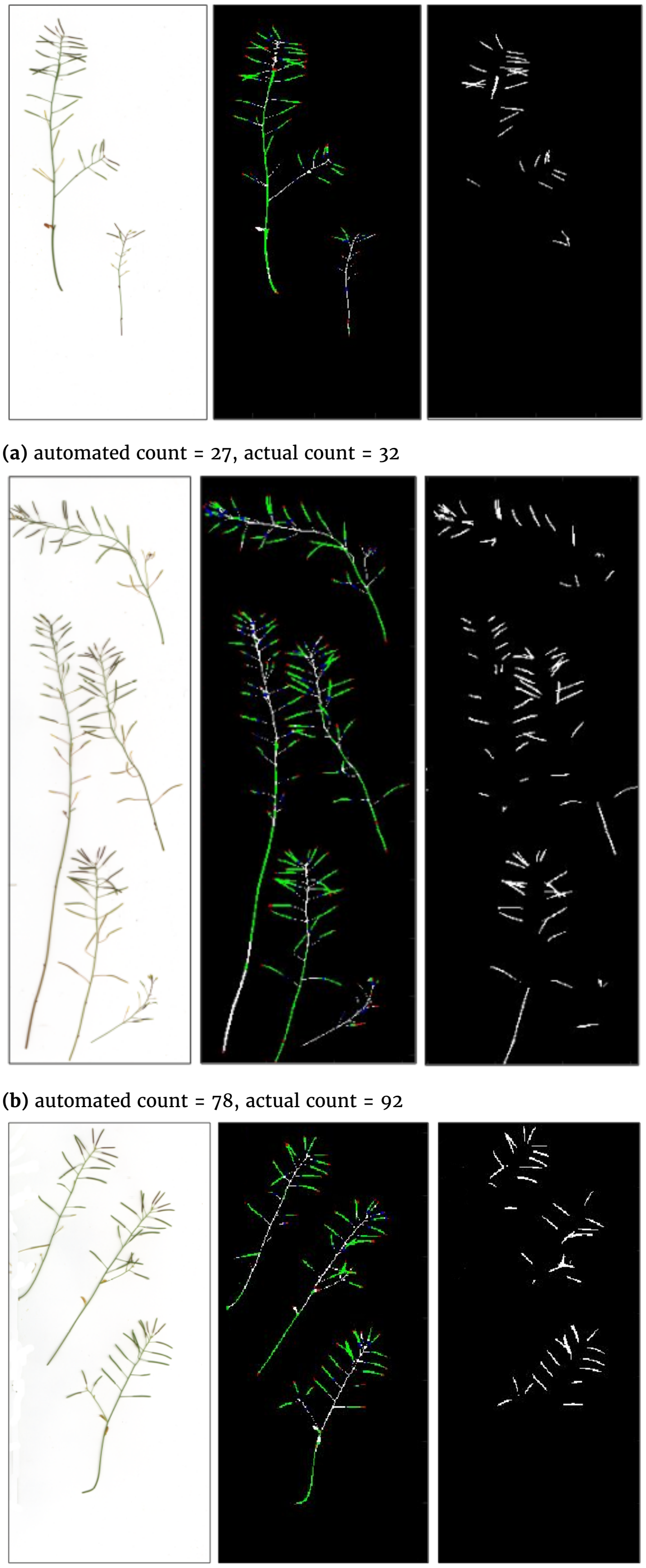

(a) automated count = 27, actual count = 32

(b) automated count = 78, actual count = 92

(c) automated count = 40, actual count = 52

Figure 12. Results of the DenseNet framework applied to some random samples from the larger testing dataset. From left to right: original plant images, sub-patch labelling and image reconstruction (Tips, bodies, bases and stems are indicated in red, green, blue and white, respectively), silique region detection (in white).

13. Pound MP, Atkinson JA, Townsend AJ, Wilson MH, Griffiths M, Jackson AS, et al. Deep machine learning provides state-of-the-art performance in image-based plant phe-

notyping. Gigascience 2017;6(10):gix083.
14. Mohanty SP, Hughes DP, Salathé M. Using deep learning for image-based plant disease detection. Frontiers in Plant Science 2016;7.
15. Ubbens J, Cieslak M, Prusinkiewicz P, Stavness I. The use of plant models in deep learning: an application to leaf counting in rosette plants. Plant Methods 2018;14(1):6.
16. Namin ST, Esmaeilzadeh M, Najafi M, Brown TB, Borevitz JO. Deep phenotyping: deep learning for temporal phenotype/genotype classification. Plant Methods 2018;14(1):66.
17. Ubbens JR, Stavness I. Deep plant phenomics: a deep learning platform for complex plant phenotyping tasks. Frontiers in Plant Science 2017;8:1190.
18. Pawara P, Okafor E, Surinta O, Schomaker L, Wiering M. Comparing Local Descriptors and Bags of Visual Words to Deep Convolutional Neural Networks for Plant Recognition. In: ICPRAM; 2017. p. 479–486.
19. Fuentes A, Yoon S, Kim SC, Park DS. A robust deep-learning-based detector for real-time tomato plant diseases and pests recognition. Sensors 2017;17(9):2022.
20. Wang G, Sun Y, Wang J. Automatic image-based plant disease severity estimation using deep learning. Computational Intelligence and Neuroscience 2017;2017.
21. Ramcharan A, Baranowski K, McCloskey P, Ahamed B, Legg J, Hughes D. Transfer Learning for Image-Based Cassava Disease Detection. Frontiers in Plant Science 2017;8:1852.
22. Mitchell-Olds T. Arabidopsis thaliana and its wild relatives: a model system for ecology and evolution. Trends in Ecology & Evolution 2001;16(12):693–700.
23. Koornneef M, Meinke D. The development of Arabidopsis as a model plant. The Plant Journal 2010;61(6):909–921.
24. Krämer U, Planting molecular functions in an ecological context with Arabidopsis thaliana. PubMed PMID: 25807084; PubMed Central PMCID: PMC4373673.; 2015.
25. Reboud X, Le Corre V, Scarcelli N, Roux F, David J, Bataillon T, et al. Natural variation among accessions of Arabidopsis thaliana: beyond the flowering date, what morphological traits are relevant to study adaptation. Plant Adaptation: Molecular Genetics and Ecology, edited by QCB Cronk, J Whitton, RH Ree and IEP Taylor NRC Research Press, Ottawa, Ontario, Canada 2004;p. 135–142.
26. Bac-Molenaar JA, Vreugdenhil D, Granier C, Keurentjes JJ. Genome-wide association mapping of growth dynamics detects time-specific and general quantitative trait loci. Journal of Experimental Botany 2015;66(18):5567–5580.
27. Bac-Molenaar JA, Granier C, Keurentjes JJ, Vreugdenhil D. Genome-wide association mapping of time-dependent growth responses to moderate drought stress in Arabidopsis. Plant, Cell & Environment 2016;39(1):88–102.
28. Minervini M, Abdelsamea MM, Tsaftaris SA. Image-based plant phenotyping with incremental learning and active contours. Ecological Informatics 2014;23:35–48.
29. Augustin M, Haxhimusa Y, Busch W, Kropatsch WG. A framework for the extraction of quantitative traits from 2D images of mature Arabidopsis thaliana. Machine Vision and Applications 2016;27(5):647–661.
30. Bush MS, Crowe N, Zheng T, Doonan JH. The RNA helicase, eIF 4A-1, is required for ovule development and cell size homeostasis in Arabidopsis. The Plant Journal 2015;84(5):989–1004.
31. Zheng T, Nibau C, Phillips DW, Jenkins G, Armstrong SJ, Doonan JH. CDKG1 protein kinase is essential for synapsis and male meiosis at high ambient temperature in Arabidopsis thaliana. Proceedings of the National Academy of SSciences 2014;111(6):2182–2187.
32. LeCun Y, Kavukcuoglu K, Farabet C. Convolutional networks and applications in vision. In: Proceedings of IEEE International Symposium on Circuits and Systems (ISCAS),; 2010. p. 253–256.
33. Simonyan K, Zisserman A. Very deep convolutional networks for large-scale image recognition. International Conference on Learning Representations, arXiv preprint arXiv:14091556 2014;.
34. Krizhevsky A, Hinton G. Learning multiple layers of features from tiny images. Technical Report, University of Toronto 2009;(4).
35. Krizhevsky A, Sutskever I, Hinton GE. Imagenet classification with deep convolutional neural networks. In: Advances in Neural Information Processing Systems; 2012. p. 1097–1105.
36. Szegedy C, Liu W, Jia Y, Sermanet P, Reed S, Anguelov D, et al. Going deeper with convolutions. In: Proceedings of the IEEE Conference on Computer Vision and Pattern Recognition; 2015. p. 1–9.
37. Redmon J, Divvala S, Girshick R, Farhadi A. You only look once: Unified, real-time object detection. In: Proceedings of the IEEE Conference on Computer Vision and Pattern Recognition; 2016. p. 779–788.
38. Kover PX, Valdar W, Trakalo J, Scarcelli N, Ehrenreich IM, Purugganan MD, et al. A multiparent advanced generation inter-cross to fine-map quantitative traits in Arabidopsis thaliana. PLoS genetics 2009;5(7):e1000551.
39. Abràmoff MD, Magalhães PJ, Ram SJ. Image processing with ImageJ. Biophotonics international 2004;11(7):36–42.
40. Zhang C, Bengio S, Hardt M, Recht B, Vinyals O. Understanding deep learning requires rethinking generalization. arXiv preprint arXiv:161103530 2016;.
41. Hamidinekoo A, Suhail Z, Qaiser T, Zwiggelaar R. Investigating the effect of various augmentations on the input data fed to a convolutional neural network for the task of mammographic mass classification. In: Annual Conference on Medical Image Understanding and Analysis Springer; 2017. p. 398–409.
42. LeCun Y, Bengio Y, Hinton G. Deep learning. Nature 2015;521(7553):436–444.
43. LeCun Y, Bottou L, Bengio Y, Haffner P. Gradient-based learning applied to document recognition. Proceedings of the IEEE 1998;86(11):2278–2324.
44. Huang G, Liu Z, van der Maaten L, Weinberger KQ. Densely connected convolutional networks. In: Proceedings of the IEEE Conference on Computer Vision and Pattern Recognition; 2017. .
45. Jia Y, Shelhamer E, Donahue J, Karayev S, Long J, Girshick R, et al. Caffe: Convolutional architecture for fast feature embedding. In: Proceedings of the 22nd ACM International Conference on Multimedia ACM; 2014. p. 675–678.
46. Bac-Molenaar JA, Fradin EF, Becker FF, Rienstra JA, van der Schoot J, Vreugdenhil D, et al. Genome-wide association mapping of fertility reduction upon heat stress reveals developmental stage-specific QTLs in Arabidopsis thaliana. The Plant Cell 2015;p. tpc–15.
47. Goodfellow I, Pouget-Abadie J, Mirza M, Xu B, Warde-Farley D, Ozair S, et al. Generative adversarial nets. In: Advances in Neural Information Processing Systems; 2014. p. 2672–2680.
48. Isola P, Zhu JY, Zhou T, Efros AA. Image-to-image translation with conditional adversarial networks. In: Proceedings of the IEEE Conference on Computer Vision and Pattern Recognition; 2017. p. 1125–1134.
49. Luc P, Couprie C, Chintala S, Verbeek J. Semantic segmentation using adversarial networks. arXiv preprint arXiv:161108408 2016;.
50. Hamidinekoo A, Denton E, Rampun A, Honnor K, Zwiggelaar R. Deep learning in mammography and breast histology, an overview and future trends. Medical Image Analysis 2018;47:45–67.

51. Grall A, Hamidinekoo A, Malcolm P, Zwiggelaar R. Using a conditional Generative Adversarial Network (cGAN) for Prostate Segmentation. In: 23rd Conference on Medical Image Understanding and Analysis; 2019. .

Supplementary Figure S1

Click here to access/download
Supplementary Material
SupplFigureS1.pdf

Supplementary Data S1

Click here to access/download
Supplementary Material
Supplementary_Data_S1.csv
