# Supplementary material for: DeepPod: a convolutional neural network based quantification of fruit number in Arabidopsis
Source: Gigascience. 2020 Mar 4;9(3):giaa012. doi: 10.1093/gigascience/giaa012 (PMC7055469; doi:10.1093/gigascience/giaa012)
Supplement: giaa012_Supplemental_Files [file giaa012_supplemental_files.zip › Supplementary_figures.pdf]

Supplementary Figures

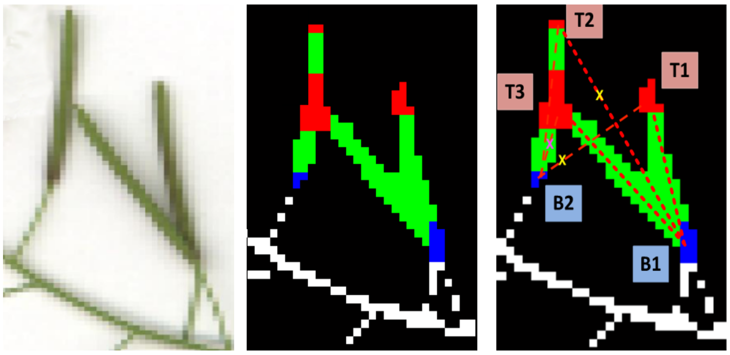

(a) (b) (c)

Figure S1: An illustrative example on identification of individual siliques with overlapping regions.

(a) A connected area (of base-body-tip) identified as a composite silique object with overlapping siliques; (b) the reconstructed results with labels of four structural elements (red: Tip, green: Body, blue: Base and white: Stem);(c) the procedure for detecting and counting siliques in the overlaying silique area.

Step1: Identify all the regions of bases {B1, B2} and tips {T1, T2, T3}.

Step2: For each of bases, e.g. B1, generate connection lines from the center of the base to the centers of all tips, {T1, T2, T3}, as potential line corresponding to a single silique object (in dotted lines).

Step3: Apply Silique Definition Rule to the lines created in Step2, i.e. if the majority (80%) of a line is laid in the body area, then that connection is considered as a potential silique (e.g. B1-T1, B1-T3), otherwise it is discarded (e.g. B1-T2).

Step4: Repeat Step2-3 for all other bases. E.g. connection line B2-T1 will be discarded after application of silique definition rule, and connection lines B2-T2 and B2-T3 will be kept as potential lines for siliques.

Step5: Iteratively remove possible duplicate silique lines for each pair of lines with shared regions (mostly shared bases or tips). Compute the angles between the overlaying lines (using cross-product between the two vectors). Discard one of the line if the measured angle is less than a predefined threshold (i.e. 0.05 radiance in this work). For each overlaying line pair, only one line (i.e. B2-T2) will be kept for silique counting due to the angle constraint. The remaining silique lines will then be B1-T3, B1-T1 and B2-T2, representing three different siliques.

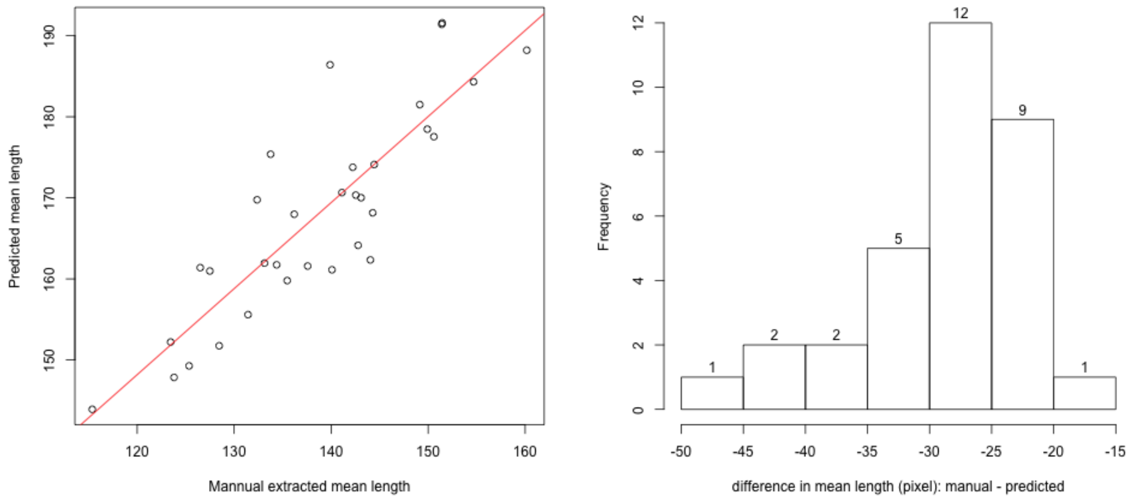

(a) (b)

Figure S2: Comparison of predicted mean silique length with manual estimate: (a) scatter plot, with a Pearson correlation of $R^2 = 0.746$. **; (b) the distribution of the mean length deviation (manual – predicted) in pixel.**

In order to validate the silique length prediction, we manually annotated 32 images randomly selected from Set-2 in more details (see Supplementary Data S2-S3). In total 2359 siliques were annotated with polylines (using an open source image annotator VIA 2.08, www.robots.ox.ac.uk/~vgg/software/via/).

The length of each silique has been estimated using the measure of the polyline length. In this preliminary work for extracting morphological features, the mean of the predicted silique length for each image was computed and compared with the mean of the manual silique length measure. The deviation of the mean length (manual – predicted) is on average of 29 ± 6 pixels, or 2.4 ± 0.5 millimetres. One can observe that the predicted length is systematically longer then the manual one, this can be explained by the difference in the silique length definition. The predicted length has been based on the whole silique object that include a base, a tip and body, whereas the manual measure of silique length tends to discount length of the base and the tip.

Although the correlation between the predicted and manual estimate in mean length is not as high as the one in fruit number estimation, the results are still considered promising with room for improvement. Due to large variation in the length for some images, the "mean length" might not be best to summarise the phenotype; a more robust estimate such as "median" could be used instead. Additionally some rules can be introduced to exclude the extreme or erroneous cases based on the length estimate: e.g. a silique that is too short might not contain any seeds and can be excluded, an extremely long silique object could also be a false detection.
