# Supplementary material for: DeepPod: a convolutional neural network based quantification of fruit number in Arabidopsis
Source: Gigascience. 2020 Mar 4;9(3):giaa012. doi: 10.1093/gigascience/giaa012 (PMC7055469; doi:10.1093/gigascience/giaa012)
Supplement: giaa012_Response_to_Reviewer_Comments_Original_Submission [file giaa012_response_to_reviewer_comments_original_submission.pdf]

Author's Response To Reviewer Comments

Close

Dear Dr. Nogoy:

We are very grateful to the reviewers for their positive and constructive comments on our manuscript. Their valuable suggestions have helped us to improve the manuscript. We have updated the manuscript accordingly and provide below point-by-point responses to the reviewers' comments.

We hope that you find the revised manuscript acceptable for publication in GigaScience.

Thank you for your consideration.

Sincerely,

Chuan Lu, on behalf of the co-authors
Aberystwyth University, UK

Reviewer #1:

Reviewer #1: In this Technical Note the authors report on DeepPod, which is image analysis software that utilises Convolutional Neural Network (CNN) architecture to classify Arabidopsis thaliana plant components into 4 classes. A key feature of the DeepPod image analysis process is the use of patch-based classification to detect and count fruits of this plant. This was followed by the use of CNN-based classifiers to extract patches of interest, and to output the probability that these patches contain any of four components, namely base, body, stem, or tip. The manuscript is well written, and the DeepPod software is available on GitHub with an OSI-approved MIT license that ensures that the software is open and accessible. Images used for manual annotation and training the convolutional neural network, and for testing the performance of the model, are available from a stable DOI link at Aberystwyth University.

- 1. DeepPod software requires Python 2.7, which will not be supported beyond 2020. More details about this are available at the following link: https://pythonclock.org/ As reuse is a major objective of GigaScience, I invite the authors to provide a detailed plan of how they will ensure continued support for DeepPod software in the longer term. For long-term reusability, the authors should consider updating the code to a version of Python 3 that will be supported long term.

Thank you for your comments on this important issue on reusability. We have revised the software requirements for DeepPod that excludes python. The Python scripts (which could be run in Python 3 as well) were only used for generating performance tables for the manuscript, but not required in the DeepPod pipeline. DeepPod requires Caffe, a deep learning framework for CNN classificatin model training and prediction, and Matlab for image annotation, data preparation as well as image reconstruction and silique counting.

Moreover, DeepPod is an ongoing project for which we will provide longer term support and undertake further development both in methodology and software. We believe that DeepPod, as an open source project hosted on GitHub with annotated data freely available, will invite more contributions from the community and make it more sustainable in the future.

- 2. Some of the image files that are publicly available at DOI:10.20391/21154739-f718-457b-96ff-838408f2b696 cannot be opened using ImageJ/Fiji. The authors need to provide md5 checksum values for all image files so that I can identify whether this is a file transfer issue with the 13GB Set-2 dataset, or whether there are corrupt files in the Set-2 directory of 2,408 raw images.

Thank you for your valuable comments. This issue has been sorted with the updated data download

webpage, where md5 checksum values have been provided for both images sets.

- Minor issue 1. The images and readme file on DOI:10.20391/21154739-f718-457b-96ff-838408f2b696 have been ascribed a CC-BY 4.0 license, and therefore GigaScience cannot archive a copy of these data. To ensure reuse, the authors should consider ascribing a CC0 license to these data.

The license from from Set_1 (144 images for model development) and readme file have been changed to CC0. Set-2 had been registered with CC-BY 4.0. All files are available in the Aberystwyth Research Repository with DOI number: 10.20391/21154739-f718-457b-96ff-838408f2b696. Also, Set_1 will be available at GigaDB and a link to Set_2 will be reference in the GigaDB readme.

- 2. There are spaces in the image filenames. These filenames - with spaces - are additionally referred to in the Set-1_Manual_counting.csv and Set-2_manual.csv files. It is the recommendation of the GigaScience Database that filenames do not include spaces (see http://gigadb.org/site/guide).

Fixed. All the spaces from folders and files were replaced with an underline.

Reviewer #2:

In the study, Hamidinekoo et al. developed a deep learning-based method (DeepPod) to count fruit number. Overall, the paper is well written and is easy to follow.
Some minor comments:

- 1) The authors compared the performance of LeNet and DenseNet (Tables 2-5 and Figs 9-10). Is it possible to use the ROC metric show the overall performance?

Table 3 provided the accuracy and loss for the validation data during CNN training, which will not be used as a generalisation performance estimate and therefore no further ROC analysis has been performed on this validation data during training. We have updated Table 4 and 5: besides confusion tables for the four classes, we have also added precision and recall rate for different classes, which should serve as better performance measure compared to accuracy alone. The final overall performance of the pipeline will be evaluated on the silique counting problem using separate test dataset.

- 2) To compare the prediction by LeNet and DenseNet, a similar plot like Fig. 8 is required to show the output of LeNet.

LeNet performed significantly worse than DenseNet on patch classification (total recall of 0.76 and precision of 0.78 on the development test set, in comparison to recall of 0.92 and precision of 0.92 for DenseNet, see Table 4 and 5). Hence only the DenseNet model-based pipeline has been chosen for further evaluation and visual inspection.

- 3) In Figs 9 and 11, it would be useful to add fitted lines and correlation coefficient values in the plots.

Thank you for your valuable suggestion. Figure 9 and 11 have been updated to add fitted lines and the correlation coefficients in the plots.

- 4) How was the manual counting data in Fig 11 obtained? I didn't find relevant information from the main text.

We have added details about manual counting to the end of the section on Data Acquisition (page 3). “Manual counting of viable fruits in images was undertaken by a single person to minimize operator variation. ImageJ [39] was used to track the counting by setting a label to each fruit as it was counted”

Reviewer #3:

This paper describes the development of a deep learning plant phenotyping system. Specifically, the system uses a CNN approach to detecting and counting fruit (seeds/siliques) of the model plant Arabidopsis. A substantial dataset from the National Plant Phenomics Centre is used to build and test the networks. Two network architectures are tested, based on existing formats.

The paper is interesting, but the contribution from the deep learning aspects of the paper are in my opinion small. Two existing (albeit tweaked) network architectures are used and evaluated. One of these is very old. I can not see any novelty in the deep learning application here, so it is either unclear or does not exist. That is not to say there is no novelty in the paper as a whole. The application domain is relevant to plant phenotyping/food security, and I have seen very little existing research on silique detection/counting compared to, say, leaves or roots. The dataset itself is large and will be of value when released. The post-processing steps to reconstruct siliques from patches is novel.

- Major points:
* Why is detecting of the base/stem/body/tip necessary? Why not just detect "siliques" as a whole? This would remove the need for the post processing.

We have now explained our motivation behind the choice of the two-phase approach over the alternative one that detects the siliques as a whole in the last three paragraphs of the Background section. The main reason is that, "training of such networks require labelled data with detailed segmentation or bounding boxes of individual objects, which are obtained usually through a very tedious manual process. Moreover, the image size allowed for the network input is limited due to the complexity of network architecture and the available memory."

There are other reasons for separate detection of the component parts, mainly concerned with future development and refinement of the platform. This will allow us (and hopefully others) to continue investigating alternative approaches that could improve the pipeline for fruit counting and other relevant morphological feature extraction, with more annotated data being available in the future.

- * Following this, the advantage of detecting tips/bases is that silique length can be measured (as mentioned in the future work section). I would suggest this would be a much stronger paper if the processing for doing this was included here. This would justify the detection of sub-features of the siliques, and also provide more novel software development. It would also add much more power to the DeepPod system, making it more useful for the phenotyping community. Given the authors already have the detection system and annotations, only the image processing steps remain to be developed and evaluated.

Thank you for the suggestion. We do have estimates of silique length for all the test examples in Set-2, and have now provided the results (including the mean and range of the silique length) in CSV file as Supplemetary Data S1.

- Minor points:
-LeNet is now a very dated architecture. Please justify better why you are using this as one of the two comparison architectures.

We have added justification for the choice of LeNet architecture to the section on Network Architecture. "LeNet is a simple shallow network with only two convolutional layers, and has been chosen as a baseline model in this study, considering the potentially higher computational resource needs for running more complex deep learning models."

- Was the data collected specifically for this paper or was it already in existence? Please make this more clear. Also, whilst the scanning setup is detailed, descriptions of the growth conditions etc. seem to be missing, and may be helpful if added. Also, I believe all data should be deposited at Gigascience rather than on institution servers (I assume this will happen anyway).

The image data set was pre-existing, having been produced as part of an on-going collaboration with Dr Biernaskie, University of Oxford. The identity of the plant material (i.e. the "RIL" number) is embedded within the image file names, where the first three numbers after the name of the experiment (eg. AT0XX_152XXX) corresponds to a subset of RILs described by Kover et al. 2009. This is now explained both in the paper as well as the on-line DOI. Details of growth conditions have been added (See Data acquisition section, page 3). Dr Biernaskie is now acknowledged in the text. The data will also be available through Gigascience.

- Two datasets are used, referred to as Set-1 (144 images, manually annotated, used for training) and Set-2(2408 images, used for final testing.) Please clarify, was the final test set (Set-2) annotated too, then? The dataset description on p3 makes it sound like only Set-1 is annotated. If it is not annotated,

how exactly is Set-2 used for testing? Later text states "Set-1...was split into train, validation and test sets...". Again it is not clear how Set-2 is going to be used. (To note, further on, on p7, Set-2 is used as a further test set - which means it must be annotated or at least has a manual "inspection" count - please can you detail which of these is the case.)

Set-2 was not annotated to provide labels of silique structural elements (base, tip, stem and body). The structural element labelling was only provided for Set-1 , which was used to train and validate the patch based classification model. However Set-2 has been given manual silique counts for each image. This has been clarified by providing additional details in Table 1 on available annotation and their use in this work for different datasets.

-much of the approach using images patches (including the scanning window, and sub cropping within a larger window for augmentation etc) is similar to our previous work [13]. This is referenced in the introduction, but it might be helpful to also point to this paper in the methods section as there is a lot of similarity in the basic approach.

We have added the reference [13] to the Method section for data preparation and model development, and pointed out that similar approaches have been followed here as in [13] for image patch generation and augmentation.

-Also to note: The authors have referenced our previous work [13] second column p2, but don't quite have the details right. It is not a shallow CNN with 2 x conv layers (please see Supplemental 2 in [13] for full architecture). Please also add details explaining how the approach here is different from the existing approach.

We have revised the manuscript to describe the existing work in [13] in more detail, in particular, to better explain on how the existing approach differs from ours. See the revised paragraph in the Background Section below.

“Pound et al. [13] demonstrated wheat root and shoot feature identification and localisation using two different standard CNN architectures for patch classification. For shoot features, they found that the leaf tips represented the hardest classification problem compared to the leaf base due to the existing variations in orientation, size, shape and colour of tips in their dataset. Further reconstruction from the classification results of overlapping patches allows localisation of separate structural regions such as leaf tips and bases. However, the objects of interest as a whole (such as leaves) are yet to be identified in order to extract more morphological features (e.g. leaf length and shape). ”

-p5. What does the sentence "Note that only annotated patches have been considered for evaluation" mean? Are some not annotated?

Our annotation approach does not require detailed segmentation of the images, only pixel/point sampling for the main structural regions are required. Although most tips and bases have been annotated, only a small portion of patches for body or stems have been labelled (see Figure 3). Also we exclude patches in the background for classification in order to speed up the process for image scanning and reconstruction process. This makes our pipeline actually more cost effective in annotation.

-p5,. "DenseNet showed higher representation learning capacity" - is there evidence for this, or is it a hypothesis?

We have revised the wording to avoid confusion here. “The DenseNet network has higher representational power due to its deeper architecture and its use of features of multiple levels for classification in comparison to the LeNet network; its efficacy in the learning task has also been evidenced by its higher accuracy in classifying plant parts (as shown in Tables 4 and 5).”

- it would be helpful to have a figure (or further supplemental info) illustrating the strategy for dealing with overlapping siliques (p7, "Sillique counting")

Thank you for your valuable suggestion. We have added Supplementary Figure S1 to provide an illustrative example with more details on our strategy for detection and counting siliques with overlapping regions.

-Table 5. It may be more insightful to have some more metrics, e.g. % exactly right, % within 1 count of the groundtruth, % within 5 counts of GT etc. as correlation can be hard to interpret, and is sensitive to outliers (e.g. the the right-most three points in fig 9 may possibly be skewing the correlation)

We have modified Figure 10, the histograms for prediction errors (actual count – predicted count) with finer bins (<5), which will provide a better idea on the distribution of the errors.

- In the results (p7) it seems like a recent non-deep learning approach actually performed better (r^2 0.91 versus 0.9). This definitely warrants further discussion.

We have added more discussion on the non-deep learning approach and how it compares to ours. And their performance of R2=0.91 is only achieved on the cross-validation, when applied on a separate experiment, the R2 actually dropped to 0.7. See below the paragraph added to the Discussion section.

"A recent computer vision approach [2] to fruit number estimation involves linear regression using selected skeleton descriptors (such as junction numbers and number of triple points) extracted after segmentation and 2D skeletonisation, resulted in a Pearson correlation coefficient (R2) of 0.91 between observed and automated values for the best performing model on 100 cross-validated examples. When applied on the dataset from a separate experiment, the model prediction can qualitatively capture the main phenotype under investigation, however the correlation of the prediction with manual counts R2 dropped to 0.7 [2]. This suggests that the regression approach to fruit counting might not be generalized to other conditions as effectively as our object recognition approach. Apparently, the non-deep learning approach used only "handcrafted" global features with resulting models more specific to the conditions for training, whilst our approach used both local features (for patch classification) as well as more global features (for object reconstruction).

-Is the annotation GUI being released?

This toolbox is now registered in the www.SciCrunch.org data sharing and display platform with the Research Resource Identification Initiative ID (RRID) number of "SCR_017413" , under the name of "Plant Phenotyping AnnotationToolbox" . This information is added to the section on source code availability.

-p2 "augment [an] Arabidopsis rosette dataset" (wording)

Fixed.

-p3 "the difference in distribution between testing and training"... please clarify which difference in which distribution you are referring too. (density)

We are referring to the pixel density distribution here. This has been clarified in the revision.

-p3 " (3) to exclude ambiguous patch examples" - sorry I'm not sure of the meaning here.

We have removed this statement in the revision.
--

Close
