# Supplementary material for: DeepPod: a convolutional neural network based quantification of fruit number in Arabidopsis
Source: Gigascience. 2020 Mar 4;9(3):giaa012. doi: 10.1093/gigascience/giaa012 (PMC7055469; doi:10.1093/gigascience/giaa012)
Supplement: giaa012_Response_to_Reviewer_Comments_Revision_1 [file giaa012_response_to_reviewer_comments_revision_1.pdf]

Author's Response To Reviewer Comments

Close

Dear Dr. Nogoy:

We are again very grateful to the reviewers for their positive and constructive comments on our revised manuscript. We have further updated the manuscript accordingly and provide below point-by-point responses to the reviewers' comments.

We hope that you find the second revised manuscript acceptable for publication in GigaScience.

Thank you for your consideration.

Sincerely,

Chuan Lu, on behalf of the co-authors
Aberystwyth University, UK

======

Reviewer #1: The authors have addressed most of my comments, and in particular have updated the software requirements, filenames (to remove spaces), and have ascribed a CC0 license to the Set_1 image data that will be archived in GigaDB. These changes will greatly encourage reuse of this dataset and the supporting scripts. However, I did invite the authors to provide md5 checksum values for all image files (AT023_072342_001.png, AT023_072342_002.png etc) whereas instead they have only provided md5 checksum values for the .tar files. This is a minor point and so I will invite the GigaScience database team to generate a list of checksum values for each and every image file at the point of archiving the Set_1 dataset, and to further invite the authors to check these checksum values at this later date.

- Thank you for the suggestion. We will work with GigaDB team and make sure individual checksum values should be available for Set_1.

Reviewer #2: Thank the authors for addressing my comments. I have no further comments. Congrats to the authors for this nice paper.
- Thank you very much.

Reviewer #3: Dear Authors Thank you for spending time to work through the comments. I am happy that with the corrections the paper is now suitable for publication. However, I have two suggestions to make based on a couple of the revisions:
1. Regarding this revision: Thank you for the suggestion. We do have estimates of silique length for all the test examples in Set-2, and have now provided the results (including the mean and range of the silique length) in CSV file as Supplemetary Data S1.
This is good to see - I wonder if you could manually ground truth just enough of this data to demonstrate the measurements are valid?

- In order to validate the silique length prediction, we manually annotated 32 images randomly selected from Set-2 in more details. In total 2359 siliques were annotated with polylines and have got manual length estimate. Preliminary validation of the mean length estimate has been given in Supplementary Figure S2 and Data S2-S3.

2. Regarding this revision: Our annotation approach does not require detailed segmentation of the images, only pixel/point sampling for the main structural regions are required. Although most tips and bases have been annotated, only a small portion of patches for body or stems have been labelled (see Figure 3).
Could you add some more info to Fig 3 legend to help explain this sampling labelling strategy?

- Done. The Caption in Figure 3 has been modified to explain the annotation procedure in more details.

Close
