# Supplementary material for: DeepPod: a convolutional neural network based quantification of fruit number in Arabidopsis
Source: Gigascience. 2020 Mar 4;9(3):giaa012. doi: 10.1093/gigascience/giaa012 (PMC7055469; doi:10.1093/gigascience/giaa012)
Supplement: giaa012_Response_to_Reviewer_Comments_Revision_2 [file giaa012_response_to_reviewer_comments_revision_2.pdf]

Author's Response To Reviewer Comments

Close

Dear Dr. Nogoy:

We have the addressed the editorial comments to include the reference to the GigaDB supporting data in this revision

Thank you for your support,
Sincerely,
Chuan Lu, on behalf of the co-authors
Aberystwyth University, UK

Close
