# Supplementary material for: DeepPod: a convolutional neural network based quantification of fruit number in Arabidopsis
Source: Gigascience. 2020 Mar 4;9(3):giaa012. doi: 10.1093/gigascience/giaa012 (PMC7055469; doi:10.1093/gigascience/giaa012)
Supplement: giaa012_Reviewer_1_Report_Original_Submission — Chris Armit -- 7/31/2019 Reviewed [file giaa012_reviewer_1_report_original_submission.pdf]

Reviewer Report

Title: DeepPod: A Convolutional Neural Network Based Quantification of Fruit Number in Arabidopsis

Version: Original Submission **Date:** 7/31/2019

Reviewer name: Chris Armit

Reviewer Comments to Author:

In this Technical Note the authors report on DeepPod, which is image analysis software that utilises Convolutional Neural Network (CNN) architecture to classify Arabidopsis thaliana plant components into 4 classes. A key feature of the DeepPod image analysis process is the use of patch-based classification to detect and count fruits of this plant. This was followed by the use of CNN-based classifiers to extract patches of interest, and to output the probability that these patches contain any of four components, namely base, body, stem, or tip. The manuscript is well written, and the DeepPod software is available on GitHub with an OSI-approved MIT license that ensures that the software is open and accessible. Images used for manual annotation and training the convolutional neural network, and for testing the performance of the model, are available from a stable DOI link at Aberystwyth University.
Major issues
1. DeepPod software requires Python 2.7, which will not be supported beyond 2020. More details about this are available at the following link: https://pythonclock.org/
As reuse is a major objective of GigaScience, I invite the authors to provide a detailed plan of how they will ensure continued support for DeepPod software in the longer term. For long-term reusability, the authors should consider updating the code to a version of Python 3 that will be supported long term.
2. Some of the image files that are publicly available at DOI:10.20391/21154739-f718-457b-96ff-838408f2b696 cannot be opened using ImageJ/Fiji. The authors need to provide md5 checksum values for all image files so that I can identify whether this is a file transfer issue with the 13GB Set-2 dataset, or whether there are corrupt files in the Set-2 directory of 2,408 raw images.
Minor issue
1. The images and readme file on DOI:10.20391/21154739-f718-457b-96ff-838408f2b696 have been ascribed a CC-BY 4.0 license, and therefore GigaScience cannot archive a copy of these data. To ensure reuse, the authors should consider ascribing a CC0 license to these data.
2. There are spaces in the image filenames. These filenames - with spaces - are additionally referred to in the Set-1_Manual_counting.csv and Set-2_manual.csv files. It is the recommendation of the GigaScience Database that filenames do not include spaces (see http://gigadb.org/site/guide).

Level of Interest

Please indicate how interesting you found the manuscript: Choose an item.

Quality of Written English

Please indicate the quality of language in the manuscript: Choose an item.

Declaration of Competing Interests

Please complete a declaration of competing interests, considering the following questions:

- Have you in the past five years received reimbursements, fees, funding, or salary from an organisation that may in any way gain or lose financially from the publication of this manuscript, either now or in the future?
- Do you hold any stocks or shares in an organisation that may in any way gain or lose financially from the publication of this manuscript, either now or in the future?
- Do you hold or are you currently applying for any patents relating to the content of the manuscript?
- Have you received reimbursements, fees, funding, or salary from an organization that holds or has applied for patents relating to the content of the manuscript?
- Do you have any other financial competing interests?
- Do you have any non-financial competing interests in relation to this paper?

If you can answer no to all of the above, write 'I declare that I have no competing interests' below. If your reply is yes to any, please give details below.

I declare that I have no competing interests.

I agree to the open peer review policy of the journal. I understand that my name will be included on my report to the authors and, if the manuscript is accepted for publication, my named report including any attachments I upload will be posted on the website along with the authors' responses. I agree for my report to be made available under an Open Access Creative Commons CC-BY license (http://creativecommons.org/licenses/by/4.0/). I understand that any comments which I do not wish to be included in my named report can be included as confidential comments to the editors, which will not be published.

Choose an item.

To further support our reviewers, we have joined with Publons, where you can gain additional credit to further highlight your hard work (see: https://publons.com/journal/530/gigascience). On publication of this paper, your review will be automatically added to Publons, you can then choose whether or not to claim your Publons credit. I understand this statement.

Yes Choose an item.
