# Supplementary material for: DeepPod: a convolutional neural network based quantification of fruit number in Arabidopsis
Source: Gigascience. 2020 Mar 4;9(3):giaa012. doi: 10.1093/gigascience/giaa012 (PMC7055469; doi:10.1093/gigascience/giaa012)
Supplement: giaa012_Reviewer_3_Report_Original_Submission — Andrew French -- 8/21/2019 Reviewed [file giaa012_reviewer_3_report_original_submission.pdf]

Reviewer Report

Title: DeepPod: A Convolutional Neural Network Based Quantification of Fruit Number in Arabidopsis

Version: Original Submission **Date:** 8/21/2019

Reviewer name: Andrew French

Reviewer Comments to Author:

This paper describes the development of a deep learning plant phenotyping system. Specifically, the system uses a CNN approach to detecting and counting fruit (seeds/siliques) of the model plant Arabidopsis. A substantial dataset from the National Plant Phenomics Centre is used to build and test the networks. Two network architectures are tested, based on existing formats.
The paper is interesting, but the contribution from the deep learning aspects of the paper are in my opinion small. Two existing (albeit tweaked) network architectures are used and evaluated. One of these is very old. I can not see any novelty in the deep learning application here, so it is either unclear or does not exist. That is not to say there is no novelty in the paper as a whole. The application domain is relevant to plant phenotyping/food security, and I have seen very little existing research on silique detection/counting compared to, say, leaves or roots. The dataset itself is large and will be of value when released. The post-processing steps to reconstruct siliques from patches is novel.
Major points:
* Why is detecting of the base/stem/body/tip necessary? Why not just detect "siliques" as a whole? This would remove the need for the post processing.
* Following this, the advantage of detecting tips/bases is that silique length can be measured (as mentioned in the future work section). I would suggest this would be a much stronger paper if the processing for doing this was included here. This would justify the detection of sub-features of the siliques, and also provide more novel software development. It would also add much more power to the DeepPod system, making it more useful for the phenotyping community. Given the authors already have the detection system and annotations, only the image processing steps remain to be developed and evaluated.
Minor points:
-LeNet is now a very dated architecture. Please justify better why you are using this as one of the two comparison architectures.
- Was the data collected specifically for this paper or was it already in existence? Please make this more clear. Also, whilst the scanning setup is detailed, descriptions of the growth conditions etc. seem to be missing, and may be helpful if added. Also, I believe all data should be deposited at Gigascience rather than on institution servers (I assume this will happen anyway).
- Two datasets are used, referred to as Set-1 (144 images, manually annotated, used for training) and Set-2(2408 images, used for final testing.) Please clarify, was the final test set (Set-2) annotated too, then? The dataset description on p3 makes it sound like only Set-1 is annotated. If it is not annotated, how exactly is Set-2 used for testing? Later text states "Set-1...was split into train, validation and test sets...". Again it is not clear how Set-2 is going to be used. (To note, further on, on p7, Set-2 is used as a

further test set - which means it must be annotated or at least has a manual "inspection" count - please can you detail which of these is the case.)
-much of the approach using images patches (including the scanning window, and sub cropping within a larger window for augmentation etc) is similar to our previous work [13]. This is referenced in the introduction, but it might be helpful to also point to this paper in the methods section as there is a lot of similarity in the basic approach.
-Also to note: The authors have referenced our previous work [13] second column p2, but don't quite have the details right. It is not a shallow CNN with 2 x conv layers (please see Supplemental 2 in [13] for full architecture). Please also add details explaining how the approach here is different from the existing approach.
-p5. What does the sentence "Note that only annotated patches have been considered for evaluation" mean? Are some not annotated?
-p5,. "DenseNet showed higher representation learning capacity" - is there evidence for this, or is it a hypothesis?
- it would be helpful to have a figure (or further supplemental info) illustrating the strategy for dealing with overlapping siliques (p7, "Sillique counting")
-Table 5. It may be more insightful to have some more metrics, e.g. % exactly right, % within 1 count of the groundtruth, % within 5 counts of GT etc. as correlation can be hard to interpret, and is sensitive to outliers (e.g. the the right-most three points in fig 9 may possibly be skewing the correlation)
-Is Fig 9 the same data as produced Table 5? As the reported r^2 in the legend is different to the table.
- In the results (p7) it seems like a recent non-deep learning approach actually performed better (r^2 0.91 versus 0.9). This definitely warrants further discussion.
-Is the annotation GUI being released?
-p2 "augment [an] Arabidopsis rosette dataset" (wording)
-p3 "the difference in distribution between testing and training"... please clarify which difference in which distribution you are referring too.
-p3 " (3) to exclude ambiguous patch examples" - sorry I'm not sure of the meaning here.

Level of Interest

Please indicate how interesting you found the manuscript: Choose an item.

Quality of Written English

Please indicate the quality of language in the manuscript: Choose an item.

Declaration of Competing Interests

Please complete a declaration of competing interests, considering the following questions:

- Have you in the past five years received reimbursements, fees, funding, or salary from an organisation that may in any way gain or lose financially from the publication of this manuscript, either now or in the future?
- Do you hold any stocks or shares in an organisation that may in any way gain or lose financially from the publication of this manuscript, either now or in the future?
- Do you hold or are you currently applying for any patents relating to the content of the manuscript?
- Have you received reimbursements, fees, funding, or salary from an organization that holds or has applied for patents relating to the content of the manuscript?
- Do you have any other financial competing interests?
- Do you have any non-financial competing interests in relation to this paper?

If you can answer no to all of the above, write 'I declare that I have no competing interests' below. If your reply is yes to any, please give details below.

I declare that I have no competing interests

I agree to the open peer review policy of the journal. I understand that my name will be included on my report to the authors and, if the manuscript is accepted for publication, my named report including any attachments I upload will be posted on the website along with the authors' responses. I agree for my report to be made available under an Open Access Creative Commons CC-BY license (http://creativecommons.org/licenses/by/4.0/). I understand that any comments which I do not wish to be included in my named report can be included as confidential comments to the editors, which will not be published.

Choose an item.

To further support our reviewers, we have joined with Publons, where you can gain additional credit to further highlight your hard work (see: https://publons.com/journal/530/gigascience). On publication of this paper, your review will be automatically added to Publons, you can then choose whether or not to claim your Publons credit. I understand this statement.

Yes Choose an item.
