# Supplementary material for: DeepPod: a convolutional neural network based quantification of fruit number in Arabidopsis
Source: Gigascience. 2020 Mar 4;9(3):giaa012. doi: 10.1093/gigascience/giaa012 (PMC7055469; doi:10.1093/gigascience/giaa012)
Supplement: giaa012_Reviewer_3_Report_Revision_1 — Andrew French -- 12/6/2019 Reviewed [file giaa012_reviewer_3_report_revision_1.pdf]

Reviewer Report

Title: DeepPod: A Convolutional Neural Network Based Quantification of Fruit Number in Arabidopsis

Version: Revision 1 **Date:** 12/6/2019

Reviewer name: Andrew French

Reviewer Comments to Author:

Dear Authors
Thank you for spending time to work through the comments. I am happy that with the corrections the paper is now suitable for publication.
However, I have two suggestions to make based on a couple of the revisions:
1. Regarding this revision:
Thank you for the suggestion. We do have estimates of silique length for all the test examples in Set-2, and have now provided the results (including the mean and range of the silique length) in CSV file as Supplemetary Data S1.
>>This is good to see - I wonder if you could manually ground truth just enough of this data to demonstrate the measurements are valid?
2. Regarding this revision:
Our annotation approach does not require detailed segmentation of the images, only pixel/point sampling for the main structural regions are required. Although most tips and bases have been annotated, only a small portion of patches for body or stems have been labelled (see Figure 3).
>>Could you add some more info to Fig 3 legend to help explain this sampling labelling strategy?
Neither of these are essential, but will make for an improvement to the paper I feel.

Level of Interest

Please indicate how interesting you found the manuscript: Choose an item.

Quality of Written English

Please indicate the quality of language in the manuscript: Choose an item.

Declaration of Competing Interests

Please complete a declaration of competing interests, considering the following questions:

- Have you in the past five years received reimbursements, fees, funding, or salary from an organisation that may in any way gain or lose financially from the publication of this manuscript, either now or in the future?

- Do you hold any stocks or shares in an organisation that may in any way gain or lose financially from the publication of this manuscript, either now or in the future?
- Do you hold or are you currently applying for any patents relating to the content of the manuscript?
- Have you received reimbursements, fees, funding, or salary from an organization that holds or has applied for patents relating to the content of the manuscript?
- Do you have any other financial competing interests?
- Do you have any non-financial competing interests in relation to this paper?

If you can answer no to all of the above, write 'I declare that I have no competing interests' below. If your reply is yes to any, please give details below.

I declare that I have no competing interests

I agree to the open peer review policy of the journal. I understand that my name will be included on my report to the authors and, if the manuscript is accepted for publication, my named report including any attachments I upload will be posted on the website along with the authors' responses. I agree for my report to be made available under an Open Access Creative Commons CC-BY license (http://creativecommons.org/licenses/by/4.0/). I understand that any comments which I do not wish to be included in my named report can be included as confidential comments to the editors, which will not be published.

Choose an item.

To further support our reviewers, we have joined with Publons, where you can gain additional credit to further highlight your hard work (see: https://publons.com/journal/530/gigascience). On publication of this paper, your review will be automatically added to Publons, you can then choose whether or not to claim your Publons credit. I understand this statement.

Yes Choose an item.
